# SingleNucleotide Polymorphisms as Biomarkers of Mepolizumab and Benralizumab Treatment Response in Severe Eosinophilic Asthma

**DOI:** 10.3390/ijms25158139

**Published:** 2024-07-26

**Authors:** Susana Rojo-Tolosa, José Antonio Sánchez-Martínez, Alberto Caballero-Vázquez, Laura Elena Pineda-Lancheros, María Victoria González-Gutiérrez, Cristina Pérez-Ramírez, Alberto Jiménez-Morales, Concepción Morales-García

**Affiliations:** 1 Respiratory Medicine Department, University Hospital Virgen de las Nieves, 18014 Granada, Spain; josebaza@hotmail.com (J.A.S.-M.); alberto.caballero.sspa@juntadeandalucia.es (A.C.-V.); mvglezgut@hotmail.com (M.V.G.-G.); concepcion.morales.sspa@juntadeandalucia.es (C.M.-G.); 2 Pharmacy Service, Pharmacogenetics Unit, University Hospital Virgen de las Nieves, 18014 Granada, Spain; alberto.jimenez.morales.sspa@juntadeandalucia.es; 3 Department of Pharmacy, Faculty of Sciences, National University of Colombia, Bogota Campus, Cra. 30 No. 45-03, Bogotá 11001, Colombia; 4 Center of Biomedical Research, Department of Biochemistry and Molecular Biology II, Institute of Nutrition and Food Technology “José Mataix”, University of Granada, Avda. del Conocimiento s/n., 18016 Granada, Spain; cperezramirez87@ugr.es

**Keywords:** asthma, benralizumab, mepolizumab, personalized medicine, polymorphisms, real life, treatment response

## Abstract

The most promising treatment options for severe uncontrolled asthma (SUA) have emerged in recent years with the development of monoclonal antibodies for blocking selective targets responsible for the underlying inflammation, such as mepolizumab and benralizumab. However, there is variability in treatment response that is not fully controlled. The variability of the response to mepolizumab and benralizumab could be influenced by single-nucleotide polymorphisms (SNPs), and it would be useful to detect these and use them as predictive biomarkers of response. We conducted a retrospective observational cohort study of 72 Caucasian patients recruited from a tertiary hospital with severe uncontrolled eosinophilic asthma treated with mepolizumab and benralizumab. Polymorphisms in the *IL5* (rs4143832, rs17690122), *RAD50* (rs11739623, rs4705959), *IL1RL1* (rs1420101, rs17026974, rs1921622), *GATA2* (rs4857855), *IKZF2* (rs12619285), *FCGR2A* (rs1801274), *FCGR2B* (rs3219018, rs1050501), *FCGR3A* (rs10127939, rs396991), *FCER1A* (rs2251746, rs2427837), *FCER1B* (rs1441586, rs573790, rs569108), and *ZNF415* (rs1054485) genes were analyzed by real-time polymerase chain reaction (PCR) using Taqman probes. The response was analyzed after 12 months of treatment. In patients under mepolizumab treatment, a treatment response defined as a reduction in exacerbations was associated with *ZNF415* rs1054485-T (*p* = 0.042; OR = 5.33; 95% CI = 1.06–30.02), treatment response defined as a reduction in oral corticosteroids use was associated with the number of exacerbations in the previous year (*p* = 0.029; OR = 3.89; 95% CI = 1.24–14.92), and treatment response defined as improvement in lung function was associated with the age at the beginning of biological therapy (*p* = 0.002; OR = 1.10; 95% CI = 1.04–1.18), *FCER1B* rs569108-AA (*p* < 0.001; OR = 171.06; 95% CI = 12.94–6264.11), and *FCER1A* rs2427837-A (*p* = 0.021; OR = 8.61; 95% CI = 1.71–76.62). On the other hand, in patients under benralizumab treatment, treatment response, defined as a reduction in exacerbations, was associated with *ZNF415* rs1054485-T (*p* = 0.073; OR = 1.3 × 10^8^; 95% CI = 1.8 × 10^−19^–NA), *FCER1B* rs569108-AA (*p* = 0.050; OR = 11.51; 95% CI = 1.19–269.78), allergies (*p* = 0.045; OR = 4.02; 95% CI = 1.05–16.74), and sex (*p* = 0.028; OR = 4.78; 95% CI = 1.22–20.63); and treatment response defined as improvement in lung function was associated with polyposis (*p* = 0.027; OR = 9.16; 95% CI = 1.58–91.4), *IKZF2* rs12619285-AA (*p* = 0.019; OR = 9.1; 95% CI = 1.7–75.78), *IL5* rs4143832-T (*p* = 0.017; OR = 11.1; 95% CI = 1.9–112.17), and *FCER1B* rs1441586-C (*p* = 0.045; OR = 7.81; 95% CI = 1.16–73.45). The results of this study show the potential influence of the studied polymorphisms on the response to mepolizumab and benralizumab and the clinical benefit that could be obtained by defining predictive biomarkers of treatment response.

## 1. Introduction

Asthma is a chronic inflammatory disease of the airways involving various cells and cellular mediators. This inflammation causes recurrent episodes of wheezing, dyspnea, chest tightness, and coughing, associated with variable airflow obstruction that is often reversible, either spontaneously or with treatment [1]. The guidelines for asthma management define six therapeutic steps for asthma treatment based on the severity and control of the disease, with the most severe cases placed in the higher steps. Severe uncontrolled asthma (SUA) is characterized by the need to use multiple medications at high doses to manage the condition, typically falling within levels 5–6 of the Guía Española para el Manejo del Asma (GEMA) and level 5 of the Global Initiative for Asthma (GINA) [1,2]. The type 2 immune response plays a central role in the pathogenesis of severe asthma, involving several cell types, in particular type 2 helper T cells (Th2) and type 2 innate lymphoid cells (ILC2) [3].

**Figure 1:**
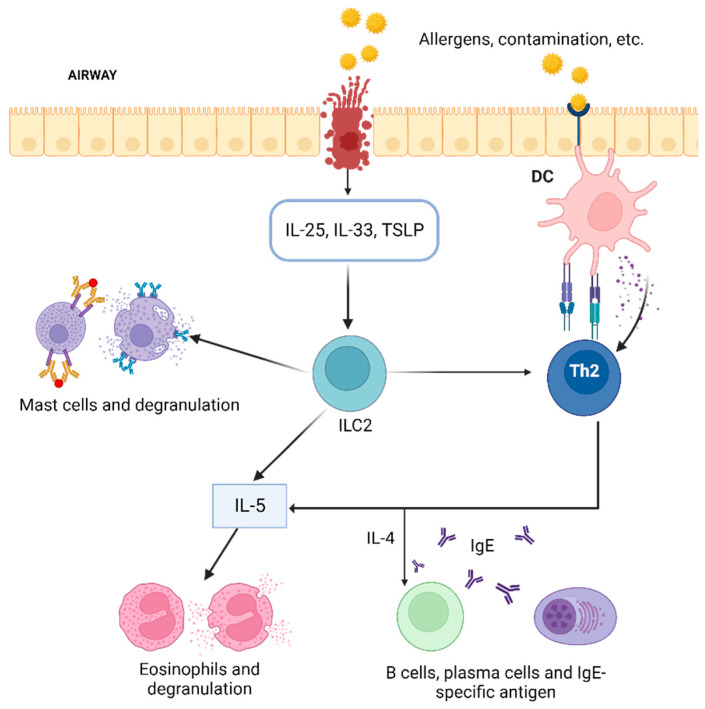
Allergens and environmental pollutants are inhaled and travel to the airways. The arrival of the stimulus in the airway causes damage to the epithelium, resulting in the release of IL-33, IL-25, and TSLP. ILC2, innate immune cells, are activated, release proinflammatory type 2 cytokines such as IL-5 for eosinophil recruitment, and promote degranulation of mast cells. T helper cells are activated in an antigen-dependent manner via dendritic cells. IL: interleukin, Th2: type 2 helper T cells; TSLP: thymic stromal lymphopoietin.

Interleukin (IL)-5 is a potent proinflammatory cytokine responsible for the maturation, proliferation, activation, migration, and survival of eosinophils [4]. It is produced by a variety of cellular components, including Th2 lymphocytes, ILC2 cells, mast cells, and eosinophils. Exposure to allergens triggers activation of Th2 cells, leading to the production and secretion of IL-5. The presence of IL-4 is critical, as it is required for the activation of Th2 cells through the stimulation of key transcription factors. The release of IL-5 from ILC2 cells is dependent on GATA3 activation induced by epithelial innate cytokines such as IL-25, IL-33, and in particular, thymic stromal lymphopoietin (TSLP) (Figure 1) [4].

The most promising treatment options for SUA have emerged in recent years with the development of monoclonal antibodies for blocking IL-5 and its receptor [5].

**Figure 2:**
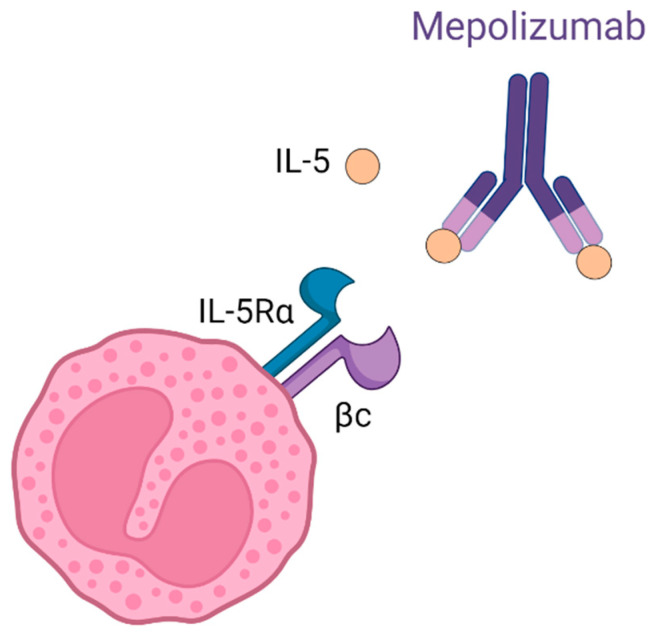
Mechanism of action of mepolizumab. Mepolizumab binds to free IL-5, inhibiting it from binding to the IL-5Rα receptor, thus preventing the eosinophilic cascade from being triggered.

Mepolizumab, marketed as Nucala, is a humanized monoclonal antibody (immunoglobulin (Ig) G1, kappa) that functions by binding to IL-5 with high affinity and specificity [6]. Mepolizumab works by inhibiting the bioactivity of IL-5, which is achieved by blocking the binding of IL-5 to the alpha chain of the IL-5 receptor complex (IL-5Rα) expressed on the surface of eosinophils. Consequently, this inhibition disrupts IL-5 signaling pathways, leading to a reduction in eosinophil production and survival [6] (see Figure 2).

**Figure 3:**
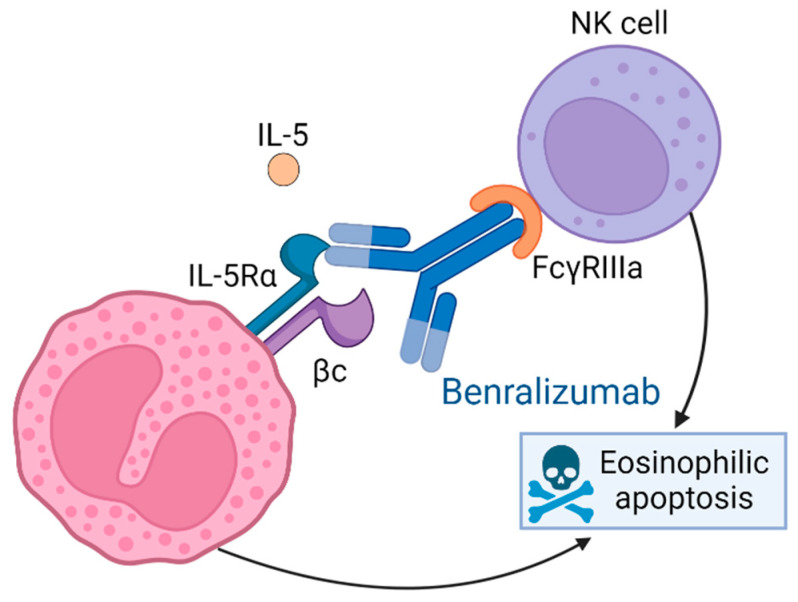
Mechanism of action of benralizumab. Benralizumab specifically binds to the alpha subunit of the IL-5R, thereby preventing IL-5 binding to its receptor and inhibiting eosinophil activation and migration. In addition, the lack of fucose in its constant domain means that it has a high affinity for FcγRIII, such as those on natural killer cells, promoting eosinophilic apoptosis.

Benralizumab, marketed as Fasenra, is a humanized and afucosylated monoclonal antibody (IgG1, kappa) [7]. It binds to IL-5Rα with exceptional affinity and specificity. This receptor is specifically expressed on the surface of eosinophils and basophils. The absence of fucose in the constant (Fc) domain of benralizumab results in high affinity for Fc gamma III receptors (FcγRIII) on immune effector cells such as natural killer (NK) cells [7]. Consequently, this interaction leads to apoptosis of eosinophils and basophils through enhanced antibody-dependent cell-mediated cytotoxicity, ultimately reducing eosinophilic inflammation [7] (see Figure 3).

Mepolizumab and benralizumab are designed to block the action of IL-5 and its receptors. Genetic variation affecting the production or signaling of IL-5 could influence the efficacy of these drugs due to the presence of single-nucleotide polymorphisms (SNPs) in genes that affect both the drug target (*IL5*) and its binding affinity (*FCGR*), as well as other genes that, although not involved in the IL-5 signaling pathway, may affect other signaling pathways. SNPs in non-target genes (*FCER*, *IL1RL1*, *RAD50*, *IKZF2*, *GATA2*) may also be indirectly involved in the IL-5 pathway by affecting other biological processes such as inflammation or the regulation of the immune response, which in turn may modulate the treatment response.

Both monoclonal therapies are IgG type 1. Immunoglobulin molecules consist of two heavy chains and two light chains linked by disulphide bridges. Each chain consists of two components: the Fc domain and a variable region (Fab). The Fc region of IgG is used to improve pharmacokinetics by increasing the stability and prolonging the half-life of the drug [8]. Specifically, the Fc portion of IgG1 binds selectively to FcγR, integral membrane glycoproteins that mediate the complex activation or inhibition of antibody cell function upon IgG aggregation [9,10]. There are several types of FcγR, including FcγRI (CD64), FcγRII (CD32), and FcγRIII (CD16), with different affinities for the Fc region of IgG, thus influencing the response to biologic therapies [9].

Studies have associated SNPs in the *IL5* gene with atopic asthma, increased *IL5* mRNA expression, and decreased lung function and eosinophil count [11,12]. The identification of SNPs in the *IL5* gene that are associated with increased IL-5 expression may indicate the presence of subgroups of asthmatic patients who are more likely to respond to anti-IL-5 therapies. Variations in the amount of IL-5 available may impact the target amount available for these biological therapies to block, and consequently, patients may require higher or more frequent doses to achieve the same therapeutic effect. Furthermore, patients with higher concentrations may have a more pronounced response at the start of treatment, while those with lower levels may show less improvement. These SNPs could also affect the structure of IL-5 and, therefore, its binding affinity to mepolizumab and benralizumab. These SNPs may influence IL-5 signaling pathways, affecting the regulation of the inflammatory response in asthma and other diseases associated with eosinophilia, since IL-5 plays a fundamental role in the maturation and differentiation of eosinophils. Interestingly, eosinophil-specific granules have the capacity to store IL-5, suggesting that these cells may also contribute to elevated circulating levels of IL-5 [13,14]. Some investigators have suggested that RAD50 may act as a general regulator of IL-5 levels. SNPs identified in this gene are strongly associated with IL-5 levels and eosinophilia [15], potentially influencing the therapeutic response.

IL-1 is a proinflammatory cytokine produced by Th2 lymphocytes and its expression is increased in asthma. IL-1 promotes the activation of T lymphocytes, resulting in an increase in the number of eosinophils [16]. Recently, IL-33 has been shown to be a member of the IL-1 family with the ability to induce Th2 responses. Its functions are mediated through IL-1 receptor-like 1 (IL1RL1) signaling, resulting in the release of eosinophils and allergy-related mediators such as IL-5 and IL-13, thereby increasing eosinophil numbers and eosinophilic inflammation [17]. This highlights the potential impact on responses to biologic therapies, particularly mepolizumab and benralizumab.

The *IKZF2* gene encodes a member of the IKAROS family of zinc finger proteins. This family includes hematopoietic-specific transcription factors that are critical for lymphocyte development [18]. Studies have linked the presence of SNPs in this gene to eosinophil counts in asthma [11,19].

The *GATA2* gene encodes a transcription factor that is essential for the development and proliferation of hematopoietic cells and is recognized as a critical regulator of eosinophil development [20]. Similarly to *IKZF2*, studies have associated the presence of SNPs in this gene with eosinophil counts in asthma [11].

The *ZNF415* gene encodes a protein containing multiple zinc finger domains, being involved in the regulation of transcription and cellular function as well as interaction with specific inflammatory pathways [18]. The presence of SNPs in this gene could alter inflammatory, cellular, and transcriptional processes affecting the response of studied biologic therapies.

The high-affinity IgE receptor FcεRI triggers mast cell and basophil activation by interacting with multivalent antigen-bound IgE dimers [21]. The Ag-IgE-FcεRI complex initiates a signaling cascade involving G-protein activation, culminating in the synthesis and release of cytokines such as IL-1, IL-4, and IL-5 [22,23,24,25]. The presence of SNPs in this gene may modulate this process, potentially affecting the response to biological therapies, particularly mepolizumab and benralizumab.

Currently, progress has been made in the search for clinical biomarkers of response to mepolizumab and benralizumab, but there are still important gaps in the knowledge of genetic biomarkers due to the complexity of the immune response, inter-individual variability, limitations of clinical trials, and the limited approach to candidate genes. In this context, the aim of this study was to assess the influence of SNPs in genes involved in the mechanism of action and response process to mepolizumab and benralizumab (*FCGR3, IL-5*, *IL1RL1*, *GATA2, ZNF415, IKZF2*, *RAD50,* and *FCERI*). The aim was to identify predictive biomarkers of response to biologic therapies.

## 2. Results

A retrospective observational cohort study was conducted with 72 patients over 18 years of age of Caucasian origin diagnosed with SUA, according to GEMA 5.2 criteria, between April 2014 and April 2022. The response to mepolizumab was evaluated in 72 patients and to benralizumab in 51 patients before and 12 months after initiation of biologic therapy. Response variables included reduction in OCS courses, improvement in lung function, and reduction in exacerbations. Descriptive and bivariate analyses were performed using R 4.2.0, and multivariate logistic regression was applied to identify prognostic factors for response, controlling for the false discovery rate using the Benjamini-Hochberg method.

### 2.1. Characteristics of the Patients

A total of 72 patients treated with mepolizumab and 51 patients treated with benralizumab were recruited for the study. Clinical and demographic characteristics are shown in Table 1.

### 2.2. Clinical Effectiveness

Clinical efficacy was assessed at 12 months after starting mepolizumab or benralizumab (Table 2). To evaluate the response at 12 months, three response criteria were taken into account: (1) reduction in OCS courses per year, considering a reduction of at least 50% in courses or the absence of OCS as a satisfactory response; (2) improvement in lung function, considering as responses those that achieved an increase of at least 10% in FEV1 or FEV1 > 80% after 12 months of treatment; (3) reduction in exacerbations per year requiring emergency treatment and/or hospitalization, considering a reduction of at least 50% or the absence of exacerbations as a satisfactory response.

As a result, 65.3% (47/72) and 62.8% (32/51) of patients treated with mepolizumab and benralizumab, respectively, responded favorably to the reduction in systemic oral corticosteroids (OCS) by 50% or more. Of the patients, 90.3% (65/72) on mepolizumab and 94.1% (48/51) on benralizumab had reduced numbers of exacerbations in the last 12 months by at least 50%. Additionally, 73.6% (53/72) on mepolizumab and 72.6% (37/51) on benralizumab showed favorable responses in the lung function improvement criterion, defined as an increase in % forced expiratory volume in one second (FEV1) of ≥10% or %FEV1 of ≥80%.

After defining the responses using the three criteria above, 97.2% (70/72) and 98.0% (50/51) of patients responded positively to at least one criterion, 79.2% (57/72) and 82.4% (42/51) responded positively to at least two criteria, and 48.6% (35/72) and 49% (25/51) responded positively to all three response criteria after starting mepolizumab or benralizumab.

### 2.3. Distribution of the Genotypes Analyzed

**Figure 4:**
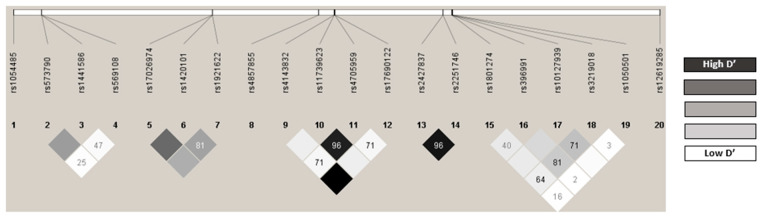
Linkage disequilibrium. The figure shows the linkage disequilibrium between the different pairs of polymorphisms. The darker colors show a strong linkage disequilibrium, while the lighter colors show a low disequilibrium.

The observed genotype frequencies were consistent with those expected according to the Hardy-Weinberg equilibrium (HWE) model, except for *FCGR2A* rs1801274 and *FCGR3A* rs10127939 (*p* = 0.016 and *p* = 0.014, respectively, Table S1). No statistical differences from those described in Iberian populations were found for these variants (*FCGR2A* rs1801274-A allele: 0.653 vs. 0.472; *p* = 0.797 and *FCGR3A* rs10127939-A allele: 0.889 vs. 0.897; *p* = 0.864) [26]. The Lewontin D-prime (D’) and r2 linkage disequilibrium (LD) coefficients are shown in Table S2, and the LD graph is shown in Figure 4. The following pairs of polymorphisms were in strong LD: *IL5* rs1443832/rs17690122 (r2 = 1, D’ = 1), *FCER1A* rs2427837/rs2251746 (r2 = 0.9, D’ = 0.96), and *RAD50* rs11739623/rs4705959 (r2 = 0.84, D’ = 0.96) (Table S2, Figure 4).

All polymorphisms showed a minor allele frequency (MAF) greater than 1%, and therefore, none were excluded from the analysis (Table S3). The estimated haplotype frequencies are shown in Tables S4–S9. The *IL5* haplotype rs1443832/rs17690122 was not associated with any response to mepolizumab or benralizumab (Tables S4 and S7). The CG haplotype of *FCER1A* rs2251746/rs2427837 was associated with responses to all parameters in mepolizumab, except for the response to lung function improvement, which was associated with the GC haplotype (OR = 3.48; 95%CI = 1.07–11.35; *p* = 0.043) (Table S5). The CC haplotype of *RAD50* rs11739623/rs4705959 was associated with the response to mepolizumab when using OCS reduction criterion and when defined as being responsive in terms of all three criteria simultaneously (Table S6). These haplotypes were not significantly associated with the benralizumab response (Tables S8 and S9).

### 2.4. Predictors of Mepolizumab Response at 12 Months

#### 2.4.1. Predictors of Treatment Response Using the Exacerbation Reduction Criterion

Bivariate analysis showed that the absence of previous respiratory disease was significantly associated with a reduction in exacerbations (*p* = 0.047) (see Table S10). Additionally, among the pharmacogenetic variables, carriers of the *ZNF415* rs1054485-T allele showed associations with a reduction in exacerbations (*p* = 0.042) (Table S11).

Further investigation by multivariate analysis showed that the presence of the *ZNF415* rs1054485-T allele emerged as an independent variable significantly associated with a reduction in exacerbations after 12 months of mepolizumab treatment (OR = 5.33; 95% CI = 1.06–30.02) (Table 3).

#### 2.4.2. Predictors of Response for OCS Reduction

In the bivariate analysis, the variable associated with a reduction in OCS use was the absence of exacerbations in the year prior to treatment initiation (*p* = 0.023) (Table S12). However, no significant associations were observed between a reduction in OCS use and any of the genetic variants (Table S13).

In the subsequent multivariate analysis, this association persisted (OR = 3.89; 95% CI = 1.24–14.92) (Table 3).

#### 2.4.3. Predictors of Response for Lung Function Improvement

In bivariate analysis, age at start of mepolizumab treatment was associated with an improvement in lung function (*p* = 0.023) (see Table S14). Regarding pharmacogenetic variables, an association or trend was observed between an improvement in lung function and carriers of the *IL1RL1* rs1420101-T allele for both genotypic and dominant models (*p* = 0.023 and *p* = 0.058, respectively). Associations were also found with the *FCER1B* rs569108-AA genotype (*p* < 0.001), the *FCER1B* rs1441586-TT genotype (*p* = 0.05), and the *FCER1A* rs2427837-A allele for both dominant and genotypic models (*p* = 0.024 and *p* = 0.057, respectively) (Table S15).

Subsequent multivariate analysis revealed independent variables associated with an improvement in lung function after 12 months of mepolizumab treatment, including age at mepolizumab initiation (OR = 1. 10; 95% CI = 1.04–1.18), the *FCER1B* rs569108-AA genotype (OR = 171.06; 95% CI = 12.94–6264.11), and the *FCER1A* rs2427837-A allele (OR = 8.61; 95% CI = 1.71–76.62) (Table 3).

#### 2.4.4. Predictors of Meeting at Least 1 Response Criterion

In the bivariate analysis, none of the sociodemographic or clinical variables showed associations with meeting at least one response criterion (Table S16). However, a statistical association was observed between the response to one criterion and carriers of the *GATA2* rs4857855-C allele (OR = 34; 95% CI = 1.1–1136.7; *p* = 0.026) (Table S17).

In the subsequent multivariate analysis, the independent variable remained associated with meeting at least one criterion after 12 months of mepolizumab treatment (Table 3).

#### 2.4.5. Predictors of Meeting at Least Two Response Criteria

In the bivariate analysis, the variable associated with satisfactory fulfilment of at least two response criteria was the absence of exacerbations in the previous year (*p* = 0.034) (see Table S18). In addition, with regard to pharmacogenetic variables, an association was found between the response to two criteria and the *FCER1B* rs569108-AA genotype for both genotypic and recessive models (*p* = 0.006), as well as the *FCER1A* rs2427837-A allele (*p* = 0.042) (Table S19).

In the subsequent multivariate analysis, the independent variables associated with response to at least two criteria after 12 months of mepolizumab treatment were an absence of exacerbations in the previous year (OR = 13. 79; 95% CI = 2.12–296.89), the *FCER1A* rs2427837-A allele (OR = 4.99; 95% CI = 1.18–30.1), and the *FCER1B* rs569108-AA genotype (OR = 11.86; 95% CI = 1.92–109.30) (Table 3).

#### 2.4.6. Predictors of Meeting All 3 Criteria

In the bivariate analysis, none of the sociodemographic or clinical variables showed an association with meeting all three criteria (Table S20). However, for the pharmacogenetic variables, an association was observed between meeting the criteria, the *IL1RL1* rs1921622-AA genotype (*p* = 0.024), and the *FCER1B* rs569108-AA genotype (*p* = 0.002) (Table S21).

In the subsequent multivariate analysis, the variable that retained the association with meeting all three criteria after 12 months of treatment with mepolizumab was the *IL1RL1* rs1921622-AA genotype (OR = 3.44; 95% CI = 1.18–11.09) (Table 3).

**Table 3:** Predictors of response after 12 months of mepolizumab treatment in patients with severe uncontrolled asthma (multivariate analysis).

	OR (95% CI)	*p* Value	*p* Value ^a^
Response with respect to exacerbation reduction			
*ZNF415* rs1054485 (T vs. GG)	5.33 (1.06–30.02)	0.042	0.042
Response with respect to reduction in OCS			
Exacerbation in previous year (No)	3.89 (1.24–14.92)	0.029	0.029
Response with respect to improved lung function			
Age upon starting mepolizumab (years)	1.10 (1.04–1.18)	0.002	0.003
*FCER1B* rs569108 (AA vs. G)	171.06 (12.94–6264.11)	<0.001	0.003
*FCER1A* rs2427837 (A vs. GG)	8.61 (1.71–76.62)	0.021	0.21
Meeting at least 1 criterion			
*GATA2* rs4857855 (C vs. TT)	34 (1.1–1136.7)	0.026	0.026
Meeting at least 2 criteria			
Exacerbation in previous year (No)	13.79 (2.12–296.89)	0.024	0.036
*FCER1A* rs2427837 (A vs. GG)	4.99 (1.18–30.1)	0.044	0.044
*FCER1B* rs569108 (AA vs. G)	11.86 (1.92–109.31)	0.013	0.036
Meeting all 3 criteria			
*IL1RL1* rs1921622 (AA vs. G)	3.44 (1.18–11.09)	0.028	0.028

OR, odds ratio; 95% CI, 95% confidence interval. ^a^, *p* value for the Benjamini-Hochberg Adjustment Method.

### 2.5. Predictors of Benralizumab Response at 12 Months

#### 2.5.1. Predictors of Treatment Response Using the Exacerbation Reduction Criterion

In the bivariate analysis, the variable that showed a trend towards a reduction in exacerbations was the absence of exacerbations in the previous year (*p* = 0.073) (Table S22). However, no statistical associations were found between a reduction in exacerbations and any of the genetic variants (Table S23).

In the subsequent multivariate analysis, the association of a reduction in exacerbations after 12 months of benralizumab treatment with an absence of exacerbations in the previous year persisted (OR = 1.3 × 10^8^; 95% CI = 1.8 × 10^−19^-NA) (Table 4).

#### 2.5.2. Predictors of Response for OCS Reduction

In the bivariate analysis, several variables were associated with a reduction in OCS, including female gender (*p* = 0.024), presence of allergies (*p* = 0.009), and absence of chronic obstructive pulmonary disease (COPD) (*p* = 0.017) (Table S24). In addition, regarding pharmacogenetic variables, an association or trend was found between a reduction in OCSS and the *FCER1B* rs569108-AA genotype for both genotypic and recessive models (*p* = 0.05) (Table S25).

In the subsequent multivariate analysis, female gender (OR = 4.78; 95% CI = 1.22–20.63), presence of allergies (OR = 4.02; 95% CI = 1.05–16.73), and *FCER1B* rs569108-AA genotype (OR = 11.51; 95% CI = 1.19–269.78) were identified as independent variables associated with a reduction in OCS after 12 months of benralizumab treatment (see Table 4).

#### 2.5.3. Predictors of Response for Lung Function Improvement

According to a bivariate analysis, the variable associated with an improvement in lung function was the presence of nasal polyps (*p* = 0.025), with trends observed for a %FEV1 greater than 80% (*p* = 0.076) and an absence of exacerbations in the previous year (*p* = 0.061) (Table S26). Regarding pharmacogenetic variables, associations or trends were found between an improvement in lung function and the *IKZF2* rs12619285-AA genotype in both recessive and genotypic models (*p* = 0.024 and *p* = 0.061, respectively), the *IL5* rs4143832-T allele (*p* = 0.053), and the *FCER1B* rs1441586-C allele (*p* = 0.074) (Table S27).

In the subsequent multivariate analysis, the independent variables associated with an improvement in lung function after 12 months of treatment with benralizumab were *IL5* rs4143832-T (OR = 11. 1; 95% CI = 1.9–112.17), *IKZF2* rs12619285-AA (OR = 9.10; 95% CI = 1.7–75.78), *FCER1B* rs1441586-C (OR = 7.81; 95% CI = 1.16–73.45), and the presence of nasal polyps (OR = 9.16; 95% CI = 1.58–91.4) (Table 4).

#### 2.5.4. Predictors of Meeting at Least 1 Response Criterion

In the bivariate analysis, no association was found between the clinical, sociodemographic, and genetic variables and meeting at least one criterion for a satisfactory response (Tables S28 and S29).

#### 2.5.5. Predictors of Meeting at Least Two Response Criteria

In the bivariate analysis, the variable associated with meeting at least two criteria was an absence of exacerbations in the previous year (*p* = 0.021), with a trend observed for the presence of nasal polyps (*p* = 0.057) (Table S30). Regarding pharmacogenetic variables, an association was found between the response to two criteria and the *IKZF2* rs12619285-AA genotype for the genotypic model (*p* = 0.038) and the *IKZF2* rs12619285-A allele for the recessive model (*p* = 0.026). (Table S31).

According to a multivariate analysis, the independent variable associated with meeting at least two criteria after 12 months of benralizumab treatment was the *IKZF2* rs12619285-AA genotype (OR = 9.68; 95% CI = 1.58–188.03) (Table 4).

#### 2.5.6. Predictors of Meeting All Three Criteria

The bivariate analysis showed no association between the sociodemographic and clinical variables and the fulfilment of all three criteria (Table S32). However, regarding the pharmacogenetic variables, an association was found between meeting all three criteria and the *IL5* rs4143832-T allele for both genotypic and recessive models (*p* = 0.046 and *p* = 0.014, respectively), as well as the *RAD50* rs4705959-TT genotype (*p* = 0.032) (Table S33).

In the multivariate analysis, the independent variable that remained associated with meeting all three criteria after 12 months of benralizumab treatment was identified as the *IL5* rs4143832-T allele (OR = 4.55; 95% CI = 1.36–17.22) (Table 4).

**Table 4:** Predictors of response after 12 months of benralizumab treatment in patients with severe uncontrolled asthma (multivariate analysis).

	OR (95% CI)	*p* Value	*p* Value ^a^
Response with respect to exacerbation reduction			
Exacerbation in previous year (No)	1.3 × 10^8^ (1.8 × 10^−19^–NA)	0.073	0.073
Response with respect to reduction in OCS			
Sex (Female)	4.78 (1.22–20.63)	0.028	0.050
Allergies (Yes)	4.02 (1.05–16.74)	0.045	0.050
*FCER1B* rs569108 (AA vs. G)	11.51 (1.19–269.78)	0.050	0.050
Response with respect to improved lung function			
*IL5* rs4143832 (T vs. GG)	11.1 (1.9–112.17)	0.017	0.035
*IKZF2* rs12619285 (AA vs. G)	9.1 (1.7–75.78)	0.019	0.035
*FCER1B* rs1441586 (C vs. TT)	7.81 (1.16–73.45)	0.045	0.045
Polyposis (No)	9.16 (1.58–91.4)	0.027	0.035
Meeting at least 1 criterion			
-	-	-	-
Meeting at least 2 criteria			
*IKZF2* rs12619285 (AA vs. G)	9.68 (1.58–188.03)	0.039	0.039
Meeting all 3 criteria			
*IL5* rs4143832 (T vs. GG)	4.55 (1.36–17.22)	0.014	0.014

OR, odds ratio; 95% CI, 95% confidence interval. a, *p* value for the Benjamini-Hochberg Adjustment Method.

## 3. Discussion

The treatment responses of patients diagnosed with SUA characterized by an eosinophilic phenotype are highly variable. Both biologic therapies, mepolizumab and benralizumab, have demonstrated significant efficacy in numerous randomized controlled clinical trials [27,28,29,30,31,32,33] and real-life studies [34,35,36,37,38,39,40,41,42], leading to improvements in symptoms, improved quality of life, and reduced rescue medication use. While previous studies have recognized the substantial genetic contribution to asthma predisposition and response to conventional treatments such as inhaled corticosteroids (ICS) and inhaled short-acting β2 agonists, genetic biomarkers associated with response to these biologic therapies in asthma patients remain unidentified. Therefore, there is a critical need to evaluate the efficacy of these biologic therapies in different populations and to identify biomarkers that can predict their efficacy.

In this study, the treatment response to mepolizumab and benralizumab was assessed after 12 months of treatment. For mepolizumab patients, 90.3%, 73.6%, and 65.3% of the patients were defined as responders based on the “reduction in exacerbations”, “improved lung function”, and “OCS use reduction” criteria, respectively. For benralizumab patients, 94.1%, 72.6%, and 62.8% of patients were defined as responders according to the aforementioned criteria. These results are consistent with those reported in real-world studies, which indicate reductions in OCS courses and exacerbations as well as improvements in lung function [37,38,39,40,41,42]. A systematic review published in 2021 also supported our findings, reporting lower rates of exacerbations requiring emergency department treatment and/or hospitalization, fewer OCS courses, and improved lung function [39].

Although several clinical biomarkers have been proposed as potential predictors of response to mepolizumab and benralizumab, the heterogeneity of asthma requires the use of pharmacogenetics as a complementary tool to advance personalized medicine. The combination of genetic and clinical biomarkers could optimize treatment outcomes by predicting patient response to these biologic therapies. The response to mepolizumab and benralizumab may be influenced not only by SNPs that affect the target of the drug (*IL5*) or its binding affinity (*FCGR*), but also by other genes that, although not involved in the IL-5 signaling pathway, may affect other biological processes that in turn modulate treatment response, such as genes involved in the inflammatory process or the regulation of the immune response (*FCER*, *IL1RL1*, *RAD50, IKZF2*, *GATA2*). However, to date, no study has investigated the role of these or other SNPs in the therapeutic response to mepolizumab or benralizumab [42,43,44,45,46].

Studies have investigated the effect of SNPs within the FcγR, focusing on variants that may affect the stability of the Fc region of IgG1 in the *FCGR2A*, *FCGR2B*, and *FCGR3A* genes, all located on chromosome 1q23.3. We did not find any significant associations between polymorphisms in these genes and the response to mepolizumab and benralizumab. Notably, the *FCGR2A* rs1801274 (A > G) SNP results in a histidine (His) to arginine (Arg) substitution at position 131 (His131Arg) [47,48]. Previous studies have shown that the *FCGR2A*-H131 variant has a lower affinity for IgG1 compared to *FCGR2A*-R131 [49]. Furthermore, the FcγRIIb receptor, which acts as an inhibitor of IgG receptors and has SNPs that have been associated with negative regulation of FcγRIIb expression in B cells and positive regulation in IgG antibody responses [50,51]. With regard to *FCGR3A*, the rs396991 (A > C) SNP results in a substitution of phenylalanine for valine at position 158 (Phe158Val). Previous studies with other biologic therapies have suggested that the low-affinity variant *FCGR3A*-p.158Phe is associated with decreased drug clearance and, consequently, improved therapeutic response [52]. Another polymorphic site within the extracellular domain corresponds to *FCGR3A* rs10127939, located at amino acid residue 66, and exhibits a triallelic variation resulting in a substitution of leucine by either arginine or histidine (L66R/H). This change inhibits glycosylation, potentially affecting the affinity of the ligand for FcγRIIIa [53].

The *IL5* gene is located on chromosome 5q31. Within this gene, two SNPs, namely, rs4143832 (G > T) and rs17690122 (A > G), have been identified to be associated with eosinophil count and IL-5 levels, although their impact on the response to biologic therapies remains uncertain [16]. Our results showed a significant association between the presence of the *IL5* rs4143832-T allele, a favorable response in terms of improvement in lung function, and meeting all three criteria in patients treated with benralizumab.

Regarding the *IL1RL1* gene located on chromosome 2q12, previous studies have suggested that the investigated SNPs (rs1420101, rs17026974, rs1921622) could potentially contribute to the promotion of type 2 inflammatory responses in the airways, characterized by increased eosinophilia, increased serum IgE levels, and decreased FEV1 reversibility. However, their association with the response to biologic therapies has not been thoroughly investigated [47,48,54]. Our research showed a positive correlation between the *IL1RL1* rs1921622-AA genotype and a satisfactory response in terms of lung function improvement in patients treated with mepolizumab.

The *GATA2* gene is situated on chromosome 3q21. Our findings reveal a notable correlation between the presence of the *GATA2* rs4857855-C allele and the fulfillment of at least one criterion among patients undergoing treatment with mepolizumab. Previous research has underscored the pivotal role of *GATA2* as a transcription factor involved in hematopoiesis regulation, particularly in the differentiation of Th2 cells. While this SNP has been linked to elevated eosinophil counts, its impact on the response to biological therapies remains unexplored [49,54].

Moving on to *IKZF2,* located on chromosome 2q13, this gene has not been directly associated with eosinophil biology, but it serves as a regulator in the development and differentiation of lymphocytes through transcriptional control [55]. Our study identifies a significant association between the *IKZF2* rs12619285-AA genotype, a favorable response in terms of lung function improvement, and meeting at least two criteria among patients treated with benralizumab. Although this SNP has previously been linked to high blood eosinophil counts, its influence on the response to biological therapies remains to be elucidated [16].

The *RAD50* gene is located on chromosome 5q31.1. Two investigated SNPs within it, rs11739623 (C > T) and rs4705959 (T > C), have been proposed to potentially influence RAD50 expression, thereby affecting IL-5 and eosinophil levels [20]. Our bivariate analysis suggests that patients treated with benralizumab who carry the *RAD50* rs4705959-TT genotype are more likely to meet all three criteria. Although its association with benralizumab response has not been directly investigated, a previous study showed an association of the *RAD50* rs4705959-T allele with increased eosinophil levels and decreased IL-5 levels, which may explain a more favorable response to benralizumab [20].

On the other hand, the *FCER1A* gene is located on chromosome 1q23. Its association with the response to these therapies has not been studied, but two SNPs within this gene, rs2251746 (T > C) and rs2427837 (G > A), have been associated with increased serum IgE levels [43,44,56]. High serum IgE levels stimulate IgE-FcεRI cross-linking, which triggers the release of pro-inflammatory cytokines such as IL-5 [57]. Our study identified a significant association between carriers of the *FCER1A* rs2427837-A allele, a satisfactory response in terms of improvement in lung function, and meeting at least two criteria in patients treated with mepolizumab.

Within the *FCER1B* gene, located on chromosome 11q12–13, several SNPs have been identified, including rs1441586 (T > C), rs573790 (T > C) and rs569108 (A > G), which are common in asthmatic pathology and atopy and are associated with elevated IgE levels [47,54]. In our study, we observed a significant association between the *FCER1B* rs569108-AA genotype, an increased likelihood of meeting two criteria, and experiencing lung function improvement in patients treated with mepolizumab.

Among patients treated with benralizumab, those with the *FCER1B* rs569108-AA genotype and those with the *FCER1B* rs1441586-C allele had satisfactory responses in terms of reduced oral corticosteroid use and improved lung function, respectively. Although no studies have directly investigated the association of these SNPs with the response to these biologic therapies, it has been reported that the presence of the *FCER1B* rs569108-G and *FCER1B* rs1441586-T alleles correlates with an increased risk of asthma, which could potentially explain the association of the lower-risk alleles with a more favorable prognosis for these biologic therapies [58,59,60,61].

The *ZNF415* gene is located on chromosome 19 and contains the SNP rs1054485 (T > G). In our results, the *ZNF415* rs1054485-T allele was associated with a satisfactory response in terms of fewer exacerbations. In addition, the *ZNF415* rs1054485-G allele has been associated with significantly higher eosinophil counts and asthma [62,63].

Limitations of our study include the possible incomplete collection of relevant clinical data for some patients, which could have affected the accuracy of the results by limiting access to detailed information such as complete medical histories, comprehensive follow-ups of previous treatments, and significant comorbidities. In addition, the small sample size is a major limitation that could have influenced the lack of statistical associations between certain genetic variables and the types of responses analyzed. A small sample size may reduce the ability of a study to detect significant differences, especially in the context of genetic studies that require larger samples to ensure the robustness of the observed associations. However, homogeneity and reliability of the variables collected was ensured by recruiting patients from the same hospital cohort and standardizing procedures and care.

However, although further studies are needed to confirm the results of this study in populations with larger sample sizes and in different geographical areas, it is well known that interindividual genetic variability can contribute to the drug response. Consequently, pharmacogenetics is an important tool that brings us closer to a personalized medicine that, combined with the clinical characteristics and factors associated with the patient, will contribute to obtaining a unique and effective treatment. Personalized medicine has great clinical, economic, and bibliometric impact. First, the patient receives an individualized treatment that allows for maximum effectiveness and low probability of adverse reactions, improving their quality of life. Secondly, health expenditure is often raised by the lack of tools that help to select the most suitable drug for the patient; in this way, having prior response markers would reduce health expenditures in medical care, visits to emergency centers, and hospitalizations. And, thirdly, it could have a strong bibliometric impact, leading to the description of new genetic markers of responses associated, in this case, with biological therapies versus SUA, since there is currently no reference that has described SNPs related to the response to these treatments. Today, the implication of pharmacogenetics is experiencing great advances, with some implications with enough evidence as to establish the determination of SNPs in routine clinical practice. However, there are still few clinical guidelines established; therefore, this research needs to be continued in order for us to be able to continue to take advantage of this tool.

## 4. Materials and Methods

### 4.1. Ethics Statements

The study was conducted with the approval of the Ethics and Research Committee of the Hospital Universitario Virgen de las Nieves, in accordance with the Declaration of Helsinki (1313-N-20). The subjects who participated in the study signed informed consent forms for the collection and genetic analysis of saliva samples and for their donation to the Biobank of the Sistema Sanitario Público de Andalucía (Andalusian Public Health Service). The samples were identified using alphanumeric codes.

### 4.2. Study Design

We conducted an observational, retrospective cohort study.

### 4.3. Study Population

This study included 72 patients aged over 18 years and of Caucasian origin who were diagnosed with SUA according to the criteria of the GEMA 5.2 [1], who had received mepolizumab or benralizumab, and who had been recruited in the Respiratory Medicine Department of the Hospital Universitario Virgen de las Nieves in Granada (Spain) between April 2014 and April 2022. Of the patients recruited, the response to mepolizumab was evaluated in 72 patients and that to benralizumab in 51 patients before beginning treatment as well as 12 months after the start of biological therapy. The administration route of the drug was subcutaneous: 100 mg of mepolizumab every 4 weeks and 30 mg of benralizumab every 4 weeks for the first 3 doses, then subsequently every 8 weeks [6,7].

### 4.4. Socio-Demographic and Clinical Variables

The socio-demographic and clinical data were collected by reviewing clinical histories. The socio-demographic data collected were age, sex, body mass index (BMI), and smoking status. The clinical variables included years with the disease, nasal polyposis, previous respiratory disease, allergies, gastroesophageal reflux disease (GERD), sleep apnea-hypopnea syndrome (SAHS), COPD, years of biological therapy, treatment dose, change to another biological therapy, courses of oral corticosteroids (OCS) expressed as prednisone-equivalent mg and of ICS expressed as fluticasone furoate-equivalent µg, blood eosinophil count, exacerbations requiring emergency department treatment and/or hospitalization, IgE, lung function as maximum percentage expiratory volume in the first second of forced exhalation (%FEV1), and Asthma Control Test (ACT) results [64]. The %FEV1 was calculated by comparing the measured value of the patient’s forced expiratory volume in the first second (FEV1) with the reference value predicted for a person of the same age, sex, height, and ethnicity. The clinical variables were collected with reference to the year before starting biological therapy and after completing the first year of treatment.

### 4.5. Genetic Variables

#### 4.5.1. DNA Isolation

The saliva samples were collected via buccal swabs (OCR-100 Kit). The DNA was extracted using the QlAamp DNA Mini Kit (Qiagen GmbH, Hilden, Germany), following the manufacturer’s instructions for purifying DNA from saliva, and was stored at −40 °C. The concentration and purity of the DNA were measured using a NanoDrop 2000 UV spectrophotometer with 280/260 and 280/230 absorbance ratios.

#### 4.5.2. Detection of Gene Polymorphisms and Quality Control

The gene polymorphisms were determined by real-time polymerase chain reaction (PCR) allelic discrimination assay using TaqMan probes (ABI Applied Biosystems, QuantStudio 3 Real-Time PCR System, 96 wells) according to the manufacturer’s instructions (Table 5). The *FCGR2B* rs3219018 and *FCGR2B* rs1050501 polymorphisms were analyzed using a custom assay by ThermoFisher Scientific (Waltham, Massachusetts, United States) and coded as ANPRZAZ and ANRWUVX, respectively. Ten percent of the results were confirmed by Sanger sequencing. Real-time PCR and Sanger sequencing were performed in the Pharmacogenetics Unit of the Hospital Universitario Virgen de las Nieves. The criteria for SNP quality control were: (1) missing genotype rate per SNP < 0.05; (2) minor allele frequency > 0.01; (3) *p* value > 0.05 in Hardy-Weinberg equilibrium test; and (4) missing genotype rate between cases and control < 0.05.

### 4.6. Response Variables

To evaluate the predictors of response at 12 months, the following were taken as response variables: reduction in OCS courses per year, considering a reduction of at least 50% in courses or absence of OCS as a satisfactory response; improvement of lung function, considering those that achieved an increase of at least 10% in FEV1 or FEV1 > 80% after 12 months’ treatment as responders; and reduction in exacerbations per year requiring emergency department treatment and/or hospitalization, taking a reduction of at least 50% or absence of exacerbations as a satisfactory response.

On the other hand, global response was also evaluated, taking into account the variables previously described as the 3 possible response criteria and considering (1) patients who responded positively to at least 1 response criterion; (2) patients who responded positively to at least 2 response criteria; and (3) patients who responded positively to all 3 response criteria.

### 4.7. Statistical Analysis

The descriptive analysis was performed with R 4.2.0 software. The quantitative variables were expressed as the mean (±standard deviation) for those that complied with normality and as the median and percentile (25 and 75) for the variables that did not follow a normal distribution. Normality was confirmed using the Kolmogorov-Smirnov test. The bivariate analysis between the response and the genetic variables was performed with multiple models—genotypic (DD vs. dd and Dd vs. dd), dominant ((DD, Dd) vs. dd), and recessive (DD vs. (Dd, dd))—using the Pearson χ^2^ test or applying the Fisher exact test for the qualitative variables. The Pearson χ^2^ test was used for those samples whose expected frequencies in the contingency table were greater than or equal to 5, and the Fisher exact test was used for those samples with expected frequencies lower than 5. For the quantitative variables, we applied the *t* test to the variables that complied with normality and the Mann-Whitney U test for non-normal variables.

A multivariate (logistic regression) analysis was used to calculate the adjusted odds ratio (OR) and the 95% confidence interval (95% CI) for the possible prognostic factors for response. For the selection of the variables included in the multivariate model, the “backward stepwise” technique was used, which considers a value of *p* < 0.05 as an input criterion. The Benjamini-Hochberg method was used to control the false discovery rate (FDR) when performing multiple comparisons.

The Hardy-Weinberg equilibrium, the haplotype frequency, and the linkage disequilibrium (LD) were determined through the Lewontin D-prime (D’) coefficients and the LD coefficient (r2).

All the tests were 2-sided, with significance levels of *p* < 0.05, and were performed using PLINK 1.9 free-access software for whole-genome association analysis [65] and the R 4.2.0 statistical program [66]. The LD was calculated with Haploview 4.2 software [67], and the haplotype analysis was performed with SNPStats [68], a web tool for the analysis of association studies.

## 5. Conclusions

In conclusion, this study shows that polymorphisms in the target gene and the mechanism of action of mepolizumab and benralizumab may have potential prognostic value for treatment response. The identification of genetic biomarkers of response to mepolizumab and benralizumab may allow for personalization of treatment, providing a clinical tool to select the most appropriate therapy based on the patient’s genetic profile. In this way, clinical resources could be optimized; costs could be reduced; and, by predicting the patient’s response, a better therapeutic response and consequently a better quality of care could be achieved. However, the functional involvement of most asthma loci is still unknown. Further studies are therefore needed to improve our understanding of the impact of these genes and their effects on response to biologic therapies in order to enable their use in future clinical practice and to guide us towards more personalized medicine.

## Figures and Tables

**Table 1:** Socio-demographic and clinical characteristics of patients treated with mepolizumab and benralizumab.

	Mepolizumab		Benralizumab	
Variables	N	Cases (N = 72)	N	Cases (N = 51)
**Sex**				
Women	48	66.7	34	66.7
Men	24	33.3	17	33.3
**Age upon starting biological therapy (years)**	72	53 ± 13	51	58 ± 15
**Years with asthma**	72	7 (3, 10)	51	6 (4, 10)
**BMI (kg/m^2^)**				
<25	19	26.4	9	17.7
>25	53	73.6	42	82.3
**Previous respiratory disease**				
Yes	34	47.2	24	47.1
No	38	52.8	27	52.9
**Smoking status**				
Non-smoker	12	16.7	39	76.5
Active smoker	0	0	2	3.9
Ex-smoker	60	83.3	10	19.6
**Polyposis**				
Yes	33	45.8	20	39.2
No	39	54.2	31	60.8
**Allergies**				
Yes	37	51.4	33	64.7
No	35	48.6	18	35.3
**GERD**				
Yes	32	44.4	22	43.1
No	40	55.6	29	56.9
**SAHS**				
Yes	15	20.8	10	19.6
No	57	79.2	41	80.4
**COPD**				
Yes	13	18.1	10	19.6
No	59	81.9	41	80.4
**Age at diagnosis (years)**	72	49 ± 15	51	52 ± 15
<18	2	2.8	1	2
>18	70	97.2	50	98
**ICS (µg/day)**	72	500 (500, 1000)	51	1000 (500, 1000)
**OCS courses per year**				
Yes	57	79.2	45	88.2
No	15	20.8	6	11.8
**Baseline %FEV1**				
<80	51	70.8	34	66.7
>80	21	29.2	17	33.3
**Exacerbation in previous year**				
Yes	47	65.3	22	43.1
No	25	34.7	29	56.9
**Baseline blood eosinophils (cells/µL)**				
<300	15	20.8	21	41.2
>300	57	79.2	30	58.8
**Previous biological therapy**				
Yes	21	29.2	20	39.2
No	51	70.8	31	60.8

%FEV1, maximum percentage expiratory volume in the first second of forced expiration; BMI, body mass index; COPD, chronic obstructive pulmonary disease; GERD, gastroesophageal reflux disease; ICS, inhaled corticosteroids; OCS, oral corticosteroids; SAHS, sleep apnea-hypopnea syndrome. Qualitative variables are shown as numbers (percentage, %). Quantitative variables with normal distribution are shown as mean ± standard deviation (SD). Quantitative variables with non-normal distribution are shown as medians (interquartile range).

**Table 2:** Clinical effectiveness of mepolizumab and benralizumab in patients with severe uncontrolled asthma and eosinophilic phenotype.

Response Variable	Mepolizumab		Benralizumab	
	N	%	N	%
Reduction in OCS ≥ 50%				
R	47	65.3	32	62.8
NR	25	34.7	19	37.2
Reduction in exacerbations ≥ 50%				
R	65	90.3	48	94.1
NR	7	9.7	3	5.9
Increase in %FEV1 ≥ 10% or %FEV1 ≥ 80%				
Yes	53	73.6	37	72.6
No	19	26.4	14	27.4
Responsive for 1 criterion				
R	70	97.2	50	98.0
NR	2	2.8	1	2.0
Responsive for 2 criteria				
R	57	79.2	42	82.4
NR	15	20.8	9	17.6
Responsive for 3 criteria				
R	35	48.6	25	49
NR	37	51.4	26	51

%FEV1, maximum percentage expiratory volume in the first second of forced expiration; OCS, oral corticosteroids; R, responsive; NR, non-responsive. Qualitative variables are shown as numbers (percentage, %).

**Table 5:** Gene polymorphisms and TaqMan ID.

Gene	SNP	dbSNP ID	Assay ID
*IL5* (5q31)	T > G	rs4143832	C__28028637_10
	A > G	rs17690122	C__33291516_10
*RAD50* (5q31.1)	C > T	rs11739623	C__31237883_10
	T > C	rs4705959	C___2549990_10
*FCER1A* (1q23)	T > C	rs2251746	C___1840470_20
	G > A	rs2427837	C__16233438_20
*FCER1B* (11q12–13)	T > C	rs1441586	C___1842226_10
	T > C	rs573790	C____900105_20
	A > G	rs569108	C____900116_10
*ZNF415* (19q)	T > G	rs1054485	C___2932371_10
*IKZF22* (2q13)	A > G	rs12619285	C___1861821_10
*FCGR2A* (1q23.3)	A > G	rs1801274	C___9077561_20
*FCGR2B* (1q23.3)	G > C	rs3219018	ANPRZAZ
	T > C	rs1050501	ANRWUVX
*FCGR3A* (1q23.3)	A > C	rs10127939	C__57480226_10
	A > C	rs396991	C__25815666_10
*IL1RL1* (2q12)	C > T	rs1420101	C___8906009_20
	G > A	rs17026974	C__33551182_10
	G > A	rs1921622	C___1226146_10
*GATA2* (3q21)	C > T	rs4857855	C__11231076_10

## Data Availability

Data are contained within the article and Supplementary Materials.

## Supplementary Materials

The following supporting information can be downloaded at: https://www.mdpi.com/article/10.3390/ijms25158139/s1.

## Abbreviations

ACT	Asthma Control Test
BMI	Body Mass Index
CI	Confidence Interval
COPD	Chronic Obstructive Pulmonary Disease
FEV1	Forced Expiratory Volume in one second
GEMA	Guía Española para el Manejo del Asma
GERD	Gastroesophageal Reflux Disease
GINA	Global Initiative for Asthma
HWE	Hardy-Weinberg Equilibrium
ICS	Inhaled Corticosteroids
Ig	Immunoglobulin
IL	Interleukin
ILC2	Type 2 Innate Lymphoid Cells
LD	Linkage Disequilibrium
MAF	Minor Allele Frequency
NK	Natural Killer
OCS	Oral Corticosteroids Systemic
OR	Odds Ratio
PCR	Polymerase Chain Reaction
SAHS	Sleep Apnea-Hypopnea Syndrome
SD	Standard Deviation
SNP	Single-Nucleotide Polymorphisms
SUA	Severe Uncontrolled asthma
Th2	Type 2 Helper T
TSLP	thymic stromal lymphopoietin
