# Supplementary material for: SingleNucleotide Polymorphisms as Biomarkers of Mepolizumab and Benralizumab Treatment Response in Severe Eosinophilic Asthma
Source: Int J Mol Sci. 2024 Jul 26;25(15):8139. doi: 10.3390/ijms25158139 (PMC11311889; doi:10.3390/ijms25158139)
Supplement: Supplementary file 1 [file ijms-25-08139-s001.zip › Table S1.pdf]

Table S1. Hardy-Weinberg equilibrium for the SNPs included in the study.

CHR	SNP	Gene	Minor allele	Major allele	Genotype counting	Observed heterozygosity	Expected heterozygosity	p-valor
1	rs2427837	ALL	A	G	6/25/41	0.3472	0.3818	0.5345
1	rs2251746	ALL	C	T	5/26/41	0.3611	0.375	0.7556
1	rs1801274	ALL	G	A	20/25/27	0.3472	0.4953	0.01631
1	rs396991	ALL	C	A	9/41/22	0.5694	0.4837	0.2211
1	rs10127939	ALL	C	A	3/8/61	0.1111	0.1755	0.01372
1	rs3219018	ALL	C	G	1/24/47	0.3333	0.2959	0.4406
1	rs1050501	ALL	C	T	1/16/55	0.2222	0.2188	1
2	rs17026974	ALL	A	G	6/28/38	0.3889	0.4012	0.7728
2	rs1420101	ALL	T	C	12/34/26	0.4722	0.4811	1
2	rs1921622	ALL	G	A	13/39/20	0.5417	0.4953	0.4845
2	rs12619285	ALL	G	A	5/31/36	0.4306	0.4073	0.7761
3	rs4857855	ALL	T	C	3/16/53	0.2222	0.2589	0.1987
5	rs4143832	ALL	T	G	4/17/51	0.2361	0.2869	0.2035
5	rs11739623	ALL	T	C	2/32/38	0.4444	0.375	0.2051
5	rs4705959	ALL	C	T	3/28/41	0.3889	0.3607	0.7447
5	rs17690122	ALL	G	A	4/17/51	0.2361	0.2869	0.2035
11	rs573790	ALL	T	C	6/36/30	0.5	0.4444	0.4259
11	rs1441586	ALL	C	T	11/41/20	0.5694	0.4922	0.2357
11	rs569108	ALL	G	A	0/9/63	0.125	0.1172	1
19	rs1054485	ALL	G	T	17/31/24	0.4306	0.4953	0.3396

CHR, chromosome; SNP, single nucleotide polymorphism.
