# Supplementary material for: SingleNucleotide Polymorphisms as Biomarkers of Mepolizumab and Benralizumab Treatment Response in Severe Eosinophilic Asthma
Source: Int J Mol Sci. 2024 Jul 26;25(15):8139. doi: 10.3390/ijms25158139 (PMC11311889; doi:10.3390/ijms25158139)
Supplement: Supplementary file 1 [file ijms-25-08139-s001.zip › Table S10.pdf]

Table S10. Association of clinical characteristics of mepolizumab-treated patients with reduced and/or no exacerbations.

Characteristics	N	Response R N (%)	NR N (%)	X^2	p-value	Ref. Cat	OR	CI 95%
Sex								
Female	48	41 (85.4)	7 (14.6)		0.087*			
Male	24	24 (100)	0 (0)					
Age of initiation BT (years)	72	65 (90.3)	7 (9.7)		0.833			
Years with asthma	72	65 (90.3)	7 (9.7)		0.777			
BMI (kg/m2)								
<25	19	18 (94.7)	1 (5.3)	0.5847	0.445			
>25	53	47 (88.7)	6 (11.3)					
Previous respiratory disease								
Yes	34	28 (2.4)	6 (17.6)		0.047*	Si	7.93	1.26-154.29
No	38	37 (97.4)	1 (2.6)					
Tobacco consumption								
Non smoker	60	53 (88.3)	7 (11.7)	1.5508	0.213			
Current smoker	0	0 (0)	0 (0)					
Former smoker	12	12 (100)	0 (0)					
Polyps								
Yes	33	29 (87.9)	4 (12.1)		0.695*			
No	39	36 (92.3)	3 (7.7)					
Allergies								
Yes	37	32 (86.5)	5 (13.5)		0.430*			
No	35	33 (94.3)	2 (5.7)					
GERD								
Yes	32	29 (90.6)	3 (9.4)		1*			
No	40	36 (90)	4 (10)					
SAHS								
Yes	15	15 (100)	0 (0)	2.0405	0.153			
No	57	50 (97.7)	7 (12.3)					
COPD								
Yes	13	11 (84.6)	2 (15.4)	0.5795	0.447			
No	59	54 (91.5)	5 (8.5)					
Age of diagnosis (years)								
<18	2	2 (100)	0 (0)		1*			
>18	70	63 (90)	7 (10)					
ICS (µg/day)	72	65 (90.3)	7 (9.7)		0.206			
OCS cycles per year								
Yes	57	52 (91.2)	5 (8.8)	0.2815	0.596			
No	15	13 (86.7)	2 (13.3)					
Baseline FEV1 (%)								
<80	51	45 (88.2)	6 (11.8)		0.665*			
>80	21	20 (95.2)	1 (4.8)					
Exacerbation in previous year								
Yes	47	41 (87.2)	6 (12.8)		0.409*			
No	25	24 (96)	1 (4)					
Basal blood eosinophils (cell/mcl)								
<300	15	13 (86.7)	2 (13.3)	0.2815	0.296			
>300	57	52 (91.2)	5 (8.8)					
Previous BT								
Yes	21	17 (81)	4 (19)		0.183*			
No	51	48 (94.1)	3 (5.9)					
