# Supplementary material for: SingleNucleotide Polymorphisms as Biomarkers of Mepolizumab and Benralizumab Treatment Response in Severe Eosinophilic Asthma
Source: Int J Mol Sci. 2024 Jul 26;25(15):8139. doi: 10.3390/ijms25158139 (PMC11311889; doi:10.3390/ijms25158139)
Supplement: Supplementary file 1 [file ijms-25-08139-s001.zip › Table S11.pdf]

Table S11. Association of mepolizumab genetic polymorphisms with reduction and/or absence of exacerbations.

Gene	SNPs	Genotype	N	Response		χ²	p-value	Ref Cat	OR	CI 95%
				R<br>N (%)	NR<br>N (%)					
		CC	26	25 (96.2)	1 (3.8)					
		CT	34	30 (88.2)	4 (11.8)		0.267*			
	rs1420101	TT	12	10 (83.3)	2 (16.7)					
		C	60	55 (91.7)	5 (8.3)	0.7912	0.378			
		T	46	40 (87)	6 (13)		0.41*			
		AA	6	5 (83.3)	1 (16.7)					
		AG	28	25 (89.3)	3 (10.7)		0.476*			
IL1RL1	rs17026974	GG	38	35 (92.1)	3 (7.9)					
		A	34	30 (88.2)	4 (11.8)		0.7*			
		G	66	60 (90.9)	6 (9.1)	0.3595	0.549			
		AA	20	18 (90)	2 (10)					
		AG	39	34 (87.2)	5 (12.8)		0.49*			
	rs1921622	GG	13	13 (100)	0 (0)					
		A	59	52 (88.1)	7 (11.9)	1.7085	0.191			
		G	52	47 (90.4)	5 (9.6)	0.0024	0.961			
		GG	51	46 (90.2)	5 (9.8)					
		GT	17	15 (88.2)	2 (11.8)		1*			
	rs4143832	TT	4	4 (100)	0 (0)					
		G	68	61 (89.7)	7 (10.3)		1*			
		T	21	19 (90.5)	2 (9.5)					
IL5		AA	51	46 (90.2)	5 (9.8)					
		AG	17	15 (88.2)	2 (11.8)		1*			
	rs17690122	GG	4	4 (100)	0 (0)					
		A	68	61 (89.7)	7 (10.3)		1*			
		G	21	19 (90.5)	2 (9.5)		1*			
		CC	53	47 (88.7)	6 (11.3)					
		CT	16	16 (100)	0 (0)		0.136*			
GATA2	rs4857855	TT	3	2 (66.7)	1 (33.3)					
		C	69	63 (91.3)	6 (8.7)		0.268*			
		T	19	18 (94.7)	1 (5.3)	0.5847	0.445			
		AA	36	31 (86.1)	5 (13.9)					
		AG	31	29 (93.5)	2 (6.5)		0.668*			
IKZF2	rs12619285	GG	5	5 (100)	0 (0)					
		A	67	60 (89.6)	7 (10.4)		1*			
		G	36	34 (94.4)	2 (5.6)		0.429*			

Gene	SNPs	Genotype	N	Response		χ²	p-value	Ref Cat	OR	CI 95%
				R N (%)	NR N (%)					
RAD50	rs11739623	CC	38	33 (86.8)	5 (13.2)		0.558*			
		CT	32	30 (93.8)	2 (6.2)					
		TT	2	2 (100)	0 (0)					
		C	70	63 (90)	7 (10)		1*			
		T	34	32 (94.1)	2 (5.9)		0.435*			
	rs4705959	CC	3	3 (100)	0 (0)		0.775*			
		CT	28	26 (92.9)	2 (7.1)					
		TT	41	36 (87.8)	5 (12.2)					
		C	31	29 (92.5)	2 (6.5)		0.691*			
		T	69	62 (89.9)	7 (10.1)		1*			
FCER1A	rs2251746	CC	5	4 (80)	1 (20)		0.656*			
		CT	26	24 (92.3)	2 (7.7)					
		TT	41	37 (90.2)	4 (9.8)					
		C	31	28 (90.3)	3 (9.7)		1*			
		T	67	61 (91)	6 (9)		0.41*			
	rs2427837	AA	6	5 (83.3)	1 (16.7)		0.374*			
		AG	25	24 (96)	1 (4)					
		GG	41	36 (87.8)	5 (12.2)					
		A	31	29 (93.5)	2 (6.5)		0.415*			
		G	66	60 (90.9)	6 (9.1)	0.3596	0.549			
FCER1B	rs1441586	CC	11	9 (81.8)	2 (18.2)		0.479*			
		CT	41	38 (92.7)	3 (7.3)					
		TT	20	18 (90)	2 (10)					
		C	52	47 (90.4)	5 (9.6)	0.0024	0.961			
		T	61	56 (91.8)	5 (8.2)	1.0586	0.304			
	rs573790	CC	30	28 (93.3)	2 (6.7)		0.587*			
		CT	36	31 (86.1)	5 (13.9)					
		TT	6	6 (100)	0 (0)					
		C	66	59 (89.4)	7 (10.6)	0.7049	0.401			
		T	42	37 (88.1)	5 (11.9)		0.692*			
	rs569108	AA	63	57 (90.5)	6 (9.5)	0.0226	0.881			
		AG	9	8 (88.9)	1 (11.1)					
		GG	0	0 (0)	0 (0)					
		A	72	65 (90.3)	7 (9.7)	-	-			
		G	9	8 (88.9)	1 (11.1)	0.0226	0.881			

Gene	SNPs	Genotype	N	Response		χ²	p-value	Ref Cat	OR	CI 95%
				R<br>N (%)	NR<br>N (%)					
ZNF415	rs1054485	GG	17	13 (76.5)	4 (23.5		0.144*			
		GT	31	29 (93.5)	2 (6.5)					
		TT	24	23 (95.8)	1 (4.2)					
		G	48	42 (87.5)	6 (12.5)		0.412*			
		T	55	52 (94.5)	3 (5.5)	4.8337	0.028	GG	5.33	1.06-30.02
FCER1G	rs11587213	AA	55	49 (89.1)	6 (10.9)		1*			
		AG	12	11 (91.7)	1 (8.3)					
		GG	5	5 (100)	0 (0)					
		A	67	60 (89.6)	7 (10.4)		1*			
		G	17	16 (94.1)	1 (5.9)	0.3739	0.541			
C3	rs2230199	CC	3	3 (100)	0 (0)		1*			
		CG	26	23 (88.5)	3 (11.5)					
		GG	43	39 (90.7)	4 (9.3)					
		C	29	26 (89.7)	3 (10.3)		1*			
		G	69	62 (89.9)	7 (10.1)		1*			
FCGR2A	rs1801274	AA	27	24 (88.9)	3 (11.1)		1*			
		AG	25	23 (92)	2 (8)					
		GG	20	18 (90)	2 (10)					
		A	52	47 (90.4)	5 (9.6)	0.0024	0.961			
		G	45	41 (91.1)	4 (8.9)		1*			
FCGR2B	rs3219018	CC	1	1 (100)	0 (0)		0.293*			
		CG	24	20 (83.3)	4 (16.7)					
		GG	47	44 (93.6)	3 (6.4)					
		C	25	21 (84)	4 (16)		0.227*			
		G	71	64 (90.1)	7 (9.9)		1*			
	rs1050501	CC	1	1 (100)	0 (0)		1*			
		CT	16	15 (93.8)	1 (6.2)					
		TT	55	49 (89.1)	6 (10.9)					
		C	17	16 (94.1)	1 (5.9)	0.3739	0.541			
		T	71	64 (90.1)	7 (9.9)		1*			
FCGR3A	rs10127939	AA	61	56 (91.8)	5 (8.2)		0.402*			
		AC	8	6 (75)	2 (25)					
		CC	3	3 (100)	0 (0)0					
		A	69	62 (89.9)	7 (10.1)		1*			
		C	11	9 (81.8)	2 (18.2)	1.0586	0.304			
	rs396991	AA	22	21 (95.5)	1 (4.5)		0.305*			
		CA	41	37 (90.2)	4 (9.8)					
		CC	9	7 (77.8)	2 (22.2)					
		A	63	58 (92.1)	5 (7.9)	1.8311	0.176			

		C	50	44 (88)	6 (12)		0.427*			

Ref. Cat., reference category; R, responder; NR, non-responder; OR, odds ratio; CI 95%, 95% confidence Interval 95%; *p-value for Fisher exact test.
