# Supplementary material for: SingleNucleotide Polymorphisms as Biomarkers of Mepolizumab and Benralizumab Treatment Response in Severe Eosinophilic Asthma
Source: Int J Mol Sci. 2024 Jul 26;25(15):8139. doi: 10.3390/ijms25158139 (PMC11311889; doi:10.3390/ijms25158139)
Supplement: Supplementary file 1 [file ijms-25-08139-s001.zip › Table S12.pdf]

Table S12. Association of clinical characteristics of mepolizumab-treated patients with reduction and/or absence of oral corticosteroids.

Characteristics	N	Response		X^2	p-value	Ref. Cat	OR	CI 95%
		R N (%)	NR N (%)					
Sex								
Female	48	34 (70.8)	14 (29.2)	1.125	0.289			
Male	24	14 (58.3)	10 (41.7)					
Age of initiation BT (years)	72	48 (66.7)	24 (33.3)		0.136			
Years with asthma	72	48 (66.7)	24 (33.3)		0.539			
BMI (kg/m2)								
<25	19	15 (78.9)	4 (21.1)	1.7517	0.186			
>25	53	33 (62.3)	20 (37.7)					
Previous respiratory disease								
Yes	34	21 (61.8)	13 (38.2)	0.6966	0.404			
No	38	27 (71.1)	11 (28.9)					
Tobacco consumption								
Non smoker	60	38 (63.3)	22 (36.7)	1.8	0.179			
Current smoker	0	0 (0)	0 (0)					
Former smoker	12	10 (83.3)	2 (16.7)					
Polyps								
Yes	33	21 (63.6)	12 (36.4)	0.2518	0.616			
No	39	27 (69.2)	12 (30.8)					
Allergies								
Yes	37	26 (70.3)	11 (29.7)	0.4448	0.505			
No	35	22 (62.9)	13 (37.1)					
GERD								
Yes	32	20 (62.5)	12 (37.5)	0.45	0.502			
No	40	28 (70)	12 (30)					
SAHS								
Yes	15	11 (73.3)	4 (26.7)	0.379	0.538			
No	57	37 (64.9)	20 (35.1)					
COPD								
Yes	13	10 (76.9)	3 (23.1)	0.7510	0.386			
No	59	38 (64.4)	21 (35.6)					
Age of diagnosis (years)	72	48 (66.7)	24 (33.3)					
<18	2	1 (50)	1 (50)		1*			
>18	70	47 (67.1)	23 (32.9)					
ICS (µg/day)	72	48 (66.7)	24 (33.3)		0.687			
OCS cycles per year								
Yes	57	37 (64.9)	20 (35.1)	0.3790	0.538			
No	15	11 (73.3)	4 (26.7)					
Baseline FEV1 (%)								
<80	51	34 (66.7)	17 (33.3)	0.1488	0.670			
>80	21	13 (61.9)	8 (38.1)					
Exacerbation in previous year								
Yes	47	27 (57.4)	20 (42.6)	5.1779	0.023	Si	3.89	1.24-14.92
No	25	21 (84)	4 (16)					
Basal blood eosinophils (cell/mcl)								
<300	15	11 (73.3)	4 (26.7)	0.5425	0.461			
>300	57	36 (63.2)	21 (36.8)					
Previous BT								
Yes	21	12 (57.1)	9 (42.9)	1.2101	0.271			
No	51	36 (70.6)	15 (29.4)					

BMI, body mass index; GERD, gastroesophageal reflux disease; SAHS, sleep apnea-hypopnea syndrome; COPD, chronic obstructive pulmonary disease; ICS, inhaled corticosteroids; OCS, oral

corticosteroids; FEV1, maximum expiratory volume in the first second of forced expiration; BT, biological therapy.

Ref. Cat, Reference category; NR, Non-Responder; R, Responder; OR, Odds Ratio; CI 95%, Confidence interval; *p-value for Fisher's Exact Test.
