# Supplementary material for: SingleNucleotide Polymorphisms as Biomarkers of Mepolizumab and Benralizumab Treatment Response in Severe Eosinophilic Asthma
Source: Int J Mol Sci. 2024 Jul 26;25(15):8139. doi: 10.3390/ijms25158139 (PMC11311889; doi:10.3390/ijms25158139)
Supplement: Supplementary file 1 [file ijms-25-08139-s001.zip › Table S13.pdf]

Table S13. Association of mepolizumab genetic polymorphisms with corticosteroid reduction and/or absence.

Gene	SNPs	Genotype	N	Response R N (%)	Response NR N (%)	χ²	p-value	Ref Cat	OR	CI 95%
IL1RL1	rs1420101	CC	26	18 (69.2)	8 (30.8)	0.1358	0.934			
		CT	34	22 (64.7)	12 (35.3)					
		TT	12	8 (66.7)	4 (33.3)					
		C	60	40 (66.7)	20 (33.3)	0	1			
		T	46	30 (35.2)	16 (34.8)	0.1204	0.729			
	rs17026974	AA	6	4 (66.7)	2 (33.3)		0.862*			
		AG	28	20 (71.4)	8 (28.6)					
		GG	38	24 (63.2)	14 (36.8)					
		A	34	24 (70.6)	10 (29.4)	0.4458	0.504			
		G	66	44 (66.7)	22 (33.3)		1*			
	rs1921622	AA	20	14 (70)	6 (30)	0.2539	0.881			
		AG	39	26 (66.7)	13 (33.3)					
		GG	13	8 (61.5)	5 (38.5)					
		A	59	40 (67.8)	19 (32.2)	0.1877	0.665			
		G	52	34 (65.4)	18 (34.6)	0.1385	0.71			
IL5	rs4143832	GG	51	34 (66.7)	17 (33.3)		1*			
		GT	17	11 (64.7)	6 (35.3)					
		TT	4	3 (75)	1 (25)					
		G	68	45 (66.2)	23 (33.8)		1*			
		T	21	14 (66.7)	7 (33.3)	0	1			
	rs17690122	AA	51	34 (66.7)	17 (33.3)		1*			
		AG	17	11 (64.7)	6 (35.3)					
		GG	4	3 (75)	1 (25)					
		A	68	45 (66.2)	23 (33.8)		1*			
		G	21	14 (66.7)	7 (33.3)	0	1			
GATA2	rs4857855	CC	53	34 (64.2)	19 (35.8)		0.184*			
		CT	16	13 (81.2)	3 (18.8)					
		TT	3	1 (33.3)	2 (66.7)					
		C	69	47 (68.1)	22 (31.9)		0.256*			
		T	19	14 (73.7)	5 (26.3)	0.572	0.45			
IKZF2	rs12619285	AA	36	24 (66.7)	12 (33.3)		1*			
		AG	31	21 (67.7)	10 (32.3)					
		GG	5	3 (60)	2 (40)					
		A	67	45 (67.2)	22 (32.8)		1*			
		G	36	24 (66.7)	12 (33.3)	0	1			
RAD50	rs11739623	CC	38	27 (71.1)	11 (28.9)		0.581*			
		CT	32	20 (62.5)	12 (37.5)					
		TT	2	1 (50)	1 (50)					
		C	70	47 (67.1)	23 (32.9)		1*			
		T	34	21 (61.8)	13 (38.2)	0.6955	0.404			
	rs4705959	CC	3	1 (33.3)	2 (66.7)		0.214*			
		CT	28	17 (60.7)	11 (39.3)					
		TT	41	30 (73.2)	11 (26.8)					
		C	31	18 (58.1)	13 (41.9)	1.8127	0.178			
		T	69	47 (68.1)	22 (31.9)		0.256*			
FCER1A	rs2251746	CC	5	3 (60)	2 (40)	0.7759	0.678			
		CT	26	19 (73.1)	7 (26.9)					
		TT	41	26 (63.4)	15 (36.6)					
		C	31	22 (71)	9 (29)	0.4532	0.501			
		T	67	45 (67.2)	22 (32.8)		1*			
	rs2427837	AA	41	25 (61)	16 (39)		0.436*			
		AG	25	19 (76)	6 (24)					
		GG	6	4 (66.7)	2 (33.3)					
		A	31	23 (74.2)	8 (25.8)	1.3879	0.239			
		G	66	44 (66.7)	22 (33.3)		1*			
FCER1B	rs1441586	CC	11	7 (63.6)	4 (36.4)	0.1577	0.924			
		CT	41	27 (65.9)	14 (34.1)					
		TT	20	14 (70)	6 (30)					
		C	52	34 (65.4)	18 (34.6)	0.1385	0.71			
		T	61	41 (67.2)	20 (32.8)	0.0537	0.817			

Gene	SNPs	Genotype	N	Response R N (%)	Response NR N (%)	χ²	p-value	Ref Cat	OR	CI 95%
FCER1B	rs573790	CC	30	20 (66.7)	10 (33.3)		1*			
		CT	36	24 (66.7)	12 (33.3)					
		TT	6	4 (66.7)	2 (33.3)					
		C	66	44 (66.7)	22 (33.3)		1*			
		T	42	28 (66.7)	14 (33.3)	0	1*			
	rs569108	AA	63	44 (69.8)	19 (30.2)	2.2857	0.131			
		AG	9	4 (44.4)	5 (55.6)					
		GG	0	0 (0)	0 (0)					
		A	-	-	-					
		G	9	4 (44.4)	5 (55.6)	2.2857	0.131			
ZNF415	rs1054485	GG	17	10 (58.8)	7 (41.2)	0.7287	0.695			
		GT	31	22 (71)	9 (29)					
		TT	24	16 (66.7)	8 (33.3)					
		G	48	32 (66.7)	16 (33.3)	0	1			
		T	55	38 (69.1)	17 (30.9)	0.6160	0.433			
FCGR2A	rs1801274	AA	27	19 (70.4)	8 (29.6)		0.86*			
		AG	25	16 (64)	9 (36)					
		GG	20	13 (65)	7 (35)					
		A	52	35 (67.3)	17 (32.7)		1*			
		G	45	29 (64.4)	16 (35.6)	0.2667	0.606			
FCGR2B	rs3219018	CC	1	1 (100)	0 (0)		0.861*			
		CG	24	15 (62.5)	9 (37.5)					
		GG	47	32 (68.1)	15 (31.9)					
		C	25	16 (64)	9 (36)	0.1226	0.726			
		G	71	47 (66.2)	24 (33.8)		1*			
	rs1050501	CC	1	1 (100)	0 (0)		0.216*			
		CT	16	8 (50)	8 (50)					
		TT	55	39 (70.9)	16 (29.1)					
		C	17	9 (52.9)	8 (47.1)	1.8866	0.17			
		T	71	47 (66.2)	24 (33.8)		1*			
FCGR3A	rs10127939	AA	61	42 (68.9)	19 (31.1)		0.11*			
		AC	8	3 (37.5)	5 (62.5)					
		CC	3	3 (100)	0 (0)					
		A	69	45 (65.2)	24 (34.8)		0.546*			
		C	11	6 (54.5)	5 (45.5)	0.8584	0.354			
	rs396991	AA	22	15 (68.2)	7 (31.8)	0.0349	0.983			
		CA	41	27 (65.9)	14 (34.1)					
		CC	9	6 (66.7)	3 (33.3)					
		A	63	42 (66.7)	21 (33.3)	0	1			
		C	50	33 (66)	17 (34)	0.0327	0.856			

Ref. Cat., reference category; R, responder; NR, non-responder; OR, odds ratio; CI 95%, 95% confidence Interval 95%; *p-value for Fisher exact test.
