# Supplementary material for: SingleNucleotide Polymorphisms as Biomarkers of Mepolizumab and Benralizumab Treatment Response in Severe Eosinophilic Asthma
Source: Int J Mol Sci. 2024 Jul 26;25(15):8139. doi: 10.3390/ijms25158139 (PMC11311889; doi:10.3390/ijms25158139)
Supplement: Supplementary file 1 [file ijms-25-08139-s001.zip › Table S14.pdf]

Table S14. Association of clinical characteristics of mepolizumab-treated patients with improvement in lung function.

Characteristics	N	Response R N (%)	NR N (%)	X^2	p-value	Ref. Cat	OR	CI 95%
Sex								
Female	48	33 (68.8)	15 (31.2)	1.7517	0.186			
Male	24	20 (83.3)	4 (16.7)					
Age of initiation BT (years)	72	53 (73.6)	19 (26.4)		0.023		1.05	1.01-1.11
Years with asthma	72	53 (73.6)	19 (26.4)		0.066			
BMI (kg/m2)								
<25	19	14 (73.7)	5 (26.3)	0.000	0.993			
>25	53	39 (73.6)	14 (26.4)					
Previous respiratory disease								
Yes	34	27 (79.4)	7 (20.6)	1.1159	0.291			
No	38	26 (68.4)	12 (31.6)					
Tobacco consumption								
Non smoker	60	44 (73.3)	16 (26.7)	0.0143	0.905			
Current smoker	0	0 (0)	0 (0)					
Former smoker	12	9 (75)	3 (25)					
Polyps								
Yes	33	23 (69.7)	10 (30.3)	0.4805	0.488			
No	39	30 (76.9)	9 (23.1)					
Allergies								
Yes	37	25 (67.6)	12 (32.4)	1.4311	0.232			
No	35	28 (80)	7 (20)					
GERD								
Yes	32	23 (71.9)	9 (28.1)	0.0893	0.765			
No	40	30 (75)	10 (25)					
SAHS								
Yes	15	12 (80)	3 (20)	0.3981	0.528			
No	57	41 (71.9)	16 (28.1)					
COPD								
Yes	13	11 (84.6)	2 (15.4)	0.989	0.320			
No	59	42 (7.12)	17 (28.8)					
Age of diagnosis (years)	72	53 (73.6)	19 (26.4)		0.066			
<18	2	0 (0)	2 (100)		0.067*			
>18	70	53 (75.7)	17 (24.3)					
ICS (µg/day)	72	53 (73.6)	19 (26.4)		0.134			
OCS cycles per year								
Yes	57	41 (71.9)	16 (28.1)	0.3981	0.528			
No	15	12 (80)	3 (20)					
Baseline FEV1 (%)								
<80	51	38 (74.5)	13 (25.5)	0.0727	0.787			
>80	21	15 (71.4)	6 (28.6)					
Exacerbation in previous year								
Yes	47	33 (70.2)	14 (29.8)	0.8048	0.370			
No	25	20 (80)	5 (20)					
Basal blood eosinophils (cell/mcl)								
<300	15	11 (73.3)	4 (26.7)	0.000	0.987			
>300	57	42 (73.7)	15 (26.3)					
Previous BT								
Yes	21	15 (71.4)	6 (28.6)	0.0727	0.787			
No	51	38 (74.5)	13 (25.5)					

BMI, body mass index; GERD, gastroesophageal reflux disease; SAHS, sleep apnea-hypopnea syndrome; COPD, chronic obstructive pulmonary disease; ICS, inhaled corticosteroids; OCS, oral corticosteroids; FEV1, maximum expiratory volume in the first second of forced expiration; BT, biological therapy.
Ref. Cat, Reference category; NR, Non-Responder; R, Responder; OR, Odds Ratio; CI 95%, Confidence interval; *p-value for Fisher's Exact Test.
