# Supplementary material for: SingleNucleotide Polymorphisms as Biomarkers of Mepolizumab and Benralizumab Treatment Response in Severe Eosinophilic Asthma
Source: Int J Mol Sci. 2024 Jul 26;25(15):8139. doi: 10.3390/ijms25158139 (PMC11311889; doi:10.3390/ijms25158139)
Supplement: Supplementary file 1 [file ijms-25-08139-s001.zip › Table S15.pdf]

Table S15. Association of mepolizumab genetic polymorphisms with improved lung function (FEV1).

Gene	SNPs	Genotype	N	Response R N (%)	Response NR N (%)	χ²	p-value	Ref Cat	OR	CI 95%
		CC	26	19 (73.1)	7 (26.9)				1	
		CT	34	22 (64.7)	12 (35.3)	5.6938	0.058	CC	0.68	0.21-2.03
	rs1420101	TT	12	12 (100)	0 (0)				$4.3\ e^{7}$	$9.8\ x10^{-43}$-NA
		C	60	41 (68.3)	19 (31.7)	5.1623	0.023	C	$5.4e^{7}$	$5.4\ x10^{-51}$-NA
		T	46	34 (73.9)	12 (26.1)	0.006	0.938			
		AA	6	6 (100)	0 (0)					
		AG	28	19 (67.9)	9 (32.1)		0.269*			
IL1RL1	rs17026974	GG	38	28 (73.7)	10 (26.3)					
		A	34	25 (73.5)	9 (26.5)	0.0002	0.988			
		G	66	47 (71.2)	19 (28.8)		0.126*			
		AA	20	18 (90)	2 (10)					
		AG	39	27 (69.2)	12 (30.8)	4.1261	0.127			
	rs1921622	GG	13	8 (61.5)	5 (38.5)					
		A	59	45 (76.3)	14 (23.7)	1.1903	0.275			
		G	52	35 (67.3)	17 (32.7)	3.8291	0.050	G	4.37	1.08-29.51
		GG	51	38 (74.5)	13 (25.5)					
		GT	17	12 (70.6))	5 (29.4)		0.949*			
	rs4143832	TT	4	3 (75)	1 (25)					
		G	68	50 (73.5)	18 (26.5)		1*			
		T	21	15 (71.4)	6 (28.6)	0.0727	0.787			
IL5		AA	51	38 (74.5)	13 (25.5)					
		AG	17	12 (70.6)	5 (29.4)		0.949*			
	rs17690122	GG	4	3 (75)	1 (25)					
		A	68	50 (73.5)	18 (26.5)		1*			
		G	21	15 (71.4)	6 (28.6)	0.0727	0.787			
		CC	53	39 (73.6)	14 (26.4)					
		CT	16	12 (75)	4 (25)		1*			
GATA2	rs4857855	TT	3	2 (66.7)	1 (33.3)					
		C	69	51 (73.9)	18 (26.1)		1*			
		T	19	14 (73.7)	5 (26.3)	0.0001	0.993			
		AA	36	29 (80.6)	7 (19.4)					
		AG	31	20 (64.5)	11 (35.5)		0.311*			
IKZF2	rs12619285	GG	5	4 (80)	1 (20)					
		A	67	49 (73.1)	18 (26.9)		1*			
		G	36	24 (66.7)	12 (33.3)	1.7875	0.181			
		CC	38	27 (71.1)	11 (28.9)					
		CT	32	24 (75)	8 (25)		0.887*			
	rs11739623	TT	2	2 (100)	0 (0)					
		C	70	51 (72.9)	19 (27.1)		1*			
		T	34	26 (76.5)	8 (23.5)	0.2712	0.603			
RAD50		CC	3	3 (100)	0 (0)					
		CT	28	20 (71.4)	8 (28.6)		0.816*			
	rs4705959	TT	41	30 (73.2)	11 (26.8)					
		C	31	23 (74.2)	8 (25.8)	0.0095	0922			
		T	69	50 (72.5)	19 (27.5)		0.561*			
		CC	5	5 (100)	0 (0)					
		CT	26	21 (80.8)	5 (19.2)		0.154*			
	rs2251746	TT	41	27 (65.9)	14 (34.1)					
		C	31	26 (83.9)	5 (16.1)	2.95	0.0859			
		T	67	48 (71.6)	19 (28.4)		0.316*			
FCER1A		AA	6	6 (100)	0 (0)				$2.5e^{7}$	$2.5\ x10^{-60}$-NA
		AG	25	21 (84)	4 (16)		0.057*	GG	3.03	0.93-11.87
	rs2427837	GG	41	26 (63.4)	15 (36.6)				1	
		A	31	27 (87.1)	4 (12.9)	5.0967	0.024	GG	3.89	1.23-15.07
		G	66	47 (71.2)	19 (28.8)		0.331*	G	$1.7e^{7}$	$1.4\ x10^{-60}$-NA
		CC	11	6 (54.5)	5 (45.5)					
		CT	41	29 (70.7)	12 (29.3)	4.9988	0.082			
FCER1B	rs1441586	TT	20	18 (90)	2 (10)					
		C	52	35 (67.3)	17 (32.7)	3.291	0.050	C	4.37	1.07-29.51
		T	61	47 (77)	14 (23)	2.4296	0.119			

Gene	SNPs	Genotype	N	Response		χ²	p-value	Ref Cat	OR	CI 95%
				R N (%)	NR N (%)					
FCER1B	rs573790	CC	30	20 (66.7)	10 (33.3)		0.602*			
		CT	36	28 (77.8)	8 (22.5)					
		TT	6	5 (83.3)	1 (16.7)					
		C	66	48 (72.7)	18 (27.3)		1*			
		T	42	33 (78.6)	9 (21.4)	1.2768	0.259			
	rs569108	AA	63	51 (81)	12 (19)	13.983	<0.001*	AG	14.89	3.14-108.86
		AG	9	2 (22.2)	7 (77.8)					
		GG	0	0 (0)	0 (0)					
		A	-	-	-					
		G	9	2 (22.2)	7 (77.8)	13.983	<0.001*	G	14.89	3.14-108.86
ZNF41	rs1054485	GG	17	14 (82.4)	3 (17.6)	0.8756	0.645			
		GT	31	22 (71)	9 (29)					
		TT	24	17 (70.8)	7 (29.2)					
		G	48	36 (75)	12 (25)	0.143	0.705			
		T	55	39 (70.9)	16 (29.1)	0.8755	0.349			
FCGR2A	rs1801274	AA	27	18 (66.7)	9 (33.3)	1.2155	0.545			
		AG	25	20 (80)	5 (20)					
		GG	20	15 (75)	5 (25)					
		A	52	38 (73.1)	14 (26.9)	0.0275	0.868			
		G	45	35 (77.8)	10 (22.2)	1.0725	0.3			
FCGR2B	rs3219018	CC	1	0 (0)	1 (100)		0.217*			
		CG	24	19 (79.2)	5 (20.8)					
		GG	47	34 (72.3)	13 (27.7)					
		C	25	19 (76)	6 (2)	0.1125	0.737			
		G	71	53 (74.6)	18 (25.4)		0.264*			
	rs1050501	CC	0	1 (100)	0 (0)		1*			
		CT	16	12 (75)	4 (25)					
		TT	55	40 (72.7)	15 (27.3)					
		C	17	13 (76.5)	4 (23.5)	0.037	0.76			
		T	71	52 (73.2)	19 (26.8)		1*			
FCGR3A	rs10127939	AA	61	46 (75.4)	15 (24.6)		0.618*			
		AC	8	5 (62.5)	3 (37.5)					
		CC	3	2 (66.7)	1 (33.3)					
		A	69	51 (73.9)	18 (26.1)		1*			
		C	11	7 (63.6)	4 (36.4)	0.665	0.415			
	rs396991	AA	22	16 (72.7)	6 (27.3)	1.2653	0.531			
		CA	41	29 (70.7)	12 (29.3)					
		CC	9	8 (88.9)	1 (11.1)					
		A	63	45 (71.4)	18 (28.6)	1.2359	0.266			
		C	50	37 (74)	13 (26)	0.0127	0.91			

Ref. Cat., reference category; R, responder; NR, non-responder; OR, odds ratio; CI 95%, 95% confidence Interval 95%; *p-value for Fisher exact test.
