# Supplementary material for: SingleNucleotide Polymorphisms as Biomarkers of Mepolizumab and Benralizumab Treatment Response in Severe Eosinophilic Asthma
Source: Int J Mol Sci. 2024 Jul 26;25(15):8139. doi: 10.3390/ijms25158139 (PMC11311889; doi:10.3390/ijms25158139)
Supplement: Supplementary file 1 [file ijms-25-08139-s001.zip › Table S16.pdf]

Table S16. Association of clinical characteristics of mepolizumab-treated patients with response to at least one parameter.

Characteristics	N	Response R N (%)	Response NR N (%)	X^2	p-value	Ref. Cat	OR	CI 95%
Sex								
Female	48	46 (95.8)	2 (4.2)		0.549*			
Male	24	24 (100)	0 (0)					
Age of initiation BT (years)	72	70 (97.2)	2 (2.8)		0.175			
Years with asthma	72	70 (97.2)	2 (2.8)		0.411			
BMI (kg/m2)								
<25	19	19 (100)	0 (0)		1*			
>25	53	51 (96.2)	2 (3.8)					
Previous respiratory disease								
Yes	34	32 (94.1)	2 (5.9)		0.22*			
No	38	38 (100)	0 (0)					
Tobacco consumption								
Non-smoker	60	58 (96.7)	2 (3.3)		1*			
Current smoker	0	0 (0)	0 (0)					
Former smoker	12	12 (100)	0 (0)					
Polyps								
Yes	33	32 (97)	1 (3)		1*			
No	39	38 (97.4)	1 (2.6)					
Allergies								
Yes	37	36 (97.3)	1 (2.7)		1*			
No	35	34 (97.1)	1 (2.9)					
GERD								
Yes	32	31 (96.9)	1 (3.1)		1*			
No	40	39 (97.5)	1 (2.5)					
SAHS								
Yes	15	15 (100)	0 (0)		1*			
No	57	55 (96.5)	2 (3.5)					
COPD								
Yes	13	13 (100)	0 (0)		1*			
No	59	57 (96.6)	2 (3.4)					
Age of diagnosis (years)								
<18	2	2 (100)	0 (0)		1*			
>18	70	68 (97.1)	2 (2.9)					
ICS (µg/day)	72	70 (97.2)	2 (2.8)		0.538			
OCS cycles per year								
Yes	57	56 (98.2)	1 (1.8)		0.376*			
No	15	14 (93.3)	1 (6.7)					
Baseline FEV1 (%)								
<80	51	50 (98)	1 (2)		0.501*			
>80	21	20 (95.2)	1 (4.8)					
Exacerbation in previous year								
Yes	47	45 (95.7)	2 (4.3)		0.540*			
No	25	25 (100)	0 (0)					
Basal blood eosinophils (cell/mcl)								
<300	15	14 (93.3)	1 (6.7)		0.378*			
>300	57	56 (98.2)	1 (1.8)					
Previous BT								
Yes	21	20 (95.2)	1 (4.8)		0.511*			
No	51	50 (98)	1 (20)					

BMI, body mass index; GERD, gastroesophageal reflux disease; SAHS, sleep apnea-hypopnea syndrome; COPD, chronic obstructive pulmonary disease; ICS, inhaled corticosteroids; OCS, oral corticosteroids; FEV1, maximum expiratory volume in the first second of forced expiration; BT, biological therapy.
Ref. Cat, Reference category; NR, Non-Responder; R, Responder; OR, Odds Ratio; CI 95%, Confidence interval; *p-value for Fisher's Exact Test.
