# Supplementary material for: SingleNucleotide Polymorphisms as Biomarkers of Mepolizumab and Benralizumab Treatment Response in Severe Eosinophilic Asthma
Source: Int J Mol Sci. 2024 Jul 26;25(15):8139. doi: 10.3390/ijms25158139 (PMC11311889; doi:10.3390/ijms25158139)
Supplement: Supplementary file 1 [file ijms-25-08139-s001.zip › Table S17.pdf]

Table S17. Association of mepolizumab genetic polymorphisms with responders to at least one criterion.

Gene	SNPs	Genotype	N	Response R N (%)	Response NR N (%)	χ²	p-value	Ref Cat	OR	CI 95%
IL1RL1	rs1420101	CC	26	25 (96.2)	1 (3.8)		1*			
		CT	34	33 (97.1)	1 (2.9)					
		TT	12	12 (100)	0 (0)					
		C	60	58 (96.7)	2 (3.3)		1*			
		T	46	45 (97.8)	1 (2.2)		1*			
	rs17026974	AA	6	6 (100)	0 (0)		0.584*			
		AG	28	28 (100)	0 (0)					
		GG	38	36 (84.7)	2 (5.3)					
		A	34	34 (100)	0 (0)		0.495*			
		G	66	64 (97)	2 (3)		1*			
	rs1921622	AA	20	20 (100)	0 (0)		0.695*			
		AG	39	37 (94.9)	2 (5.1)					
		GG	13	13 (100)	0 (0)					
		A	59	57 (96.6)	2 (3.4)		1*			
		G	52	50 (96.2)	2 (3.8)		1*			
IL5	rs4143832	GG	51	50 (98)	1 (2)		0.501*			
		GT	17	16 (94.1)	1 (5.9)					
		TT	4	4 (100)	0 (0)					
		G	68	66 (97.1)	2 (2.9)		1*			
		T	21	20 (95.2)	1 (4.8)		0.501*			
	rs17690122	AA	4	4 (100)	0 (0)		0.501*			
		AG	17	16 (94.1)	1 (5.9)					
		GG	51	50 (98)	1 (2)					
		A	68	66 (97.1)	2 (2.9)		1*			
		G	21	20 (95.2)	1 (4.8)		0.501*			
GATA2	rs4857855	CC	53	52 (98.1)	1 (1.9)		0.129			
		CT	16	16 (100)	0 (0)					
		TT	3	2 (66.7)	1 (33.3)					
		C	69	68 (98.6)	1 (1.4)		0.026	TT	34	1.1-1136.7
		T	19	18 (94.7)	1 (5.3)		0.461			
IKZF2	rs12619285	AA	36	35 (97.2)	1 (2.8)		1*			
		AG	31	30 (96.8)	1 (3.2)					
		GG	5	5 (100)	0 (0)					
		A	67	65 (97)	2 (3)		1*			
		G	36	35 (97.2)	1 (2.8)		1*			
RAD50	rs11739623	CC	38	36 (94.7)	2 (5.3)		0.524*			
		CT	32	32 (100)	0 (0)					
		TT	2	2 (100)	0 (0)					
		C	70	68 (97.1)	2 (2.9)		1*			
		T	34	34 (100)	0 (0)		0.495*			
	rs4705959	CC	3	3 (100)	0 (0)		0.551*			
		CT	28	28 (100)	0 (0)					
		TT	41	39 (95.1)	2 (4.9)					
		C	31	31 (100)	0 (0)		0.503*			
		T	69	67 (97.1)	2 (2.9)		0.795*			
FCER1A	rs2251746	CC	41	40 (97.6)	1 (2.4)		1*			
		CT	26	25 (96.2)	1 (3.8)					
		TT	5	5 (100)	0 (0)					
		C	31	30 (96.8)	1 (3.2)		1*			
		T	67	65 (97)	2 (3)		1*			
	rs2427837	AA	6	6 (100)	0 (0)		0.599*			
		AG	25	25 (100)	0 (0)					
		GG	41	39 (95.1)	2 (4.9)					
		A	31	31 (100)	0 (0)		0.503*			
		G	66	64 (97)	2 (3)		1*			
FCER1B	rs1441586	CC	11	10 (90.9)	1 (9.1)		0.358*			
		CT	41	40 (97.6)	1 (2.4)					
		TT	20	20 (100)	0 (0)					
		C	52	50 (96.2)	2 (3.8)		1*			
		T	61	60 (98.4)	1 (1.6)		0.284*			

Gene	SNPs	Genotype	N	Response		χ²	p-value	Ref Cat	OR	CI 95%
				R N (%)	NR N (%)					
FCER1B	rs573790	CC	30	29 (96.7)	1 (3.3)					
		CT	36	35 (97.2)	1 (2.8)		1*			
		TT	6	6 (100)	0 (0)					
		C	66	64 (97)	2 (3)		1*			
		T	42	41 (97.6)	1 (2.4)		1*			
	rs569108	AA	63	62 (98.4)	1 (1.6)					
		AG	9	8 (88.9)	1 (11.1)		0.236*			
		GG	0	0 (0)	0 (0)					
		A	-	-	-					
		G	9	8 (88.9)	1 (11.1)		0.236*			
ZNF415	rs1054485	GG	17	16 (94.1)	1 (5.9)		0.321*			
		GT	31	31 (100)	0 (0)					
		TT	24	23 (95.8)	1 (4.2)					
		G	48	47 (97.9)	1 (2.1)		1*			
		T	55	54 (98.2)	1 (1.8)		0.419*			
FCGR2A	rs1801274	AA	27	27 (100)	0 (0)					
		AG	25	24 (96)	1 (4)		0.525*			
		GG	20	19 (95)	1 (5)					
		A	52	51 (98.1)	1 (1.9)		0.481*			
		G	45	43 (95.6)	2 (4.4)		0.525*			
FCGR2B	rs3219018	CC	1	1 (100)	0 (0)					
		CG	24	23 (95.8)	1 (4.2)		1*			
		GG	47	46 (97.9)	1 (2.1)					
		C	25	24 (96)	1 (4)		1*			
		G	71	69 (97.2)	2 (2.8)		1*			
	rs1050501	CC	1	1 (100)	0 (0)					
		CT	16	16 (100)	0 (0)		1*			
		TT	55	53 (96.4)	2 (3.6)					
		C	17	17 (100)	0 (0)		1*			
		T	71	69 (97.2)	2 (2.8)		1*			
FCGR3A	rs10127939	AA	61	59 (96.7)	2 (3.3)					
		AC	8	8 (100)	0 (0)		1*			
		CC	3	3 (100)	0 (0)					
		A	69	67 (97.1)	2 (2.9)		1*			
		C	11	11 (100)	0 (0)		1*			
	rs396991	AA	22	21 (95.5)	1 (4.5)					
		CA	41	40 (97.6)	1 (2.4)		1*			
		CC	9	9 (100)	0 (0)					
		A	63	61 (96.8)	2 (3.2)		1*			
		C	50	49 (98)	1 (2)		0.521*			

Ref. Cat., reference category; R, responder; NR, non-responder; OR, odds ratio; CI 95%, 95% confidence Interval 95%; *p-value for Fisher exact test.
