# Supplementary material for: SingleNucleotide Polymorphisms as Biomarkers of Mepolizumab and Benralizumab Treatment Response in Severe Eosinophilic Asthma
Source: Int J Mol Sci. 2024 Jul 26;25(15):8139. doi: 10.3390/ijms25158139 (PMC11311889; doi:10.3390/ijms25158139)
Supplement: Supplementary file 1 [file ijms-25-08139-s001.zip › Table S18.pdf]

Table S18. Association of clinical characteristics of patients treated with mepolizumab with response on at least 2 criteria.

Characteristics	N	Response R N (%)	Response NR N (%)	X^2	p-value	Ref. Cat	OR	CI 95%
Sex								
Female	48	35 (72.9)	13 (27.1)	1.9688	0.161			
Male	24	21 (87.5)	3 (12.5)					
Age of initiation BT (years)	72	56 (77.8)	16 (22.2)		0.267			
Years with asthma	72	56 (77.8)	16 (22.2)		0.634			
BMI (kg/m2)								
<25	19	15 (78.9)	4 (21.1)	0.0243	0.886			
>25	53	41 (77.4)	12 (22.6)					
Previous respiratory disease								
Yes	34	26 (76.5)	8 (23.5)	0.0637	0.801			
No	38	30 (78.9)	8 (21.1)					
Tobacco consumption								
Non-smoker	60	46 (76.7)	14 (23.3)	0.0257	0.612			
Current smoker	0	0 (0)	0 (0)					
Former smoker	12	10 (83.3)	2 (16.7)					
Polyps								
Yes	33	25 (75.8)	8 (24.2)	0.1437	0.705			
No	39	31 (79.5)	8 (20.5)					
Allergies								
Yes	37	29 (78.4)	8 (21.6)	0.0159	0.9			
No	35	27 (77.1)	8 (22.9)					
GERD								
Yes	32	29 (90.6)	3 (9.4)		1*			
No	40	36 (90)	4 (10)					
SAHS								
Yes	15	13 (86.7)	2 (13.3)	0.8662	0.352			
No	57	43 (75.4)	14 (24.6)					
COPD								
Yes	13	10 (76.9)	3 (23.1)	0.0067	0.935			
No	59	46 (78)	13 (22)					
Age of diagnosis (years)								
<18	2	1 (50)	1 (50)		0.398			
>18	70	55 (78.6)	15 (21.4)					
ICS (μg/day)	72	56 (77.8)	16 (22.2)		0.226			
OCS cycles per year								
Yes	57	44 (77.2)	13 (22.8)	0.0541	0.816			
No	15	12 (80)	3 (20)					
Baseline FEV1 (%)								
<80	51	41 (80.4)	10 (19.6)	0.1592	0.690			
>80	21	16 (76.2)	5 (23.8)					
Exacerbation in previous year								
Yes	47	33 (70.2)	14 (29.8)	4.4819	0.034	Si	4.88	1.21-32.99
No	25	23 (92)	2 (8)					
Basal blood eosinophils (cell/mcl)								
<300	15	11 (73.3)	4 (26.7)	0.3901	0.532			
>300	57	46 (80.7)	11 (19.3)					

BMI, body mass index; GERD, gastroesophageal reflux disease; SAHS, sleep apnea-hypopnea syndrome; COPD, chronic obstructive pulmonary disease; ICS, inhaled corticosteroids; OCS, oral corticosteroids; FEV1, maximum expiratory volume in the first second of forced expiration; BT, biological therapy.

Ref. Cat, Reference category; NR, Non-Responder; R, Responder; OR, Odds Ratio; CI 95%, Confidence interval; *p-value for Fisher's Exact Test.
