# Supplementary material for: SingleNucleotide Polymorphisms as Biomarkers of Mepolizumab and Benralizumab Treatment Response in Severe Eosinophilic Asthma
Source: Int J Mol Sci. 2024 Jul 26;25(15):8139. doi: 10.3390/ijms25158139 (PMC11311889; doi:10.3390/ijms25158139)
Supplement: Supplementary file 1 [file ijms-25-08139-s001.zip › Table S19.pdf]

Table S19. Association of mepolizumab genetic polymorphisms with 2-criteria response.

Gene	SNPs	Genotype	N	Response R N (%)	Response NR N (%)	χ²	p-value	Ref Cat	OR	CI 95%
IL1RL1	rs1420101	CC	26	21 (80.8)	5 (19.2)	0.2218	0.895			
		CT	34	26 (76.5)	8 (23.5)					
		TT	12	9 (75)	3 (25)					
		C	60	47 (78.3)	13 (21.7)	0.0643	0.8			
		T	46	35 (76.1)	11 (23.9)	0.2107	0.646			
	rs17026974	AA	6	5 (83.3)	1 (16.7)		1*			
		AG	28	22 (78.6)	6 (21.4)					
		GG	38	29 (76.3)	9 (23.7)					
		A	34	27 (79.4)	7 (20.6)	0.0995	0.752			
		G	66	51 (77.3)	15 (22.7)		1*			
	rs1921622	AA	20	15 (75)	5 (25)		0.929*			
		AG	39	31 (79.5)	8 (20.5)					
		GG	13	10 (76.9)	3 (23.1)					
		A	59	46 (78)	13 (22)	0.0067	0.935			
		G	52	41 (78.8)	11 (21.2)	0.1236	0.725			
IL5	rs4143832	GG	51	40 (78.4)	11 (21.6)		1*			
		GT	17	13 (76.5)	4 (23.5)					
		TT	4	3 (75)	1 (25)					
		G	68	53 (77.9)	15 (22.1)		1*			
		T	21	16 (76.2)	5 (23.8)	0.0432	0.835			
	rs17690122	AA	51	40 (78.4)	11 (21.6)		1*			
		AG	17	13 (76.5)	4 (23.5)					
		GG	4	3 (75)	1 (25)					
		A	68	53 (77.9)	15 (22.1)		1*			
		G	21	16 (76.2)	5 (23.8)	0.0432	0.835			
GATA2	rs4857855	CC	53	39 (73.6)	14 (26.4)		0.202*			
		CT	16	15 (93.8)	1 (6.2)					
		TT	3	2 (66.7)	1 (33.3)					
		C	69	54 (78.3)	15 (21.7)		0.535*			
		T	19	17 (89.5)	2 (10.5)	2.0428	0.153			
IKZF2	rs12619285	AA	36	29 (80.6)	7 (19.4)		0.819*			
		AG	31	23 (74.2)	8 (25.8)					
		GG	5	4 (80)	1 (20)					
		A	67	52 (77.6)	15 (22.4)		1*			
		G	36	27 (75)	9 (25)		0.778*			
RAD50	rs11739623	CC	38	29 (76.3)	9 (23.7)		1*			
		CT	32	25 (78.1)	7 (21.9)					
		TT	2	2 (100)	0 (0)					
		C	70	54 (77.1)	16 (22.9)		1*			
		T	34	27 (79.4)	7 (20.6)	0.0995	0.752			
	rs4705959	CC	3	3 (100)	0 (0)		0.897*			
		CT	28	21 (75)	7 (25)					
		TT	41	32 (78)	9 (22)					
		C	31	24 (77.4)	7 (22.6)	0.0040	0.949			
		T	69	53 (76.8)	16 (23.2)		1*			
FCER1A	rs2251746	CC	5	4 (80)	1 (20)		0.216*			
		CT	26	23 (88.5)	3 (11.5)					
		TT	41	29 (70.7)	12 (29.3)					
		C	31	27 (87.1)	4 (12.9)	2.7353	0.076			
		T	67	52 (77.6)	15 (22.4)		1*			
	rs2427837	AA	6	5 (83.3)	1 (16.7)		0.100*			
		AG	25	23 (92)	2 (8)					
		GG	41	29 (70.7)	12 (29.3)					
		A	31	28 (90.3)	3 (9.7)	4.1079	0.042	GG	4.33	1.23-20.42
		G	66	51 (77.3)	15 (22.7)		1*			
FCER1B	rs1441586	CC	11	7 (63.6)	4 (36.4)		0.383*			
		CT	41	32 (78)	9 (22)					
		TT	20	17 (85)	3 (15)					
		C	52	39 (75)	13 (25)	0.8357	0.361			
		T	61	49 (80.3)	12 (19.7)	1.502	0.22			

Gene	SNPs	Genotype	N	Response R N (%)	Response NR N (%)	χ²	p-value	Ref Cat	OR	CI 95%
FCER1B	rs573790	CC	30	25 (83.3)	5 (16.7)		0.577*			
		CT	36	27 (75)	9 (25)					
		TT	6	4 (66.7)	2 (33.3)					
		C	66	52 (78.8)	14 (21.2)		0.609*			
		T	42	31 (73.8)	11 (26.2)	0.9184	0.338			
	rs569108	AA	63	53 (82.5)	10 (17.5)	7.5188	0.006	AG	6.63	1.51-31.22
		AG	9	4 (44.4)	5 (55.6)					
		GG	0	0 (0)	0 (0)					
		A	-	-	-					
		G	9	4 (44.4)	5 (55.6)	7.5188	0.006	G	6.63	1.51-31.22
ZNF415	rs1054485	GG	17	11 (64.7)	6 (35.3)	2.2567	0.324			
		GT	31	25 (80.6)	6 (19.4)					
		TT	24	20 (83.3)	4 (16.7)					
		G	48	36 (75)	12 (25)	0.6429	0.423			
		T	55	45 (81.8)	10 (18.2)	2.2002	0.138			
FCGR2A	rs1801274	AA	27	21 (77.8)	6 (22.2)	0.1607	0.923			
		AG	25	20 (80)	5 (20)					
		GG	20	15 (75)	5 (25)					
		A	52	41 (78.8)	11 (21.2)	0.1236	0.725			
		G	45	35 (77.8)	10 (22.2)	0	1*			
FCGR2B	rs3219018	CC	1	1 (100)	0 (0)		0.516*			
		CG	24	17 (70.8)	7 (29.2)					
		GG	47	38 (80.9)	9 (19.1)					
		C	25	18 (72)	7 (28)	0.7397	0.389			
		G	71	55 (77.5)	16 (22.5)		1*			
	rs1050501	CC	1	1 (100)	0 (0)		0.803*			
		CT	16	12 (75)	4 (25)					
		TT	55	43 (78.2)	12 (21.8)					
		C	17	13 (76.5)	4 (23.5)	0.022	0.882			
		T	71	55 (77.5)	16 (22.5)		1*			
FCGR3A	rs10127939	AA	61	49 (80.3)	12 (19.7)		0.11*			
		AC	8	4 (50)	4 (50)					
		CC	3	3 (100)	0 (0)					
		A	69	53 (76.8)	16 (23.2)		1*			
		C	11	7 (63.6)	4 (36.4)	1.5022	0.22			
	rs396991	AA	22	19 (86.4)	3 (13.6)		0.431*			
		CA	41	31 (75.6)	10 (24.4)					
		CC	9	6 (66.7)	3 (33.3)					
		A	63	50 (79.4)	13 (20.6)	0.7347	0.391			
		C	50	37 (74)	13 (26)	1.3512	0.245			

Ref. Cat., reference category; R, responder; NR, non-responder; OR, odds ratio; CI 95%, 95% confidence Interval 95%; *p-value for Fisher exact test.
