# Supplementary material for: SingleNucleotide Polymorphisms as Biomarkers of Mepolizumab and Benralizumab Treatment Response in Severe Eosinophilic Asthma
Source: Int J Mol Sci. 2024 Jul 26;25(15):8139. doi: 10.3390/ijms25158139 (PMC11311889; doi:10.3390/ijms25158139)
Supplement: Supplementary file 1 [file ijms-25-08139-s001.zip › Table S2.pdf]

Table S2. Linkage disequilibrium of the studied SNPs.

CHR	BP	SNP	CHR	BP	SNP	R2	D'
1	159288755	rs2427837	1	159302270	rs2251746	0.900715	0.962124
1	161544752	rs396991	1	161548543	rs10127939	0.223882	1
1	161548543	rs10127939	1	161662856	rs3219018	0.307539	0.714679
2	102335900	rs17026974	2	102341256	rs1420101	0.493889	1
2	102335900	rs17026974	2	102349607	rs1921622	0.237267	1
2	102341256	rs1420101	2	102349607	rs1921622	0.363612	0.811931
5	132527285	rs4143832	5	132532143	rs17690122	1	1
5	132528460	rs11739623	5	132530099	rs4705959	0.837165	0.959821
11	60087912	rs573790	11	60088555	rs1441586	0.268978	1

BP, physical position (base pairs); CHR, chromosome; SNP, single nucleotide polymorphism.
