# Supplementary material for: SingleNucleotide Polymorphisms as Biomarkers of Mepolizumab and Benralizumab Treatment Response in Severe Eosinophilic Asthma
Source: Int J Mol Sci. 2024 Jul 26;25(15):8139. doi: 10.3390/ijms25158139 (PMC11311889; doi:10.3390/ijms25158139)
Supplement: Supplementary file 1 [file ijms-25-08139-s001.zip › Table S20.pdf]

Table S20. Association of clinical characteristics of mepolizumab-treated patients with response to the 3 criteria.

Characteristics	N	Response		X^2	p-value	Ref. Cat	OR	CI 95%
		R N (%)	NR N (%)					
Sex								
Female	48	15 (52.1)	23 (47.9)	0.695	0.405			
Male	24	10 (41.7)	14 (58.3)					
Age of initiation BT (years)	72	35 (48.6)	37 (51.4)		0.118			
Years with asthma	72	35 (48.6)	37 (51.4)		0.442			
BMI (kg/m2)								
<25	19	11 (57.9)	8 (42.1)	0.8905	0.345			
>25	53	24 (45.3)	29 (54.7)					
Previous respiratory disease								
Yes	34	15 (44.1)	19 (55.9)	0.5207	0.471			
No	38	20 (52.6)	18 (47.4)					
Tobacco consumption								
Non-smoker	60	27 (45)	33 (55)	1.8792	0.170			
Current smoker	0	(0)	0 (0)					
Former smoker	12	8 (66.7)	4 (33.3)					
Polyps								
Yes	33	14 (42.4)	19 (57.6)	0.9335	0.334			
No	39	21 (53.8)	18 (46.2)					
Allergies								
Yes	37	17 (45.9)	20 (54.1)	0.2164	0.642			
No	35	18 (51.4)	17 (48.6)					
GERD								
Yes	32	16 (50)	16 (50)	0.045	0.833			
No	40	19 (47.5)	21 (52.5)					
SAHS								
Yes	15	9 (60)	6 (60)	0.9838	0.321			
No	57	26 (45.6)	31 (54.4)					
COPD								
Yes	13	7 (53.8)	6 (46.2)	0.1740	0.677			
No	59	28 (47.5)	31 (52.5)					
Age of diagnosis (years)	72	35 (48.6)	37 (51.4)		0.127			
<18	2	0 (0)	2 (100)		0.493*			
>18	70	35 (50)	35 (50)					
ICS (µg/day)	72	35 (48.6)	37 (51.4)		0.199			
OCS cycles per year								
Yes	57	26 (45.6)	31 (54.4)	0.9838	0.321			
No	15	9 (60)	6 (40)					
Baseline FEV1 (%)								
<80	51	25 (49)	26 (51)	0.0117	0.914			
>80	21	10 (47.6)	11 (52.4)					
Exacerbation in previous year								
Yes	47	21 (44.7)	26 (55.3)	0.8370	0.360			
No	25	14 (56)	11 (44)					
Basal blood eosinophils (cell/mcl)								
<300	15	7 (46.7)	8 (53.3)	0.0287	0.8655			
>300	57	28 (49.1)	19 (50.9))					
Previous BT								
Yes	21	8 (38.1)	13 (61.9)	1.3124	0.252			
No	51	27 (52.9)	24 (47.1)					

BMI, body mass index; GERD, gastroesophageal reflux disease; SAHS, sleep apnea-hypopnea syndrome; COPD, chronic obstructive pulmonary disease; ICS, inhaled corticosteroids; OCS, oral corticosteroids; FEV1, maximum expiratory volume in the first second of forced expiration; BT, biological therapy.

Ref. Cat, Reference category; NR, Non-Responder; R, Responder; OR, Odds Ratio; CI 95%, Confidence interval; *p-value for Fisher's Exact Test.
