# Supplementary material for: SingleNucleotide Polymorphisms as Biomarkers of Mepolizumab and Benralizumab Treatment Response in Severe Eosinophilic Asthma
Source: Int J Mol Sci. 2024 Jul 26;25(15):8139. doi: 10.3390/ijms25158139 (PMC11311889; doi:10.3390/ijms25158139)
Supplement: Supplementary file 1 [file ijms-25-08139-s001.zip › Table S21.pdf]

Table S21. Association of mepolizumab genetic polymorphisms with response to the 3 criteria.

Gene	SNPs	Genotype	N	Response R N (%)	Response NR N (%)	χ²	p-value	Ref Cat	OR	CI 95%
IL1RL1	rs1420101	CC	26	13 (50)	13 (50)	2.3384	0.3106			
		CT	34	14 (41.2)	20 (58.8)					
		TT	12	8 (66.7)	4 (33.3)					
		C	60	27 (45)	33 (55)	1.8292	0.170			
		T	46	22 (47.8)	24 (52.2)	0.0314	0.859			
	rs17026974	AA	6	4 (66.7)	2 (33.3)		0.557*			
		AG	28	12 (42.9)	16 (57.1)					
		GG	38	19 (50)	19 (50)					
		A	34	16 (47.1)	18 (52.9)	0.0621	0.803			
		G	66	31 (47)	35 (53)		0.423*			
	rs1921622	AA	20	14 (70)	6 (30)	5.0971	0.078			
		AG	39	16 (41)	23 (59)					
		GG	13	5 (38.5)	8 (61.5)					
		A	59	30 (50.8)	29 (49.2)	0.6542	0.419			
		G	52	21 (40.4)	31 (59.6)	5.0714	0.024	G	3.44	1.18-11.09
IL5	rs4143832	GG	51	24 (47.1)	27 (52.9)		0.606*			
		GT	17	8 (47.1)	9 (52.9)					
		TT	4	3 (75)	1 (25)					
		G	68	32 (47.1)	36 (52.9)		0.351*			
		T	21	11 (52.4)	10 (47.6)	0.1687	0.681			
	rs17690122	AA	51	24 (47.1)	27 (52.9)		0.606*			
		AG	17	8 (47.1)	9 (52.9)					
		GG	4	3 (75)	1 (25)					
		A	68	32 (47.1)	36 (52.9)		0.351*			
		G	21	11 (52.4)	10 (47.6)	0.1687	0.681			
GATA2	rs4857855	CC	53	26 (49.1)	27 (50.9)		1*			
		CT	16	8 (50)	8 (50)					
		TT	3	1 (33.3)	2 (66.7)					
		C	69	34 (49.3)	35 (50.7)		1*			
		T	19	9 (47.4)	10 (52.6)	0.0159	0.899			
IKZF2	rs12619285	AA	36	19 (52.8)	17 (47.2)		0.761*			
		AG	31	14 (45.2)	17 (54.8)					
		GG	5	2 (40)	3 (60)					
		A	67	33 (49.3)	34 (50.7)		1*			
		G	26	16 (44.4)	20 (55.6)	0.5	0.479			
RAD50	rs11739623	CC	38	21 (55.3)	17 (44.7)		0.566*			
		CT	32	13 (40.6)	19 (59.4)					
		TT	2	1 (50)	1 (50)					
		C	70	34 (48.6)	36 (51.4)		1*			
		T	34	14 (41.2)	20 (58.8)	1.4254	0.233			
	rs4705959	CC	3	1 (33.3)	2 (66.7)		0.132*			
		CT	28	10 (35.7)	18 (64.3)					
		TT	41	24 (58.5)	17 (41.5)					
		C	31	11 (35.5)	20 (64.5)	3.755	0.053			
		T	69	34 (49.3)	35 (50.7)		1*			
FCER1A	rs2251746	CC	5	3 (60)	2 (40)		0.814*			
		CT	26	13 (50)	13 (50)					
		TT	41	19 (46.3)	22 (53.7)					
		C	31	16 (51.6)	15 (48.4)	0.1964	0.658			
		T	67	32 (47.8)	35 (52.2)		0.67*			
	rs2427837	AA	6	3 (50)	3 (50)		0.678*			
		AG	25	14 (56)	11 (44)					
		GG	41	18 (43.9)	23 (56.1)					
		A	31	17 (54.8)	14 (45.2)	0.8452	0.358			
		G	66	32 (48.5)	34 (51.5)		1*			
FCER1B	rs1441586	CC	11	5 (45.5)	6 (54.5)	0.4552	0.796			
		CT	41	19 (46.3)	22 (53.7)					
		TT	20	11 (55)	9 (45)					
		C	52	24 (46.2)	28 (53.8)	0.4525	0.501			
		T	61	30 (49.2)	31 (50.8)	0.0518	0.82			

Gene	SNPs	Genotype	N	Response		χ²	p-value	Ref Cat	OR	CI 95%
				R<br>N (%)	NR<br>N (%)					
FCER1B	rs573790	CC	30	13 (43.3)	17 (56.7)		0.560*			
		CT	36	18 (50)	18 (50)					
		TT	6	4 (66.7)	2 (33.3)					
		C	66	31 (47)	35 (53)		0.423			
		T	42	22 (52.4)	20 (47.6)	0.5735	0.449			
	rs569108	AA	63	35 (55.6)	28 (44.4)		0.002*	AG	5.3 x10^{7}	5.3 x10^{-31}-NA
		AG	9	0 (0)	9 (100)					
		GG	-	-	-					
		A								
		G	9	0 (0)	9 (100)		0.002*	G	5.3 x10^{7}	5.3 x10^{-31}-NA
ZNF415	rs1054485	GG	17	9 (52.9)	8 (47.1)	0.7027	0.704			
		GT	31	16 (51.6)	15 (48.4)					
		TT	24	10 (41.7)	14 (58.3)					
		G	48	25 (52.1)	23 (47.9)	0.695	0.405			
		T	55	26 (47.3)	29 (52.7)	0.167	0.683			
FCGR2A	rs1801274	AA	27	12 (44.4)	15 (55.6)	0.8384	0.658			
		AG	25	14 (56)	11 (44)					
		GG	20	9 (45)	11 (55)					
		A	52	26 (50)	26 (50)	0.1446	0.704			
		G	45	23 (51.1)	22 (48.9)	0.3	0.584			
FCGR2B	rs3219018	CC	1	0 (0)	1 (100)		0.319*			
		CG	24	14 (58.3)	10 (41.7)					
		GG	47	21 (44.7)	26 (55.3)					
		C	25	14 (56)	11 (44)	0.837	0.36			
		G	71	35 (49.3)	36 (50.7)		1*			
	rs1050501	CC	1	1 (100)	0 (0)		0.158*			
		CT	16	5 (31.2)	11 (68.8)					
		TT	55	29 (52.7)	26 (47.3)					
		C	17	6 (35.3)	11 (64.7)	1.5799	0.209			
		T	71	34 (47.9)	37 (52.1)		0.486*			
FCGR3A	rs10127939	AA	61	31 (50.8)	30 (49.2)		0.439*			
		AC	8	2 (25)	6 (75)					
		CC	3	2 (66.7)	1 (33.3)					
		A	69	33 (47.8)	36 (52.2)		0.609*			
		C	11	4 (36.4)	7 (63.6)	0.7796	0.377			
	rs396991	AA	22	11 (50)	11 (50)		0.843*			
		CA	41	19 (46.3)	22 (53.7)					
		CC	9	5 (46.3)	4 (44.4)					
		A	63	30 (55.6)	33 (52.4)		0.732*			
		C	50	24 (48)	26 (52)	0.0245	0.876			

Ref. Cat., reference category; R, responder; NR, non-responder; OR, odds ratio; CI 95%, 95% confidence Interval 95%; *p-value for Fisher exact test.
