# Supplementary material for: SingleNucleotide Polymorphisms as Biomarkers of Mepolizumab and Benralizumab Treatment Response in Severe Eosinophilic Asthma
Source: Int J Mol Sci. 2024 Jul 26;25(15):8139. doi: 10.3390/ijms25158139 (PMC11311889; doi:10.3390/ijms25158139)
Supplement: Supplementary file 1 [file ijms-25-08139-s001.zip › Table S22.pdf]

Table S22. Association of clinical characteristics of patients treated with benralizumab with reduction and/or absence of exacerbations.

Characteristics	N	Response R N (%)	NR N (%)	X^2	p-value	Ref. Cat	OR	CI 95%
Sex								
Female	34	31 (91.2)	3 (8.8)		0.542*			
Male	17	17 (100)	0 (0)					
Age of initiation BT (years)	51	48 (94.1)	3 (5.9)		0.969			
Years with asthma	51	48 (94.1)	3 (5.9)		0.981			
BMI (kg/m2)								
<25	9	9 (100)	0 (0)		1*			
>25	42	39 (92.9)	3 (7.1)					
Previous respiratory disease								
Yes	24	22 (91.7)	2 (8.3)		0.596*			
No	27	26 (96.3)	1 (3.7)					
Tobacco consumption								
Non-smoker	39	38 (97.4)	1 (2.6)		0.106*			
Current smoker	2	2 (100)	0 (0)					
Former smoker	10	8 (80)	2 (20)					
Polyps								
Yes	20	20 (100)	0 (0)		0.271*			
No	31	28 (90.3)	3 (9.7)					
Allergies								
Yes	33	31 (93.9)	2 (6.1)		1*			
No	18	17 (94.4)	1 (5.6)					
GERD								
Yes	22	19 (90.9)	2 (9.1)		0.571*			
No	29	28 (86.6)	1 (3.4)					
SAHS								
Yes	10	10 (100)	0 (0)		1*			
No	41	38 (92.7)	3 (7.3)					
COPD								
Yes	10	9 (90)	1 (10)		0.488*			
No	41	39 (95.1)	2 (4.9)					
Age of diagnosis (years)	51	48 (94.1)	3 (5.9)		0.961			
<18	1	1 (100)	0 (0)		1*			
>18	50	47 (94)	3 (6)					
ICS (µg/day)	51	48 (94.1)	3 (5.9)		0.564			
OCS cycles per year								
Yes	6	6 (100)	0 (0)		1*			
No	45	42 (93.3)	3 (6.7)					
Baseline FEV1 (%)								
<80	33	31 (93.9)	2 (6.1)		1*			
>80	17	16 (94.1)	1 (5.9)					
Exacerbation in previous year								
Yes	22	19 (86.4)	3 (13.6)		0.073*	Si	1.3×10^{8}	1.8×10^{-19}-NA
No	29	29 (100)	0 (0)					
Basal blood eosinophils (cell/mcl)								
<300	47	44 (93.6)	3 (6.4)		1*			
>300	4	4 (100)	0 (0)					
Previous BT								
Yes	20	19 (95)	1 (5)		1*			
No	31	29 (93.5)	2 (6.5)					

BMI: body mass index; COPD: chronic obstructive pulmonary disease; COPD: chronic obstructive pulmonary disease; GORD: gastro-oesophageal reflux disease; ICS: inhaled corticosteroids; OCS: oral corticosteroids; FEV1: peak expiratory volume in the first second of forced expiration; TB: biologic therapy; SAHS: sleep apnoea-hypopnoea syndrome.
Cat. Ref, reference category; NR, non-responder; R, responder; OR, Odds Ratio; 95% CI, confidence interval; *p value for Fisher's exact test.
