# Supplementary material for: SingleNucleotide Polymorphisms as Biomarkers of Mepolizumab and Benralizumab Treatment Response in Severe Eosinophilic Asthma
Source: Int J Mol Sci. 2024 Jul 26;25(15):8139. doi: 10.3390/ijms25158139 (PMC11311889; doi:10.3390/ijms25158139)
Supplement: Supplementary file 1 [file ijms-25-08139-s001.zip › Table S23.pdf]

Table S23. Association of benralizumab genetic polymorphisms with reduction and/or absence of exacerbations.

Gene	SNPs	Genotype	N	Response R N (%)	Response NR N (%)	χ²	p-value	Ref Cat	OR	CI 95%
IL1RL1	rs1420101	CC	18	17 (94.4)	1 (5.6)		1*			
		CT	27	25 (92.6)	2 (7.4)					
		TT	6	6 (100)	0 (0)					
		C	45	42 (93.3)	3 (6.7)		1*			
		T	33	31 (93.9)	2 (6.1)		1*			
	rs17026974	AA	4	4 (100)	0 (0)		0.649*			
		AG	18	16 (88.9)	2 (11.1)					
		GG	29	28 (96.6)	1 (3.4)					
		A	22	20 (90.9)	2 (9.1)		0.571*			
		G	47	44 (93.6)	3 (6.4)		1*			
	rs1921622	AA	11	11 (100)	0 (0)		1*			
		AG	29	27 (93.1)	2 (6.9)					
		GG	11	10 (90.9)	1 (9.1)					
		A	40	38 (95)	2 (1)		0.525*			
		G	40	37 (92.5)	3 (7.5)		1*			
IL5	rs4143832	GG	33	30 (90.9)	3 (9.1)		0.670*			
		GT	13	13 (100)	0 (0)					
		TT	5	5 (100)	0 (0)					
		G	45	42 (93.3)	3 (6.7)		1*			
		T	17	17 (100)	0 (0)		0.542*			
	rs17690122	AA	36	33 (91.7)	3 (8.3)		1*			
		AG	11	11 (100)	0 (0)					
		GG	4	4 (100)	0 (0)					
		A	47	44 (93.6)	3 (6.4)		1*			
		G	15	15 (100)	0 (0)		0.546*			
GATA2	rs4857855	CC	37	34 (91.9)	3 (8.1)		0.616*			
		CT	12	12 (100)	0 (0)					
		TT	2	2 (100)	0 (0)					
		C	49	46 (93.9)	3 (6.1)		1*			
		T	14	14 (100)	0 (0)		0.552*			
IKZF2	rs12619285	AA	24	24 (100)	0 (0)		0.173*			
		AG	19	17 (89.5)	2 (10.5)					
		GG	8	7 (87.5)	1 (12.5)					
		A	43	41 (95.3)	2 (4.7)		0.407*			
		G	27	24 (88.9)	3 (11.1)		0.238*			
RAD50	rs11739623	CC	26	24 (92.3)	2 (7.7)		1*			
		CT	22	20 (95.5)	1 (4.5)					
		TT	3	3 (100)	0 (0)					
		C	48	45 (93.8)	3 (6.2)		1*			
		T	25	24 (96)	1 (4)		1*			
	rs4705959	CC	3	3 (100)	0 (0)		1*			
		CT	19	18 (94.7)	1 (5.3)					
		TT	29	27 (93.1)	2 (6.9)					
		C	22	21 (95.5)	1 (4.5)		1*			
		T	48	45 (93.8)	3 (6.2)		1*			
FCER1A	rs2251746	CC	5	5 (100)	0 (0)		0.479*			
		CT	17	17 (100)	0 (0)					
		TT	29	26 (89.7)	3 (10.3)					
		C	22	22 (100)	0 (0)		0.246*			
		T	46	4 (93.5)	3 (6.5)		1*			
	rs2427837	AA	5	5 (100)	0 (0)		0.665*			
		AG	15	15 (100)	0 (0)					
		GG	31	28 (90.3)	3 (9.7)					
		A	20	20 (100)	0 (0)		0.265*			
		G	46	43 (93.5)	3 (6.5)		1*			
FCER1B	rs1441586	CC	11	11 (100)	0 (0)		0.770*			
		CT	30	28 (93.3)	2 (6.7)					
		TT	10	9 (90)	1 (10)					
		C	41	39 (95.1)	2 (4.9)		0.488*			
		T	40	37 (92.5)	3 (7.5)		1*			

Gene	SNPs	Genotype	N	Response		χ²	p-value	Ref Cat	OR	CI 95%
				R N (%)	NR N (%)					
FCER1B	rs573790	CC	21	20 (95.2)	1 (4.8)		1*			
		CT	27	25 (92.6)	2 (7.4)					
		TT	3	3 (100)	0 (0)					
		C	48	45 (93.8)	3 (6.2)		1*			
		T	30	28 (93.3)	2 (6.7)		1*			
	rs569108	AA	46	43 (93.5)	3 (6.5)		1*			
		AG	5	5 (100)	0 (0)					
		GG	-	-	-					
		A	-	-	-					
		G	5	5 (100)	0 (0)		1*			
ZNF415	rs1054485	GG	16	14 (87.5)	2 (12.5)		0.448*			
		GT	23	22 (95.7)	1 (4.3)					
		TT	12	12 (100)	0 (0)					
		G	39	36 (92.3)	3 (7.7)		1*			
		T	35	34 (97.1)	1 (2.9)		0.229*			
FCGR2A	rs1801274	AA	13	13 (100)	0 (0)		0.602*			
		AG	26	24 (92.3)	2 (7.7)					
		GG	12	11 (91.7)	1 (8.3)					
		A	39	37 (94.9)	2 (5.1)		0.534*			
		G	38	35 (92.1)	3 (7.9)		0.561*			
FCGR2B	rs3219018	CC	-	-	-		1*			
		CG	20	19 (95)	1 (5)					
		GG	31	29 (93.5)	2 (6.5)					
		C	20	19 (95)	1 (5)		1*			
		G	-	-	-					
	rs1050501	CC	-	-	-		0.546*			
		CT	15	15 (100)	0 (0)					
		TT	36	33 (91.7)	3 (8.3)					
		C	15	15 (100)	0 (0)		0.545*			
		T	-	-	-					
FCGR3A	rs10127939	AA	45	42 (93.3)	3 (6.7)		1*			
		AC	5	5 (100)	15					
		CC	1	1 (100)	15					
		A	50	47 (94)	3 (6)		1*			
		C	6	6 (100)	0 (0)		1*			
	rs396991	AA	12	11 (91.7)	1 (8.3)		1*			
		CA	34	32 (94.1)	2 (5.9)					
		CC	5	5 (100)	0 (0)					
		A	46	43 (93.5)	3 (6.5)		1*			
		C	39	37 (94.9)	2 (5.1)		0.561*			

Ref. Cat., reference category; R, responder; NR, non-responder; OR, odds ratio; CI 95%, 95% confidence Interval 95%; *p-value for Fisher exact test.
