# Supplementary material for: SingleNucleotide Polymorphisms as Biomarkers of Mepolizumab and Benralizumab Treatment Response in Severe Eosinophilic Asthma
Source: Int J Mol Sci. 2024 Jul 26;25(15):8139. doi: 10.3390/ijms25158139 (PMC11311889; doi:10.3390/ijms25158139)
Supplement: Supplementary file 1 [file ijms-25-08139-s001.zip › Table S24.pdf]

Table S24. Association of clinical characteristics of benralizumab-treated patients with reduction and/or absence of oral corticosteroids.

Characteristics	N	Response R N (%)	Response NR N (%)	X^2	p-value	Ref. Cat	OR	CI 95%
Sex								
Female	34	25 (73.5)	9 (26.5)	5.0748	0.024	Male	3.97	1.19-14.22
Male	17	7 (41.2)	10 (58.8)					
Age of initiation BT (years)	51	32 (62.7)	19 (37.3)		0.796			
Years with asthma	51	32 (62.7)	19 (37.3)		0.536			
BMI (kg/m2)								
<25	9	6 (66.7)	3 (33.3)	0.0719	0.789			
>25	42	26 (61.9)	16 (38.1)					
Previous respiratory disease								
Yes	24	14 (58.3)	10 (41.7)	0.3775	0.539			
No	27	18 (66.7)	9 (33.3)					
Tobacco consumption								
Non smoker	39	26 (66.7)	13 (33.3)		0.542*			
Current smoker	2	1 (50)	1 (50)					
Former smoker	10	5 (50)	5 (50)					
Polyps								
Yes	20	11 (55)	9 (45)	0.8443	0.358			
No	31	21 (67.7)	10 (32.3)					
Allergies								
Yes	33	25 (75.8)	8 (24.2)	6.7728	0.009	No	4.91	1.47-17.86
No	18	7 (38.9)	10 (61.1)					
GERD								
Yes	22	14 (63.6)	8 (36.4)	0.0131	0.909			
No	29	18 (62.1)	11 (37.9)					
SAHS								
Yes	10	6 (60)	4 (40)	0.04	0.841			
No	41	26 (63.4)	15 (36.6)					
COPD								
Yes	10	3 (30)	7 (70)	5.7058	0.017	Yes	5.64	1.33-29.76
No	41	29 (70.7)	12 (29.3)					
Age of diagnosis (years)	51	32 (62.7)	19 (37.3)		0.724			
<18	1	1 (100)	0 (0)		1*			
>18	50	31 (62)	19 (38)					
ICS (µg/day)	51	32 (62.7)	19 (37.3)		0.532			
OCS cycles per year								
Yes	6	6 (100)	0 (0)		0.072*			
No	45	26 (57.8)	19 (42.2)					
Baseline FEV1 (%)								
<80	34	21 (61.8)	13 (38.2)	0.0419	0.838			
>80	17	11 (64.7)	6 (35.3)					
Exacerbation in previous year								
Yes	22	14 (63.6)	8 (36.4)	0.0131	0.909			
No	29	18 (62.1)	11 (37.9)					
Basal blood eosinophils (cell/mcl)								
<300	47	30 (63.8)	17 (36.2)	0.3016	0.583			
>300	4	2 (50)	2 (50)					
Previous BT								
Yes	20	11 (55)	9 (45)	0.8443	0.358			
No	31	21 (67.7)	10 (32.3)					

BMI, body mass index; GERD, gastroesophageal reflux disease; SAHS, sleep apnea-hypopnea syndrome; COPD, chronic obstructive pulmonary disease; ICS, inhaled corticosteroids; OCS, oral corticosteroids; FEV1, maximum expiratory volume in the first second of forced expiration; BT, biological therapy.

Ref. Cat, Reference category; NR, Non-Responder; R, Responder; OR, Odds Ratio; CI 95%, Confidence interval; *p-value for Fisher's Exact Test.
