# Supplementary material for: SingleNucleotide Polymorphisms as Biomarkers of Mepolizumab and Benralizumab Treatment Response in Severe Eosinophilic Asthma
Source: Int J Mol Sci. 2024 Jul 26;25(15):8139. doi: 10.3390/ijms25158139 (PMC11311889; doi:10.3390/ijms25158139)
Supplement: Supplementary file 1 [file ijms-25-08139-s001.zip › Table S25.pdf]

Table S25. Association of benralizumab genetic polymorphisms with corticosteroid reduction and/or absence.

Gene	SNPs	Genotype	N	Response		χ²	p-value	Ref Cat	OR	CI 95%
				R<br>N (%)	NR<br>N (%)					
IL1RL1	rs1420101	CC	18	9 (50)	9 (50)					
		CT	27	19 (70.4)	8 (229.6)		0.383*			
		TT	6	4 (66.7)	2 (33.3)					
		C	45	28 (62.2)	17 (37.8)		1*			
		T	33	23 (69.7)	10 (30.3)	2.3926	0.122			
	rs17026974	AA	4	3 (75)	1 (25)					
		AG	18	13 (72.2)	5 (27.8)		0.49*			
		GG	29	16 (55.2)	13 (44.8)					
		A	22	16 (72.7)	6 (27.3)	1.6492	0.199			
		G	47	29 (61.7)	18 (38.3)		1*			
	rs1921622	AA	11	7 (63.6)	4 (36.4)					
		AG	29	20 (69)	9 (31)		0.489*			
		GG	11	5 (45.5)	6 (54.5)					
		A	40	27 (67.5)	13 (32.5)	1.7937	0.181			
		G	40	25 (62.5)	15 (37.5)	0.0018	0.945			
IL5	rs4143832	GG	33	18 (54.5)	15 (45.5)					
		GT	13	10 (76.9)	3 (23.1)		0.268*			
		TT	5	4 (80)	1 (20)					
		G	46	28 (60.9)	18 (39.1)		0.639*			
		T	18	14 (82.4)	4 (17.6)	2.6893	0.101			
	rs17690122	AA	36	21 (58.3)	15 (41.7)					
		AG	11	8 (72.7)	3 (27.3)		0.637*			
		GG	4	3 (75)	1 (25)					
		A	47	29 (61.7)	18 (38.3)		1*			
		G	15	11 (73.3)	4 (26.7)	1.0192	0.313			
GATA2	rs4857855	CC	37	23 (62.2)	14 (37.8)					
		CT	12	8 (66.7)	4 (33.3)		1*			
		TT	2	1 (50)	1 (50)					
		C	49	31 (63.3)	18 (36.7)		1*			
		T	14	9 (64.3)	5 (35.7)	0.0007	0.979			
IKZF2	rs12619285	AA	24	15 (62.5)	9 (37.5)					
		AG	19	14 (73.7)	5 (26.3)	3.1544	0.207			
		GG	8	3 (37.5)	5 (62.5)					
		A	43	29 (67.4)	14 (32.6)	2.5869	0.108			
		G	27	17 (63)	10 (37)	0.0012	0.973			
RAD50	rs11739623	CC	26	18 (69.2)	8 (30.8)					
		CT	22	13 (59.1)	9 (40.9)		0.431*			
		TT	3	1 (33.3)	2 (66.7)					
		C	48	31 (64.6)	17 (35.4)		0.548*			
		T	25	14 (56)	11 (44)	0.9544	0.329			
	rs4705959	CC	3	1 (33.3)	2 (66.7)					
		CT	19	10 (52.6)	9 (47.4)		0.216*			
		TT	29	21 (72.4)	8 (27.6)					
		C	22	11 (50)	11 (50)	2.6885	0.101			
		T	48	31 (64.6)	17 (35.4)		0.548*			
FCER1A	rs2251746	CC	5	3 (60)	2 (40)					
		CT	17	11 (64.7)	6 (35.3)		1*			
		TT	29	18 (62.1)	11 (37.69)					
		C	22	14 (63.6)	8 (36.4)	0.0131	0.909			
		T	46	29 (63)	17 (37)		1*			
	rs2427837	AA	5	3 (60)	2 (40)					
		AG	15	9 (60)	6 (40)		1*			
		GG	31	20 (64.5)	11 (35.5)					
		A	20	12 (60)	8 (40)	0.1061	0.745			
		G	46	29 (63)	17 (37)		1*			
FCER1B	rs1441586	CC	11	5 (45.5)	6 (54.5)					
		CT	30	19 (63.3)	11 (36.7)		0.295*			
		TT	10	8 (80)	2 (20)					
		C	41	24 (58.8)	17 (41.5)	1.5843	0.208			
		T	40	27 (67.5)	13 (32.5)	1.7937	0.181			

Gene	SNPs	Genotype	N	Response		χ²	p-value	Ref Cat	OR	CI 95%
				R N (%)	NR N (%)					
FCER1B	rs573790	CC	21	14 (66.7)	7 (33.3)		0.338*			
		CT	27	15 (55.6)	12 (44.4)					
		TT	3	3 (100)	0 (0)					
		C	48	29 (60.4)	19 (39.6)		0.285*			
		T	30	18 (60)	12 (40)	0.2349	0.628			
	rs569108	AA	46	31 (67.4)	15 (32.6)		0.05*	AG	8.27	1.11-169.42
		AG	5	1 (20)	4 (80)					
		GG	-	-	-					
		A	-	-	-					
		G	5	1 (20)	4 (80)		0.05*	G	8.27	1.11-169.42
ZNF415	rs1054485	GG	16	11 (68.8)	5 (31.2)	0.7068	0.702			
		GT	23	13 (56.5)	10 (43.5)					
		TT	12	8 (66.7)	4 (33.3)					
		G	39	24 (61.5)	15 (38.5)	0.1032	0.748			
		T	35	21 (60)	14 (40)	0.3596	0.549			
FCGR2A	rs1801274	AA	13	7 (53.8)	6 (46.2)		0.245*			
		AG	26	15 (57.7)	11 (42.3)					
		GG	12	10 (83.3)	2 (16.7)					
		A	39	22 (56.4)	17 (43.6)	2.8455	0.091			
		G	38	25 (65.8)	13 (34.2)	0.5911	0.442			
FCGR2B	rs3219018	CC	-	-	-	0.0145	0.904			
		CG	20	13 (65)	7 (35)					
		GG	30	19 (63.3)	11 (36.7)					
		C	20	13 (65)	7 (35)	0.0145	0.904			
		G	-	-	-					
	rs1050501	CC	-	-	-	0.1398	0.709			
		CT	15	10 (66.7)	5 (33.3)					
		TT	36	22 (61.1)	14 (38.9)					
		C	15	10 (66.7)	5 (33.3)	0.1398	0.709			
		T	-	-	-					
FCGR3A	rs10127939	AA	45	27 (60)	18 (40)		0.772*			
		AC	5	4 (80)	1 (20)					
		CC	1	1 (100)	0 (0)					
		A	50	31 (62)	19 (38)		1*			
		C	6	5 (83.3)	1 (16.7)		0.391*			
	rs396991	AA	12	10 (83.3)	2 (16.7)		0.138*			
		CA	34	18 (52.9)	16 (47.1)					
		CC	5	4 (80)	1 (20)					
		A	46	28 (60.9)	18 (39.1)		0.639*			
		C	39	22 (56.4)	17 (436)	2.8455	0.092			

Ref. Cat., reference category; R, responder; NR, non-responder; OR, odds ratio; CI 95%, 95% confidence Interval 95%; *p-value for Fisher exact test.
