# Supplementary material for: SingleNucleotide Polymorphisms as Biomarkers of Mepolizumab and Benralizumab Treatment Response in Severe Eosinophilic Asthma
Source: Int J Mol Sci. 2024 Jul 26;25(15):8139. doi: 10.3390/ijms25158139 (PMC11311889; doi:10.3390/ijms25158139)
Supplement: Supplementary file 1 [file ijms-25-08139-s001.zip › Table S26.pdf]

Table S26. Association of clinical characteristics of benralizumab-treated patients with improvement in lung function.

Characteristics	N	Response		X^2	p-value	Ref. Cat	OR	CI 95%
		R N (%)	NR N (%)					
Sex								
Female	34	25 (73.5)	9 (26.5)	0.0492	0.824			
Male	17	12 (70.6)	5 (29.4)					
Age of initiation BT (years)	51	37 (72.5)	14 (27.5)		0.244			
Years with asthma	51	37 (72.5)	14 (27.5)		0.675			
BMI (kg/m2)								
<25	9	7 (77.8)	2 (22.2)	0.15	0.699			
>25	42	30 (71.4)	12 (28.6)					
Previous respiratory disease								
Yes	24	17 (70.8)	7 (29.2)	0.067	0.796			
No	27	20 (74.1)	7 (25.9)					
Tobacco consumption								
Non-smoker	39	30 (76.9)	9 (23.1)		0.091*			
Current smoker	2	0 (0)	2 (100)					
Former smoker	10	7 (70)	3 (30)					
Polyps								
Yes	20	18 (90)	2 (10)	5.0314	0.025	No	5.68	1.31-39.9
No	31	19 (61.3)	12 (38.7)					
Allergies								
Yes	33	24 (72.7)	9 (27.3)	0.0015	0.969			
No	18	13 (72.2)	5 (27.8)					
GERD								
Yes	22	18 (81.8)	4 (18.2)	1.6691	0.196			
No	29	19 (65.5)	10 (34.5)					
SAHS								
Yes	10	7 (70)	3 (30)	0.0406	0.84			
No	41	30 (73.2)	11 (26.8)					
COPD								
Yes	10	7 (70)	3 (30)	0.0406	0.84			
No	41	30 (73.2)	11 (26.8)					
Age of diagnosis (years)	51	37 (72.5)	14 (27.5)		0.295			
<18	1	0 (0)	1 (100)		0.275*			
>18	50	37 (74)	13 (26)					
ICS (µg/day)	51	37 (72.5)	14 (27.5)		0.461			
OCS cycles per year								
Yes	45	33 (73.3)	12 (26.7)		0.661*			
No	6	4 (66.7)	2 (33.3)					
Baseline FEV1 (%)								
<80	34	22 (64.7)	12 (35.3)	3.1506	0.076	<80	4.09	0.94-28.78
>80	17	15 (88.2)	2 (11.8)					
Exacerbation in previous year								
Yes	22	13 (59.1)	9 (40.9)	3.5186	0.061	Yes	3.32	0.95-12.85
No	29	24 (82.8)	5 (17.2)					
Basal blood eosinophils (cell/mcl)								
<300	47	34 (72.3)	13 (27.7)	0.0131	0.909			
>300	4	3 (75)	1 (25)					
Previous BT								
Yes	20	14 (70)	6 (30)	0.1074	0743			
No	31	23 (74.2)	8 (25.8)					

BMI, body mass index; GERD, gastroesophageal reflux disease; SAHS, sleep apnea-hypopnea syndrome; COPD, chronic obstructive pulmonary disease; ICS, inhaled corticosteroids; OCS, oral corticosteroids; FEV1, maximum expiratory volume in the first second of forced expiration; BT, biological therapy.
Ref. Cat, Reference category; NR, Non-Responder; R, Responder; OR, Odds Ratio; CI 95%, Confidence interval; *p-value for Fisher's Exact Test.
