# Supplementary material for: SingleNucleotide Polymorphisms as Biomarkers of Mepolizumab and Benralizumab Treatment Response in Severe Eosinophilic Asthma
Source: Int J Mol Sci. 2024 Jul 26;25(15):8139. doi: 10.3390/ijms25158139 (PMC11311889; doi:10.3390/ijms25158139)
Supplement: Supplementary file 1 [file ijms-25-08139-s001.zip › Table S27.pdf]

Table S27. Association of benralizumab genetic polymorphisms with improved lung function (FEV1).

Gene	SNPs	Genotype	N	Response		χ²	p-value	Ref Cat	OR	CI 95%
				R N (%)	NR N (%)					
IL1RL1	rs1420101	CC	18	13 (72.2)	5 (27.8)		0.305*			
		CT	27	18 (66.7)	9 (33.3)					
		TT	6	6 (100)	0 (0)					
		C	45	31 (68.9)	14 (31.1)		0.17*			
		T	33	24 (72.7)	9 (27.3)	0.0015	0.969			
	rs17026974	AA	4	4 (100)	0 ()		0.573*			
		AG	18	12 (66.7)	6 (33.3)					
		GG	29	21 (72.4)	8 (27.6)					
		A	22	16 (72.7)	6 (27.3)	0.0006	0.98			
		G	47	33 (70.2)	14 (29.8)		0.565*			
	rs1921622	AA	11	9 (81.8)	2 (18.2)		0.637*			
		AG	29	21 (72.4)	8 (27.6)					
		GG	11	7 (63.6)	4 (36.4)					
		A	40	30 (75)	10 (25)	0.5594	0.455			
		G	40	28 (70)	12 (30)	0.6051	0.437			
IL5	rs4143832	GG	33	21 (63.6)	12 (36.4)		0.165*			
		GT	13	11 (84.6)	2 (15.4)					
		TT	5	5 (100)	0 (0)					
		G	46	32 (69.6)	14 (30.4)		0.562*			
		T	18	16 (88.9)	2 (11.1)	3.7294	0.053	GG	4.57	1.05-32.12
	rs17690122	AA	36	24 (66.7)	12 (33.3)		0.393*			
		AG	11	9 (81.8)	2 (18.2)					
		GG	3	4 (100)	0 (0)					
		A	47	33 (70.2)	14 (29.8)		0.565*			
		G	15	13 (86.7)	2 (13.3)	2.1266	0.145			
GATA2	rs4857855	CC	37	26 (70.3)	11 (29.7)		0.413*			
		CT	12	10 (83.3)	2 (16.7)					
		TT	2	1 (50)	1 (50)					
		C	49	36 (73.5)	13 (26.5)		0.478*			
		T	14	11 (78.6)	3 (21.4)	0.3514	0.553			
IKZF2	rs12619285	AA	24	21 (87.5)	3 (12.5)	5.5776	0.061	GG	7	1.15-49.93
		AG	19	17 (63.2)	7 (36.8)				1.71	0.31-9.55
		GG	8	4 (50)	4 (50)				1	
		A	43	33 (76.7)	10 (23.3)	2.4225	0.12			
		G	27	26 (59.3)	11 (40.7)	5.0882	0.024	G	4.81	1.26-23.97
RAD50	rs11739623	CC	26	19 (73.1)	7 (26.9)		1*			
		CT	22	16 (72.7)	6 (27.3)					
		TT	3	2 (66.7)	1 (33.3)					
		C	48	35 (72.9)	13 (27.1)		1*			
		T	25	18 (72)	7 (28)	0.0074	0.931			
	rs4705959	CC	3	2 (66.7)	1 (33.3)		0.884*			
		CT	19	13 (68.4)	6 (31.6)					
		TT	29	22 (75.9)	7 (24.1)					
		C	22	15 (68.2)	7 (31.8)	0.3705	0.543			
		T	48	35 (72.9)	13 (27.1)		1*			
FCER1A	rs2251746	CC	5	4 (80)	1 (20)		0.577*			
		CT	17	14 (82.4)	3 (17.6)					
		TT	29	19 (65.5)	10 (34.5)					
		C	22	18 (81.8)	4 (18.2)	1.6691	0.196			
		T	46	33 (71.7)	13 (28.3)		1*			
	rs2427837	AA	5	4 (80)	1 (20)		0.328*			
		AG	15	13 (86.7)	2 (13.3)					
		GG	31	20 (64.5)	11 (35.5)					
		A	20	17 (85)	3 (15)	2.5613	0.11			
		G	46	33 (71.7)	13 (28.3)		1*			
FCER1B	rs1441586	CC	11	9 (81.8	2 (18.2)		0.217*			
		CT	30	23 (76.7)	7 (23.3)					
		TT	10	5 (50)	5 (50)					
		C	41	32 (78)	9 (22)	3.1758	0.074	TT	3.56	0.82-15.67
		T	40	28 (70)	12 (30)	0.6051	0.437			

Gene	SNPs	Genotype	N	Response		χ²	p-value	Ref Cat	OR	CI 95%
				R N (%)	NR N (%)					
FCER1B	rs573790	CC	21	14 (66.7)	7 (33.3)		1*			
		CT	27	20 (74.1)	7 (25.9)					
		TT	3	3 (100)	0 (0)					
		C	42	30 (71.4)	12 (28.6)		1*			
		T	24	18 (75)	6 (25)	0.1375	0.69			
	rs569108	AA	46	33 (80)	13 (20)		1*			
		AG	5	4 (80)	1 (20)					
		GG	-	-	-					
		A	-	-	-					
		G	5	4 (80)	1 (20)		1*			
ZNF415	rs1054485	GG	16	10 (62.5)	6 (37.5)		0.568*			
		GT	23	18 (78.3)	5 (21.7)					
		TT	12	9 (75)	3 (25)					
		G	39	28 (71.8)	11 (28.2)	0.0473	0.828			
		T	35	27 (77.1)	8 (22.9)	1.1822	0.277			
FCGR2A	rs1801274	AA	13	11 (84.6)	2 (15.4)		0.601*			
		AG	26	18 (69.2)	8 (30.8)					
		GG	12	8 (66.7)	4 (33.3)					
		A	39	29 (74.4)	10 (25.6)	0.2095	0.647			
		G	38	23 (68.4)	12 (31.6)	1.2755	0.259			
FCGR2B	rs3219018	CC	-	-	-	0.0993	0.753			
		CG	20	15 (75)	5 (25)					
		GG	31	22 (71)	9 (29)					
		C	20	15 (75)	5 (25)	0.0993	0.753			
		G	-	-	-					
	rs1050501	CC	-	-	-	0.5924	0.442			
		CT	15	12 (80)	3 (20)					
		TT	36	25 (69.4)	11 (30.6)					
		C	15	12 (80)	3 (20)	0.5924	0.442			
		T	-	-	-					
FCGR3A	rs10127939	AA	45	31 (68.9)	14 (31.1)		0.495*			
		AC	5	5 (100)	0 (0)					
		CC	1	1 (100)	0 (0)					
		A	50	36 (72)	14 (28)		1*			
		C	6	6 (100)	0 (0)		0.17*			
	rs396991	AA	12	9 (75)	3 (25)		0.383*			
		CA	34	23 (67.6)	11 (32.4)					
		CC	5	5 (100)	0 (0)					
		A	46	32 (69.6)	14 (30.4)		0.301*			
		C	39	28 (71.8)	11 (28.2)	0.0473	0.828			

Ref. Cat., reference category; R, responder; NR, non-responder; OR, odds ratio; CI 95%, 95% confidence Interval 95%; *p-value for Fisher exact test.
