# Supplementary material for: SingleNucleotide Polymorphisms as Biomarkers of Mepolizumab and Benralizumab Treatment Response in Severe Eosinophilic Asthma
Source: Int J Mol Sci. 2024 Jul 26;25(15):8139. doi: 10.3390/ijms25158139 (PMC11311889; doi:10.3390/ijms25158139)
Supplement: Supplementary file 1 [file ijms-25-08139-s001.zip › Table S28.pdf]

Table S28. Association of clinical characteristics of benralizumab-treated patients with response to at least one parameter.

Characteristics	N	Response R N (%)	Response NR N (%)	X^2	p-value	Ref. Cat	OR	CI 95%
Sex								
Female	34	33 (97.1)	1 (2.9)		1*			
Male	17	17 (100)	0 (0)					
Age of initiation BT (years)	51	50 (98)	1 (2)		0.529			
Years with asthma	51	50 (98)	1 (2)		0.372			
BMI (kg/m2)								
<25	9	9 (100)	0 (0)		1*			
>25	42	41 (97.6)	1 (2.4)					
Previous respiratory disease								
Yes	24	23 (95.8)	1 (4.2)		0.471*			
No	27	27 (100)	0 (0)					
Tobacco consumption								
Non-smoker	39	39 (100)	0 (0)		0.235*			
Current smoker	2	2 (100)	0 (0)					
Former smoker	10	9 (90)	1 (10)					
Polyps								
Yes	20	20 (100)	0 (0)		1*			
No	31	30 (96.8)	1 (3.2)					
Allergies								
Yes	33	33 (100)	0 (0)		0.353*			
No	18	17 (94.4)	1 (5.6)					
GERD								
Yes	22	22 (100)	0 (0)		1*			
No	29	28 (96.6)	1 (3.4)					
SAHS								
Yes	10	10 (100)	0 (0)		1*			
No	41	40 (97.6)	1 (2.4)					
COPD								
Yes	10	9 (90)	1 (10)		0.196*			
No	41	41 (100)	0 (0)					
Age of diagnosis (years)	51	50 (98)	1 (2)		0.793			
<18	1	1 (100)	0 (0)		1*			
>18	50	49 (98)	1 (2)					
ICS (µg/day)	51	50 (98)	1 (2)		0.502			
OCS cycles per year								
Yes	45	44 (97.8)	1 (2.2)		1*			
No	6	6 (100)	0 (0)					
Baseline FEV1 (%)								
<80	34	33 (97.1)	1 (2.9)		1*			
>80	17	17 (100)	0 (0)					
Exacerbation in previous year								
Yes	22	21 (95.5)	1 (4.5)		0.431*			
No	29	29 (100)	0 (0)					
Basal blood eosinophils (cell/mcl)								
<300	47	46 (97.9)	1 (2.1)		1*			
>300	4	4 (100)	0 (0)					
Previous BT								
Yes	20	20 (100)	0 (0)		1*			
No	31	30 (96.8)	1 (3.2)					

BMI, body mass index; GERD, gastroesophageal reflux disease; SAHS, sleep apnea-hypopnea syndrome; COPD, chronic obstructive pulmonary disease; ICS, inhaled corticosteroids; OCS, oral corticosteroids; FEV1, maximum expiratory volume in the first second of forced expiration; BT, biological therapy.
Ref. Cat, Reference category; NR, Non-Responder; R, Responder; OR, Odds Ratio; CI 95%, Confidence interval; *p-value for Fisher's Exact Test.
