# Supplementary material for: SingleNucleotide Polymorphisms as Biomarkers of Mepolizumab and Benralizumab Treatment Response in Severe Eosinophilic Asthma
Source: Int J Mol Sci. 2024 Jul 26;25(15):8139. doi: 10.3390/ijms25158139 (PMC11311889; doi:10.3390/ijms25158139)
Supplement: Supplementary file 1 [file ijms-25-08139-s001.zip › Table S29.pdf]

Table S29. Association of benralizumab genetic polymorphisms with responders to at least one criterion.

| Gene | SNPs | Genotype | N | Response R N (%) | Response NR N (%) | χ² | p-value | Ref Cat | OR | CI 95% |
|---|---|---|---|---|---|---|---|---|---|---|
| IL1RL1 | rs1420101 | CC | 18 | 17 (94.4) | 1 (5.6) | | 0.470* | | | |
| | | CT | 27 | 27 (100) | 0 (0) | | | | | |
| | | TT | 6 | 6 (100) | 0 (0) | | | | | |
| | | C | 45 | 44 (97.8) | 1 (2.2) | | 1* | | | |
| | | T | 33 | 33 (100) | 0 (0) | | 0.353* | | | |
| | rs17026974 | AA | 4 | 4 (100) | 0 (0) | | 1* | | | |
| | | AG | 18 | 18 (100) | 0 (0) | | | | | |
| | | GG | 29 | 28 (96.6) | 1 (3.4) | | | | | |
| | | A | 22 | 22 (100) | 0 (0) | | 1* | | | |
| | | G | 47 | 46 (97.9) | 1 (2.1) | | 1* | | | |
| | rs1921622 | AA | 11 | 11 (100) | 0 (0) | | 0.431* | | | |
| | | AG | 29 | 29 (100) | 0 (0) | | | | | |
| | | GG | 11 | 10 (90.9) | 1 (9.1) | | | | | |
| | | A | 40 | 40 (100) | 0 (0) | | 0.216* | | | |
| | | G | 40 | 39 (97.5) | 1 (2.5) | | 1* | | | |
| IL5 | rs4143832 | GG | 33 | 32 (97) | 1 (3) | | 1* | | | |
| | | GT | 13 | 13 (100) | 0 (0) | | | | | |
| | | TT | 5 | 5 (100) | 0 (0) | | | | | |
| | | G | 46 | 45 (97.8) | 1 (2.2) | | 1* | | | |
| | | T | 18 | 18 (100) | 0 (0) | | 1* | | | |
| | rs17690122 | AA | 36 | 35 (97.2) | 1 (2.8) | | 1* | | | |
| | | AG | 11 | 11 (100) | 0 (0) | | | | | |
| | | GG | 4 | 4 (100) | 0 (0) | | | | | |
| | | A | 47 | 46 (97.9) | 1 (2.1) | | 1* | | | |
| | | G | 15 | 15 (100) | 0 (0) | | 1* | | | |
| GATA2 | rs4857855 | CC | 37 | 36 (97.3) | 1 (2.7) | | 1* | | | |
| | | CT | 12 | 12 (100) | 0 (0) | | | | | |
| | | TT | 2 | 2 (100) | 0 (0) | | | | | |
| | | C | 49 | 48 (98) | 1 (2) | | 1* | | | |
| | | T | 14 | 14 (100) | 0 (0) | | 1* | | | |
| IKZF2 | rs12619285 | AA | 24 | 24 (100) | 0 (0) | | 0.157* | | | |
| | | AG | 19 | 19 (100) | 0 (0) | | | | | |
| | | GG | 8 | 7 (87.5) | 1 (12.5) | | | | | |
| | | A | 43 | 43 (100) | 0 (0) | | 0.157* | | | |
| | | G | 27 | 26 (96.3) | 1 (3.7) | | 1* | | | |
| RAD50 | rs11739623 | CC | 26 | 25 (96.2) | 1 (3.8) | | 1* | | | |
| | | CT | 22 | 22 (100) | 0 (0) | | | | | |
| | | TT | 3 | 3 (100) | 0 (0) | | | | | |
| | | C | 48 | 47 (97.9) | 1 (2.1) | | 1* | | | |
| | | T | 25 | 25 (100) | 0 (0) | | 1* | | | |
| | rs4705959 | CC | 3 | 3 (100) | 0 (0) | | 1* | | | |
| | | CT | 19 | 19 (100) | 0 (0) | | | | | |
| | | TT | 29 | 28 (96.6) | 1 (3.4) | | | | | |
| | | C | 21 | 21 (100) | 0 (0) | | 1* | | | |
| | | T | 48 | 47 (97.9) | 1 (2.1) | | 1* | | | |
| FCER1A | rs2251746 | CC | 5 | 5 (100) | 0 (0) | | 1* | | | |
| | | CT | 17 | 17 (100) | 0 (0) | | | | | |
| | | TT | 29 | 28 (96.6) | 1 (3.4) | | | | | |
| | | C | 22 | 22 (100) | 0 (0) | | 1* | | | |
| | | T | 46 | 45 (97.8) | 1 (2.2) | | 1* | | | |
| | rs2427837 | AA | 5 | 5 (100) | 0 (0) | | 1* | | | |
| | | AG | 15 | 15 (100) | 0 (0) | | | | | |
| | | GG | 31 | 30 (96.8) | 1 (3.2) | | | | | |
| | | A | 20 | 20 (100) | 0 (0) | | 1* | | | |
| | | G | 46 | 45 (97.8) | 1 (2.2) | | 1* | | | |
| FCER1B | rs1441586 | CC | 11 | 11 (100) | 0 (0) | | 0.196* | | | |
| | | CT | 30 | 30 (100) | 0 (0) | | | | | |
| | | TT | 10 | 9 (90) | 1 (10) | | | | | |
| | | C | 41 | 41 (100) | 0 (0) | | 0.196* | | | |
| | | T | 40 | 39 (97.5) | 1 (2.5) | | 1* | | | |
| | rs573790 | CC | 21 | 21 (100) | 0 (0) | | 1* | | | |

| Gene | SNPs | Genotype | N | Response |  | χ² | p-value | Ref Cat | OR | CI 95% |
|---|---|---|---|---|---|---|---|---|---|---|
|  |  |  |  | R<br>N (%) | NR<br>N (%) |  |  |  |  |  |
|  |  | CT | 27 | 26 (96.3) | 1 (3.7) |  |  |  |  |  |
|  |  | TT | 3 | 3 (100) | 0 (0) |  |  |  |  |  |
|  |  | C | 48 | 47 (97.9) | 1 (2.1) |  | 1* |  |  |  |
|  |  | T | 30 | 29 (96.1) | 1 (3.3) |  | 1* |  |  |  |
|  | rs569108 | AA | 46 | 45 (97.8) | 1 (2.2) |  | 1* |  |  |  |
|  |  | AG | 5 | 5 (100) | 0 (0) |  |  |  |  |  |
|  |  | GG | 0 | 0 (0) | 0 (0) |  |  |  |  |  |
|  |  | A | - | - | - |  |  |  |  |  |
|  |  | G | 5 | 5 (100) | 0 (0) |  | 1* |  |  |  |
| ZNF415 | rs1054485 | GG | 16 | 15 (93.8) | 1 (6.2) |  | 0.549* |  |  |  |
|  |  | GT | 23 | 23 (100) | 0 (0) |  |  |  |  |  |
|  |  | TT | 12 | 12 (100) | 0 (0) |  |  |  |  |  |
|  |  | G | 39 | 38 (97.4) | 1 (2.6) |  | 1* |  |  |  |
|  |  | T | 35 | 35 (100) | 0 (0) |  | 0.314* |  |  |  |
| FCGR2A | rs1801274 | AA | 13 | 13 (100) | 0 (0) |  | 1* |  |  |  |
|  |  | AG | 26 | 25 (96.2) | 1 (3.8) |  |  |  |  |  |
|  |  | GG | 12 | 12 (100) | 0 (0) |  |  |  |  |  |
|  |  | A | 39 | 38 (97.4) | 1 (2.6) |  | 1* |  |  |  |
|  |  | G | 38 | 37 (97.4) | 1 (2.6) |  | 1* |  |  |  |
| FCGR2B | rs3219018 | CC | - | - | - |  | 0.417* |  |  |  |
|  |  | CG | 20 | 19 (95) | 1 (5) |  |  |  |  |  |
|  |  | GG | 31 | 31 (100) | 0 (0) |  |  |  |  |  |
|  |  | C | 20 | 19 (95) | 1 (5) |  | 0.417* |  |  |  |
|  |  | G | - | - | - |  |  |  |  |  |
|  | rs1050501 | CC | - | - | - |  | 1* |  |  |  |
|  |  | CT | 15 | 15 (100) | 0 (0) |  |  |  |  |  |
|  |  | TT | 36 | 35 (97.2) | 1 (2.8) |  |  |  |  |  |
|  |  | C | 15 | 15 (100) | 0 (0) |  | 1* |  |  |  |
|  |  | T | - | - | - |  |  |  |  |  |
| FCGR3A | rs10127939 | AA | 45 | 44 (97.8) | 1 (2.2) |  | 1* |  |  |  |
|  |  | AC | 5 | 5 (100) | 0 (0) |  |  |  |  |  |
|  |  | CC | 1 | 1 (100) | 0 (0) |  |  |  |  |  |
|  |  | A | 50 | 49 (98) | 1 (2) |  | 1* |  |  |  |
|  |  | C | 6 | 6 (100) | 0 (0) |  | 1* |  |  |  |
|  | rs396991 | AA | 12 | 12 (100) | 0 (0) |  | 1* |  |  |  |
|  |  | CA | 34 | 33 (97.1) | 1 (2.9) |  |  |  |  |  |
|  |  | CC | 5 | 5 (100) | 0 (0) |  |  |  |  |  |
|  |  | A | 46 | 45 (97.8) | 1 (2.2) |  | 1* |  |  |  |
|  |  | C | 39 | 38 (97.4) | 1 (2.6) |  | 1* |  |  |  |

Ref. Cat., reference category; R, responder; NR, non-responder; OR, odds ratio; CI 95%, 95% confidence Interval 95%; *p-value for Fisher exact test.
