# Supplementary material for: SingleNucleotide Polymorphisms as Biomarkers of Mepolizumab and Benralizumab Treatment Response in Severe Eosinophilic Asthma
Source: Int J Mol Sci. 2024 Jul 26;25(15):8139. doi: 10.3390/ijms25158139 (PMC11311889; doi:10.3390/ijms25158139)
Supplement: Supplementary file 1 [file ijms-25-08139-s001.zip › Table S3.pdf]

Table S3. Minor allele frequencies for the SNPs studied.

CHR	SNP	Gene	Minor allele	Major allele	MAF
1	rs2427837	FCER1A	A	G	0.2569
1	rs2251746	FCER1A	C	T	0.25
1	rs1801274	FCGR2A	G	A	0.4514
1	rs396991	FCGR3A	C	A	0.4097
1	rs10127939	FCGR3A	C	A	0.09722
1	rs3219018	FCGR2B	C	G	0.1806
1	rs1050501	FCGR2B	C	T	0.125
2	rs17026974	IL1RL1	A	G	0.2778
2	rs1420101	IL1RL1	T	C	0.4028
2	rs1921622	IL1RL1	G	A	0.4514
2	rs12619285	IKZF2	G	A	0.2847
3	rs4857855	GATA2	T	C	0.1528
5	rs4143832	IL5	T	G	0.1736
5	rs11739623	RAD50	T	C	0.25
5	rs4705959	RAD50	C	T	0.2361
5	rs17690122	IL5	G	A	0.1736
11	rs573790	FCER1B	T	C	0.3333
11	rs1441586	FCER1B	C	T	0.4375
11	rs569108	FCER1B	G	A	0.0625
19	rs1054485	ZNF415	G	T	0.4514

Chr, chromosome; SNP, single nucleotide polymorphism; MAF, minor allele frequency.
