# Supplementary material for: SingleNucleotide Polymorphisms as Biomarkers of Mepolizumab and Benralizumab Treatment Response in Severe Eosinophilic Asthma
Source: Int J Mol Sci. 2024 Jul 26;25(15):8139. doi: 10.3390/ijms25158139 (PMC11311889; doi:10.3390/ijms25158139)
Supplement: Supplementary file 1 [file ijms-25-08139-s001.zip › Table S30.pdf]

Table S30. Association of clinical characteristics of patients treated with benralizumab with response on at least 2 parameters.

Characteristics	N	Response R N (%)	Response NR N (%)	X^2	p-value	Ref. Cat	OR	CI 95%
Sex								
Female	34	29 (85.3)	5 (14.7)	0.6071	0.436			
Male	17	13 (76.5)	4 (23.5)					
Age of initiation BT (years)	51	42 (82.4)	9 (17.6)		0.377			
Years with asthma	51	42 (82.4)	9 (17.6)		0.97			
BMI (kg/m2)								
<25	9	8 (88.9)	1 (11.1)	0.3212	0.571			
>25	42	34 (81)	8 (19)					
Previous respiratory disease								
Yes	24	18 (75)	6 (25)		0.13*			
No	27	24 (88.9)	3 (11.1)					
Tobacco consumption								
Non-smoker	39	33 (84.6)	6 (15.4)		0.341*			
Current smoker	2	1 (50)	1 (50)					
Former smoker	10	8 (80)	2 (20)					
Polyps								
Yes	20	19 (95)	1 (5)	3.6213	0.057	No	6.61	1.07-128.28
No	31	23 (74.2)	8 (25.8)					
Allergies								
Yes	33	28 (84.8)	5 (15.2)	0.4007	0.527			
No	18	14 (77.8)	4 (22.2)					
GERD								
Yes	22	18 (81.8)	4 (18.2)	0.0076	0.931			
No	29	24 (82.8)	5 (17.2)					
SAHS								
Yes	10	8 (80)	2 (20)	0.0474	0.828			
No	41	34 (82.9)	7 (17.1)					
COPD								
Yes	10	7 (70)	3 (30)	1.3061	0.253			
No	41	35 (85.4)	6 (14.6)					
Age of diagnosis (years)	51	42 (82.4)	9 (17.6)		0.368			
<18	1	1 (100)	0 (0)		1*			
>18	50	41 (82)	9 (18)					
ICS (µg/day)	51	42 (82.4)	9 (17.6)		0.131			
OCS cycles per year								
Yes	6	6 (100)	0 (0)		0.575*			
No	45	36 (80)	9 (20)					
Baseline FEV1 (%)								
<80	34	26 (76.5)	8 (23.5)	2.4286	0.119			
>80	17	16 (94.1)	1 (5.9)					
Exacerbation in previous year								
Yes	22	15 (68.2)	7 (31.8)	5.3463	0.021	Si	6.3	1.32-46.06
No	29	27 (93.1)	2 (6.9)					
Basal blood eosinophils (cell/mcl)								
<300	47	39 (83)	8 (17)		0.552*			
>300	4	3 (75)	1 (25)					
Previous BT								
Yes	20	15 (75)	5 (25)	1.2241	0.269			
No	31	27 (87.1)	4 (12.9)					

BMI, body mass index; GERD, gastroesophageal reflux disease; SAHS, sleep apnea-hypopnea syndrome; COPD, chronic obstructive pulmonary disease; ICS, inhaled corticosteroids; OCS, oral corticosteroids; FEV1, maximum expiratory volume in the first second of forced expiration; BT, biological therapy.

Ref. Cat, Reference category; NR, Non-Responder; R, Responder; OR, Odds Ratio; CI 95%, Confidence interval; *p-value for Fisher's Exact Test.
