# Supplementary material for: SingleNucleotide Polymorphisms as Biomarkers of Mepolizumab and Benralizumab Treatment Response in Severe Eosinophilic Asthma
Source: Int J Mol Sci. 2024 Jul 26;25(15):8139. doi: 10.3390/ijms25158139 (PMC11311889; doi:10.3390/ijms25158139)
Supplement: Supplementary file 1 [file ijms-25-08139-s001.zip › Table S31.pdf]

Table S31. Association of benralizumab genetic polymorphisms with 2-criteria response.

Gene	SNPs	Genotype	N	Response		χ²	p-value	Ref Cat	OR	CI 95%
				R<br>N (%)	NR<br>N (%)					
IL1RL1	rs1420101	CC	18	13 (72.2)	5 (27.8)		0.383*			
		CT	27	23 (85.2)	4 (14.8)					
		TT	6	6 (100)	0 (0)					
		C	45	36 (80)	9 (20)		0.575			
		T	33	29 (87.9)	4 (12.1)	1.9645	0.161			
	rs17026974	AA	4	4 (100)	0 (0)		1*			
		AG	18	15 (83.3)	3 (16.7)					
		GG	29	23 (79.3)	6 (20.7)					
		A	22	19 (86.4)	3 (13.6)	0.4282	0.513			
		G	47	38 (80.9)	9 (19.1)		1*			
	rs1921622	AA	1	11 (100)	0 (0)		0.202*			
		AG	29	23 (79.3)	6 (20.7)					
		GG	11	8 (72.7)	3 (27.3)					
		A	40	34 (85)	6 (15)	0.8942	0.344			
		G	40	31 (77.5)	9 (22.5)	3.0054	0.083			
IL5	rs4143832	GG	33	25 (75.8)	8 (24.2)		0.381*			
		GT	13	12 (92.3)	1 (7.7)					
		TT	5	5 (100)	0 (0)					
		G	46	37 (80.4)	9 (19.6)		0.571*			
		T	18	17 (94.4)	1 (5.6)	2.7986	0.094			
	rs17690122	AA	36	28 (77.8)	8 (22.2)		0.588*			
		AG	11	10 (90.9)	1 (9.1)					
		GG	4	4 (100)	0 (0)					
		A	47	38 (80.9)	9 (19.1)		1*			
		G	15	14 (93.3)	1 (6.7)	1.763	0.184			
GATA2	rs4857855	CC	37	30 (81.1)	7 (18.9)		0.355*			
		CT	12	11 (91.7)	1 (8.3)					
		TT	2	1 (50)	1 (50)					
		C	49	41 (83.7)	8 (16.3)		0.325*			
		T	14	12 (85.7)	2 (14.3)	0.15	0.699			
IKZF2	rs12619285	AA	24	23 (95.8)	1 (4.2)		0.038*	GG	13.79	1.44-314.47
		AG	19	14 (73.7)	5 (26.3)				9.68	1.58-188.03
		GG	8	5 (62.5)	3 (37.5)				1	
		A	43	37 (86)	6 (14)	2.5733	0.109			
		G	24	19 (70.4)	8 (29.6)		0.026*	G	9.47	1.49-184.89
RAD50	rs11739623	CC	26	21 (80.8)	5 (19.2)		0.488*			
		CT	22	19 (86.4)	3 (13.6)					
		TT	3	2 (66.7)	1 (33.3)					
		C	48	40 (83.3)	8 (16.7)		0.448*			
		T	25	21 (84)	4 (16)		1*			
	rs4705959	CC	3	2 (66.7)	1 (33.3)		0.697*			
		CT	19	16 (84.2)	3 (15.8)					
		TT	29	24 (82.8)	5 (17.2)					
		C	22	18 (81.8)	4 (18.2)	0.0076	0.931			
		T	48	40 (83.3)	8 (16.7)		0.449*			
FCER1A	rs2251746	CC	5	4 (80)	1 (20)		0.245*			
		CT	17	16 (94.1)	1 (5.9)					
		TT	29	22 (75.9)	7 (24.1)					
		C	22	20 (90.9)	2 (9.1)	1.9489	0.163			
		T	46	38 (82.6)	8 (17.4)		1*			
	rs2427837	AA	5	4 (80)	1 (20)		0.398*			
		AG	15	14 (93.3)	1 (6.7)					
		GG	31	24 (77.4)	7 (22.6)					
		A	20	18 (90)	2 (10)	1.324	0.25			
		G	46	38 (82.6)	8 (17.4)		1*			
FCER1B	rs1441586	CC	11	9 (81.8)	2 (18.2)		1*			
		CT	30	25 (83.3)	5 (16.7)					
		TT	10	8 (80)	2 (20)					
		C	41	34 (82.9)	7 (17.1)	0.0474	0.828			
		T	40	33 (82.5)	7 (17.5)	0.0028	0.958			

Gene	SNPs	Genotype	N	Response		χ²	p-value	Ref Cat	OR	CI 95%
				R N (%)	NR N (%)					
FCER1B	rs573790	CC	21	18 (85.7)	3 (14.3)		0.841*			
		CT	27	21 (77.8)	6 (22.2)					
		TT	3	3 (100)	0 (0)					
		C	48	39 (81.2)	9 (18.8)		1*			
		T	30	24 (80)	6 (20)	0.2776	0.598			
	rs569108	AA	46	38 (82.6)	8 (17.4)		1*			
		AG	5	4 (80)	1 (20)					
		GG	-	-	-					
		A	-	-	-					
		G	5	4 (80)	1 (20)		1*			
ZNF415	rs1054485	GG	16	12 (75)	4 (25)		0.512*			
		GT	23	19 (82.6)	4 (17.4)					
		TT	12	11 (91.7)	1 (8.3)					
		G	39	31 (79.5)	8 (20.5)	0.9367	0.333			
		T	35	30 (85.7)	5 (14.3)	0.8674	0.352			
FCGR2A	rs1801274	AA	13	11 (84.6)	2 (15.4)		1*			
		AG	26	21 (80.8)	5 (19.2)					
		GG	12	10 (83.3)	2 (16.7)					
		A	39	32 (82.1)	7 (17.9)	0.0104	0.919			
		G	38	31 (81.6)	7 (18.4)	0.0615	0.804			
FCGR2B	rs3219018	CC	-	-	-	0.1254	0.723			
		CG	20	16 (80)	4 (20)					
		GG	31	26 (83.9)	5 (16.1)					
		C	20	16 (80)	4 (20)		0.703*			
		G	-	-	-					
	rs1050501	CC	-	-	-	1.763	0.184			
		CT	15	14 (93.3)	1 (6.7)					
		TT	36	28 (77.8)	8 (22.2)					
		C	15	14 (93.3)	1 (6.7)	1.763	0.184			
		T	-	-	-					
FCGR3A	rs10127939	AA	45	36 (80)	9 (20)		0.646*			
		AC	5	5 (100)	0 (0)					
		CC	1	1 (100)	0 (0)					
		A	50	41 (82)	9 (18)		1*			
		C	6	6 (100)	0 (0)		0.575			
	rs396991	AA	12	11 (91.7)	1 (8.3)		0.434*			
		CA	34	26 (76.5)	8 (23.5)					
		CC	5	5 (100)	0 (0)					
		A	46	37 (80.4)	9 (19.6)		0.571*			
		C	39	31 (79.5)	8 (20.5)	0.9367	0.333			

Ref. Cat., reference category; R, responder; NR, non-responder; OR, odds ratio; CI 95%, 95% confidence Interval 95%; *p-value for Fisher exact test.
