# Supplementary material for: SingleNucleotide Polymorphisms as Biomarkers of Mepolizumab and Benralizumab Treatment Response in Severe Eosinophilic Asthma
Source: Int J Mol Sci. 2024 Jul 26;25(15):8139. doi: 10.3390/ijms25158139 (PMC11311889; doi:10.3390/ijms25158139)
Supplement: Supplementary file 1 [file ijms-25-08139-s001.zip › Table S32.pdf]

Table S32. Association of clinical characteristics of benralizumab-treated patients with response to the 3 criteria.

Characteristics	N	Response		X^2	p-value	Ref. Cat	OR	CI 95%
		R<br>N (%)	NR<br>N (%)					
Sex								
Female	34	19 (55.9)	15 (44.1)	0.9223	0.166			
Male	17	6 (35.3)	11 (64.7)					
Age of initiation BT (years)	51	25 (49)	26 (51)		0.907			
Years with asthma	51	25 (49)	26 (51)		0.417			
BMI (kg/m2)								
<25	9	5 (55.6)	4 (44.4)		0.727*			
>25	42	20 (47.6)	22 (52.4)					
Previous respiratory disease								
Yes	24	12 (50)	12 (50)	0.0174	0.895			
No	27	13 (48.1)	14 (51.9)					
Tobacco consumption								
Non-smoker	39	22 (56.4)	17 (43.6)		0.118			
Current smoker	2	0 (0)	2 (100)					
Former smoker	10	3 (30)	7 (70)					
Polyps								
Yes	20	10 (50)	10 (50)	0.0127	0.91			
No	31	15 (48.4)	16 (51.6)					
Allergies								
Yes	33	19 (57.6)	14 (42.4)	2.739	0.098			
No	18	6 (33.3)	12 (66.7)					
GERD								
Yes	22	12 (54.5)	10 (45.5)	0.4727	0.492			
No	29	13 (44.8)	16 (55.2)					
SAHS								
Yes	10	5 (50)	5 (50)	0.0048	0.945			
No	41	20 (48.8)	21 (51.2)					
COPD								
Yes	10	3 (30)	7 (70)	1.8006	0.18			
No	41	22 (53.7)	19 (46.3)					
Age of diagnosis (years)	51	25 (49)	26 (51)		0.940			
<18	1	0 (0)	1 (100)		1*			
>18	50	25 (50)	25 (50)					
ICS (µg/day)	51	25 (49)	26 (51)		0.517			
OCS cycles per year								
Yes	45	21 (46.7)	24 (53.3)		0.419*			
No	6	4 (66.7)	2 (33.3)					
Baseline FEV1 (%)								
<80	34	16 (47.1)	18 (52.9)	0.1569	0.692			
>80	17	9 (52.9)	8 (47.1)					
Exacerbation in previous year								
Yes	22	10 (45.5)	12 (54.5)	0.1968	0.657			
No	29	15 (51.7)	14 (48.3)					
Basal blood eosinophils (cell/mcl)								
<300	47	23 (48.9)	24 (51.1)		1*			
>300	4	2 (50)	2 (50)					
Previous BT								
Yes	20	9 (45)	11 (55)	0.2127	0.645			
No	31	16 (51.6)	15 (48.4)					

BMI, body mass index; GERD, gastroesophageal reflux disease; SAHS, sleep apnea-hypopnea syndrome; COPD, chronic obstructive pulmonary disease; ICS, inhaled corticosteroids; OCS, oral corticosteroids; FEV1, maximum expiratory volume in the first second of forced expiration; BT, biological therapy.

Ref. Cat, Reference category; NR, Non-Responder; R, Responder; OR, Odds Ratio; CI 95%, Confidence interval; *p-value for Fisher's Exact Test.
