# Supplementary material for: SingleNucleotide Polymorphisms as Biomarkers of Mepolizumab and Benralizumab Treatment Response in Severe Eosinophilic Asthma
Source: Int J Mol Sci. 2024 Jul 26;25(15):8139. doi: 10.3390/ijms25158139 (PMC11311889; doi:10.3390/ijms25158139)
Supplement: Supplementary file 1 [file ijms-25-08139-s001.zip › Table S33.pdf]

Table S33. Association of benralizumab genetic polymorphisms with response to the 3 criteria.

| Gene | SNPs | Genotype | N | Response<br>R<br>N (%) | <br>NR<br>N (%) | χ² | p-value | Ref Cat | OR | CI 95% |
|---|---|---|---|---|---|---|---|---|---|---|
| | | CC | 18 | 9 (50) | 9 (50) | | | | | |
| | | CT | 27 | 12 (44.4) | 15 (55.6) | | 0.624* | | | |
| | rs1420101 | TT | 6 | 4 (66.7) | 2 (33.3) | | | | | |
| | | C | 45 | 21 (46.7) | 24 (53.3) | | 0.419* | | | |
| | | T | 33 | 16 (48.5) | 17 (51.5) | 0.0107 | 0.918 | | | |
| | | AA | 4 | 3 (75) | 1 (25) | | | | | |
| | | AG | 18 | 8 (44.4) | 10 (55.6) | | 0.694* | | | |
| IL1RL1 | rs17026974 | GG | 29 | 14 (48.3) | 15 (51.7) | | | | | |
| | | A | 22 | 11 (50) | 11 (50) | 0.0149 | 0.903 | | | |
| | | G | 47 | 22 (46.8) | 25 (53.2) | | 0.35* | | | |
| | | AA | 11 | 5 (45.5) | 6 (54.5) | | | | | |
| | | AG | 29 | 16 (55.2) | 13 (44.8) | 1.2003 | 0.549 | | | |
| | rs1921622 | GG | 11 | 4 (36.4) | 7 (63.6) | | | | | |
| | | A | 40 | 21 (52.5) | 19 (47.5) | | 1* | | | |
| | | G | 40 | 20 (50) | 20 (50) | | 1* | | | |
| | | GG | 33 | 12 (36.4) | 21 (63.6) | | | | 1 | |
| | | GT | 13 | 9 (69.2) | 4 (30.8) | | 0.046* | GG | 3.94 | 1.05-17.21 |
| | rs4143832 | TT | 5 | 4 (80) | 1 (20) | | | | 7 | 0.91-145.61 |
| | | G | 46 | 21 (45.7) | 25 (54.3) | | 0.195* | | | |
| | | T | 18 | 13 (72.2) | 5 (27.8) | 5.993 | 0.014 | GG | 4.55 | 1.36-17.22 |
| IL5 | | AA | 36 | 15 (41.7) | 21 (58.3) | | | | | |
| | | AG | 11 | 7 (63.6) | 4 (36.4) | | 0.292* | | | |
| | rs17690122 | GG | 4 | 3 (75) | 1 (25) | | | | | |
| | | A | 47 | 22 (46.8) | 25 (53.2) | | 0.35* | | | |
| | | G | 15 | 10 (66.7) | 5 (33.3) | 2.648 | 0.104 | | | |
| | | CC | 37 | 17 (45.9) | 20 (54.1) | | | | | |
| | | CT | 12 | 7 (58.3) | 5 (41.7) | | 0.307* | | | |
| GATA2 | rs4857855 | TT | 2 | 1 (50) | 1 (50) | | | | | |
| | | C | 49 | 24 (49) | 25 (51) | | 1* | | | |
| | | T | 14 | 8 (57.1) | 6 (49.2) | 0.5096 | 0.475 | | | |
| | | AA | 24 | 13 (54.2) | 11 (45.8) | | | | | |
| | | AG | 19 | 10 (52.6) | 9 (47.4) | | 0.389* | | | |
| IKZF2 | rs12619285 | GG | 8 | 2 (25) | 6 (75) | | | | | |
| | | A | 43 | 23 (53.5) | 20 (46.5) | | 0.249* | | | |
| | | G | 27 | 12 (44.4) | 15 (55.6) | 0.4506 | 0.488 | | | |
| | | CC | 26 | 15 (57.7) | 11 (42.3) | | | | | |
| | | CT | 22 | 9 (40.9) | 13 (59.1) | | 0.478* | | | |
| | rs11739623 | TT | 3 | 1 (33.3) | 2 (66.7) | | | | | |
| | | C | 48 | 24 (50) | 24 (50) | | 1* | | | |
| | | T | 25 | 10 (40) | 15 (60) | 1.5964 | 0.206 | | | |
| RAD50 | | CC | 3 | 1 (33.3) | 2 (66.7) | | | | | |
| | | CT | 19 | 6 (31.6) | 13 (68.4) | | 0.087 | | | |
| | rs4705959 | TT | 29 | 18 (62.1) | 11 (37.9) | | | | | |
| | | C | 22 | 7 (31.8) | 15 (68.2) | 4.5809 | 0.032 | C | 3.51 | 1.12-11.85 |
| | | T | 48 | 24 (50) | 24 (50) | | 1* | | | |
| | | CC | 5 | 3 (60) | 2 (40) | | | | | |
| | | CT | 17 | 9 (52.9) | 8 (47.1) | | 0.772* | | | |
| | rs2251746 | TT | 29 | 13 (44.8) | 16 (55.2) | | | | | |
| | | C | 22 | 12 (54.5) | 10 (45.5) | 0.4727 | 0.492 | | | |
| | | T | 46 | 22 (47.8) | 24 (522) | | 0.668* | | | |
| FCER1A | | AA | 4 | 3 (60) | 2 (40) | | | | | |
| | | AG | 15 | 8 (53.3) | 7 (46.7) | | 0.766* | | | |
| | rs2427837 | GG | 31 | 14 (45.2) | 17 (54.8) | | | | | |
| | | A | 20 | 11 (55) | 9 (45) | 0.4709 | 0.493 | | | |
| | | G | 46 | 22 (47.8) | 24 (522) | | 0.668* | | | |
| | | CC | 11 | 5 (45.5) | 6 (54.5) | | | | | |
| | | CT | 30 | 15 (50) | 15 (50) | 0.0713 | 0.965 | | | |
| FCER1B | rs1441586 | TT | 10 | 5 (50) | 5 (50) | | | | | |
| | | C | 41 | 20 (48.8) | 21 (51.2) | 0.0048 | 0.945 | | | |
| | | T | 40 | 20 (50) | 20 (50) | 0.0713 | 0.789 | | | |

| Gene | SNPs | Genotype | N | Response | | χ² | p-value | Ref Cat | OR | CI 95% |
|---|---|---|---|---|---|---|---|---|---|---|
| | | | | R N (%) | NR N (%) | | | | | |
| FCER1B | rs573790 | CC | 21 | 9 (45) | 11 (55) | | 0.245* | | | |
| | | CT | 27 | 13 (48.1) | 14 (51.9) | | | | | |
| | | TT | 3 | 3 (100) | 0 (0) | | | | | |
| | | C | 48 | 22 (45.8) | 26 (54.2) | | 0.11* | | | |
| | | T | 30 | 16 (53.3) | 14 (46.7) | 0.543 | 0.461 | | | |
| | rs569108 | AA | 46 | 24 (52.2) | 22 (47.8) | | 0.35* | | | |
| | | AG | 5 | 1 (20) | 4 (80) | | | | | |
| | | GG | - | - | - | | | | | |
| | | A | - | - | - | | | | | |
| | | G | 5 | 1 (20) | 4 (80) | | 0.347* | | | |
| ZNF415 | rs1054485 | GG | 16 | 8 (50) | 8 (50) | 0.0239 | 0.988 | | | |
| | | GT | 23 | 11 (47.8) | 12 (52.2) | | | | | |
| | | TT | 12 | 6 (50) | 6 (50) | | | | | |
| | | G | 39 | 19 (48.7) | 20 (51.3) | 0.006 | 0.938 | | | |
| | | T | 35 | 17 (48.6) | 18 (51.4) | 0.009 | 0.925 | | | |
| FCGR2A | rs1801274 | AA | 13 | 7 (53.8) | 6 (46.2) | 1.0064 | 0.605 | | | |
| | | AG | 26 | 11 (42.3) | 15 (57.7) | | | | | |
| | | GG | 12 | 7 (58.3) | 5 (41.7) | | | | | |
| | | A | 39 | 18 (46.2) | 21 (53.8 | 0.5447 | 0.461 | | | |
| | | G | 38 | 18 (47.4) | 20 (52.6) | 0.1626 | 0.687 | | | |
| FCGR2B | rs3219018 | CC | - | - | - | 1.5875 | 0.208 | | | |
| | | CG | 20 | 12 (60) | 8 (40) | | | | | |
| | | GG | 31 | 13 (41.9) | 18 (58.1) | | | | | |
| | | C | 20 | 12 (60) | 8 (40) | 1.5875 | 0.208 | | | |
| | | G | - | - | - | | | | | |
| | rs1050501 | CC | - | - | - | 0.1582 | 0.691 | | | |
| | | CT | 15 | 8 (53.3) | 7 (46.7) | | | | | |
| | | TT | 36 | 17 (47.2) | 19 (52.8) | | | | | |
| | | C | 15 | 8 (53.3) | 7 (46.7) | 0.1582 | 0.691 | | | |
| | | T | - | - | - | | | | | |
| FCGR3A | rs10127939 | AA | 45 | 20 (44.4) | 25 (55.6) | | 0.115* | | | |
| | | AC | 5 | 4 (80) | 1 (20) | | | | | |
| | | CC | 1 | 1 (100) | 0 (0) | | | | | |
| | | A | 50 | 24 (48) | 26 (52) | | 0.49* | | | |
| | | C | 6 | 5 (83.3) | 1 (16.7) | | 0.099* | | | |
| | rs396991 | AA | 12 | 7 (58.3) | 5 (41.7) | | 0.209* | | | |
| | | CA | 34 | 14 (41.2) | 20 (58.8) | | | | | |
| | | CC | 5 | 4 (80) | 1 (20) | | | | | |
| | | A | 46 | 21 (45.7) | 25 (54.3) | | 0.191* | | | |
| | | C | 39 | 18 (46.2) | 21 (53.8) | 0.545 | 0.461* | | | |

Ref. Cat., reference category; R, responder; NR, non-responder; OR, odds ratio; CI 95%, 95% confidence Interval 95%; *p-value for Fisher exact test.
