# Supplementary material for: SingleNucleotide Polymorphisms as Biomarkers of Mepolizumab and Benralizumab Treatment Response in Severe Eosinophilic Asthma
Source: Int J Mol Sci. 2024 Jul 26;25(15):8139. doi: 10.3390/ijms25158139 (PMC11311889; doi:10.3390/ijms25158139)
Supplement: Supplementary file 1 [file ijms-25-08139-s001.zip › Table S4.pdf]

Table S4. Estimation of IL5 rs1413832/rs17690122 haplotype frequency in patients treated with mepolizumab.

	rs2251746	rs2427837	Freq	R	NR	Cumulative frequency	OR (95% CI)	p-value
**Responsive for 1 criterion**								
1	G	A	0.8264	0.8286	0.75	0.8264	1.00	---
2	T	G	0.1736	0.1714	0.25	1	1.49 (0.18 -12.16)	0.71
Global haplotype association p-value: 0.72								
**Responsive for 2 criteria**								
1	G	A	0.8264	0.8333	0.8	0.8264	1.00	---
2	T	G	0.1736	0.1667	0.2	1	1.21 (0.47 - 3.09)	0.69
Global haplotype association p-value: 0.07								
**Responsive for 3 criteria**								
1	G	A	0.8264	0.8	0.8514	0.8264	1.00	---
2	T	G	0.1736	0.2	0.1486	1	0.74 (0.33 - 1.65)	0.46
Global haplotype association p-value: 0.45								
**Reduction in OCS ≥ 50%**								
1	G	A	0.8264	0.8298	0.82	0.8264	1.00	---
2	T	G	0.1736	0.1702	0.18	1	1.06 (0.46 - 2.43)	0.89
Global haplotype association p-value: 0.89								
**Reduction in exacerbations ≥ 50%**								
1	G	A	0.8264	0.8231	0.8571	0.8264	1.00	---
2	T	G	0.1736	0.1769	0.1429	1	0.80 (0.19 - 3.44)	0.77
Global haplotype association p-value: 0.76								
**Increase in %FEV1 ≥ 10% or %FEV1 ≥ 80%**								
1	G	A	0.8264	0.8302	0.8158	0.8264	1.00	---
2	T	G	0.1736	0.1698	0.1842	1	1.09 (0.45 - 2.64)	0.85
Global haplotype association p-value: 0.85								

Freq: haplotype frequency; NA, not available; R, responder; NR, non-responder.
