# Supplementary material for: SingleNucleotide Polymorphisms as Biomarkers of Mepolizumab and Benralizumab Treatment Response in Severe Eosinophilic Asthma
Source: Int J Mol Sci. 2024 Jul 26;25(15):8139. doi: 10.3390/ijms25158139 (PMC11311889; doi:10.3390/ijms25158139)
Supplement: Supplementary file 1 [file ijms-25-08139-s001.zip › Table S5.pdf]

Table S5. Estimation of *FCER1A* rs2427837/rs2251746 haplotype frequency in patients treated with mepolizumab.

	**rs2251746**	**rs2427837**	**Freq**	**R**	**NR**	**Cumulative frequency**	**OR (95% CI)**	**p-value**
**Responsive for 1 criterion**								
1	T	G	0.736	0.7357	0.75	0.736	1.00	---
2	C	A	0.243	0.25	NA	0.979	0.00 (-Inf - Inf)	1
3	T	A	0.014	0.0143	NA	0.993	0.00 (-Inf - Inf)	1
4	C	G	0.007	NA	0.25	1	$1.1x10^{11}$ ($1.1x10^{11}$ – $1.1x10^{11}$)	<0.0001
Global haplotype association p-value: 0.03								
**Responsive for 2 criteria**								
1	T	G	0.736	0.7105	0.8333	0.736	1.00	---
2	C	A	0.243	0.2719	0.1333	0.979	0.46 (0.15 - 1.42)	0.18
3	T	A	0.014	0.0175	NA	0.993	0.00 (-Inf - Inf)	1
4	C	G	0.007	NA	0.0333	1	6 $x10^{21}$ (6 $x10^{21}$- 6 $x10^{21}$)	<0.0001
Global haplotype association p-value: 0.1								
**Responsive for 3 criteria**								
1	T	G	0.736	0.7143	0.7566	0.736	1.00	---
2	C	A	0.243	0.2714	0.216	0.979	0.78 (0.37 - 1.64)	0.51
3	T	A	0.014	0.0143	0.0137	0.993	0.97 (0.06 - 16.29)	0.99
4	C	G	0.007	NA	0.0137	1	1.9 $x10^{8}$ (1.9 $x10^{8}$ -1.9 $x10^{8}$)	<0.0001
Global haplotype association p-value: 0.62								
**Reduction in OCS ≥ 50%**								
1	T	G	0.736	0.7234	0.76	0.736	0.7234	0.76
2	C	A	0.243	0.2553	0.22	0.979	0.2553	0.22
3	T	A	0.014	0.0213	NA	0.993	0.0213	NA
4	C	G	0.007	NA	0.02	1	1 $x10^{41}$ (1 $x10^{41}$-1 $x10^{41}$)	<0.0001
Global haplotype association p-value: 0.27								
**Reduction in exacerbations ≥ 50%**								
1	T	G	0.736	0.7385	0.7143	0.736	1.00	---
2	C	A	0.243	0.2462	0.2143	0.979	1.02 (0.27 - 3.84)	0.98
3	T	A	0.014	0.0154	NA	0.993	0.00 (-Inf - Inf)	1
4	C	G	0.007	NA	0.0714	1	2.5 $x10^{18}$ (2.5 $x10^{18}$ -2.5 $x10^{18}$)	<0.0001
Global haplotype association p-value: 0.16								
**Increase in %FEV1 ≥ 10% or %FEV1 ≥ 80%**								
1	G	T	0.4238	0.527	0.3854	0.4238	1.00	---
2	G	C	0.3193	0.1605	0.3854	0.7431	**3.48 (1.07 - 11.35)**	0.043
3	A	T	0.1387	0.1605	0.1146	0.8818	0.87 (0.24 - 3.18)	0.83
4	A	C	0.1182	0.152	0.1146	1	1.37 (0.37 - 4.99)	0.64
Global haplotype association p-value: 0.063								

Freq: haplotype frequency; NA, not available; R, responder; NR, non-responder.
