# Supplementary material for: SingleNucleotide Polymorphisms as Biomarkers of Mepolizumab and Benralizumab Treatment Response in Severe Eosinophilic Asthma
Source: Int J Mol Sci. 2024 Jul 26;25(15):8139. doi: 10.3390/ijms25158139 (PMC11311889; doi:10.3390/ijms25158139)
Supplement: Supplementary file 1 [file ijms-25-08139-s001.zip › Table S6.pdf]

Table S6. Estimation of *RAD50* rs11739623/rs4705959 haplotype frequency in patients treated with mepolizumab.

	rs11739623	rs4705959	Total	R	NR	Cumulative frequency	OR (95% CI)	p-value
**Responsive for 1 criterion**								
1	C	T	0.7429	0.7355	1	0.7429	1.00	---
2	T	C	0.229	0.2355	NA	0.9719	0.00 (-Inf - Inf)	1
3	T	T	0.021	0.0216	NA	0.9929	0.00 (-Inf - Inf)	1
4	C	C	0.0071	0.0073	NA	1	0.85 (-Inf - Inf)	1
Global haplotype association p-value: 0.46								
**Responsive for 2 criteria**								
1	C	T	0.7429	0.7366	0.7667	0.7429	1.00	---
2	T	C	0.229	0.2278	0.2333	0.9719	0.98 (0.35 - 2.77)	0.98
3	T	T	0.021	0.0266	NA	0.9929	0.00 (-Inf - Inf)	1
4	C	C	0.0071	0.009	NA	1	0.85 (-Inf - Inf)	1
Global haplotype association p-value: 0.59								
**Responsive for 3 criteria**								
1	C	T	0.7429	0.7857	0.7027	0.7429	1.00	---
2	T	C	0.229	0.1714	0.2838	0.9719	1.82 (0.75 - 4.44)	0.19
3	T	T	0.021	0.0429	NA	0.9929	0.00 (-Inf - Inf)	1
4	C	C	0.0071	NA	0.0135	1	9.7×10^{59} (9.7×10^{59} -9.7×10^{59})	<0.0001
Global haplotype association p-value: 0.056								
**Reduction in OCS ≥ 50%**								
1	C	T	0.7429	0.7766	0.68	0.7429	1.00	---
2	T	C	0.229	0.1915	0.3	0.9719	1.75 (0.71 - 4.29)	0.23
3	T	T	0.021	0.0319	NA	0.9929	0.00 (-Inf - Inf)	1
4	C	C	0.0071	NA	0.02	1	9×10^{159} (9×10^{159} - 9×10^{159})	<0.0001
Global haplotype association p-value: 0.1								
**Reduction in exacerbations ≥ 50%**								
1	C	T	0.7429	0.7306	0.8571	0.7429	1.00	---
2	T	C	0.229	0.2383	0.1429	0.9719	0.47 (0.09 - 2.40)	0.36
3	T	T	0.021	0.0233	NA	0.9929	0.00 (-Inf - Inf)	1
4	C	C	0.0071	0.0079	NA	1	0.00 (-Inf - Inf)	1
Global haplotype association p-value: 0.62								
**Increase in %FEV1 ≥ 10% or %FEV1 ≥ 80%**								
1	C	T	0.7429	0.7261	0.7895	0.7429	1.00	---
2	T	C	0.229	0.2355	0.2105	0.9719	0.80 (0.30 - 2.11)	0.65
3	T	T	0.021	0.0286	NA	0.9929	0.00 (-Inf - Inf)	1
4	C	C	0.0071	0.0097	NA	1	0.00 (-Inf - Inf)	1
Global haplotype association p-value: 0.43								

Freq: haplotype frequency; NA, not available; R, responder; NR, non-responder.
