# Supplementary material for: SingleNucleotide Polymorphisms as Biomarkers of Mepolizumab and Benralizumab Treatment Response in Severe Eosinophilic Asthma
Source: Int J Mol Sci. 2024 Jul 26;25(15):8139. doi: 10.3390/ijms25158139 (PMC11311889; doi:10.3390/ijms25158139)
Supplement: Supplementary file 1 [file ijms-25-08139-s001.zip › Table S7.pdf]

Table S7. Estimation of IL5 rs1413832/rs17690122 haplotype frequency in patients treated with benralizumab.

	rs2251746	rs2427837	Freq	R	NR	Cumulative frequency	OR (95% CI)	p-value
**Responsive for 1 criterion**								
-	-	-	-	-	-	-		
**Responsive for 2 criteria**								
1	G	A	0.7745	0.7381	0.9444	0.7745	1.00	---
2	T	G	0.1863	0.2143	0.0556	0.9608	0.27 (0.04 - 1.95)	0.2
3	T	A	0.0392	0.0476	NA	1	0.00 (-Inf - Inf)	1
Global haplotype association p-value: 0.12								
**Responsive for 3 criteria**								
1	G	A	0.7745	0.66	0.8846	0.7745	1.00	---
2	T	G	0.1863	0.26	0.1154	0.9608	0.41 (0.15 - 1.12)	0.088
3	T	A	0.0392	0.08	NA	1	0.00 (-Inf - Inf)	1
Global haplotype association p-value: 0.009								
**Reduction in OCS ≥ 50%**								
1	G	A	0.7745	0.7188	0.8684	0.7745	1.00	---
2	T	G	0.1863	0.2188	0.1316	0.9608	0.59 (0.21 - 1.60)	0.3
3	T	A	0.0392	0.0625	NA	1	0.00 (-Inf - Inf)	1
Global haplotype association p-value: 0.078								
**Reduction in exacerbations ≥ 50%**								
1	G	A	0.7745	0.7604	1	0.7745	1.00	---
2	T	G	0.1863	0.1979	NA	0.9608	0.00 (-Inf - Inf)	1
3	T	A	0.0392	0.0417	NA	1	0.00 (-Inf - Inf)	1
Global haplotype association p-value: 0.26								
**Increase in %FEV1 ≥ 10% or %FEV1 ≥ 80%**								
1	G	A	0.7745	0.7162	0.9286	0.7745	1.00	---
2	T	G	0.1863	0.2297	0.0714	0.9608	0.31 (0.07 - 1.34)	0.12
3	T	A	0.0392	0.0541	NA	1	0.00 (-Inf - Inf)	1
Global haplotype association p-value: 0.048								

Freq: haplotype frequency; NA, not available; R, responder; NR, non-responder.
