# Supplementary material for: SingleNucleotide Polymorphisms as Biomarkers of Mepolizumab and Benralizumab Treatment Response in Severe Eosinophilic Asthma
Source: Int J Mol Sci. 2024 Jul 26;25(15):8139. doi: 10.3390/ijms25158139 (PMC11311889; doi:10.3390/ijms25158139)
Supplement: Supplementary file 1 [file ijms-25-08139-s001.zip › Table S8.pdf]

Table S8. Estimation of *FCER1A* rs2427837/rs2251746 haplotype frequency in patients treated with benralizumab.

	rs2251746	rs2427837	Freq	R	NR	Cumulative frequency	OR (95% CI)	p-value
**Responsive for 1 criterion**								
-	-	-	-	-	-	-	-	-
**Responsive for 2 criteria**								
1	T	G	0.7353	0.7143	0.8333	0.7353	1.00	---
2	C	A	0.2451	0.2619	0.1667	0.9804	0.58 (0.17 - 2.02)	0.4
3	C	G	0.0196	0.0238	NA	1	0.00 (-Inf - Inf)	1
Global haplotype association p-value: 0.44								
**Responsive for 3 criteria**								
1	T	G	0.7353	0.7	0.7692	0.7353	1.00	---
2	C	A	0.2451	0.28	0.2115	0.9804	0.73 (0.31 - 1.69)	0.46
3	C	G	0.0196	0.02	0.0192	1	0.82 (0.05 - 14.26)	0.89
Global haplotype association p-value: 0.76								
**Reduction in OCS ≥ 50%**								
1	T	G	0.7353	0.7344	0.7368	0.7353	1.00	---
2	C	A	0.2451	0.2344	0.2632	0.9804	1.06 (0.45 - 2.48)	0.89
3	C	G	0.0196	0.0312	NA	1	0.00 (-Inf - Inf)	1
Global haplotype association p-value: 0.38								
**Reduction in exacerbations ≥ 50%**								
1	T	G	0.7353	0.7188	1	0.7353	1.00	---
2	C	A	0.2451	0.2604	NA	0.9804	0.00 (-Inf - Inf)	1
3	C	G	0.0196	0.0208	NA	1	0.00 (-Inf - Inf)	1
Global haplotype association p-value: 0.17								
**Increase in %FEV1 ≥ 10% or %FEV1 ≥ 80%**								
1	T	G	0.7353	0.7027	0.8214	0.7353	1.00	---
2	C	A	0.2451	0.2838	0.1429	0.9804	0.50 (0.16 - 1.51)	0.22
3	C	G	0.0196	0.0135	0.0357	1	2.04 (0.12 - 36.14)	0.63
Global haplotype association p-value: 0.33								

Freq: haplotype frequency; NA, not available; R, responder; NR, non-responder.
