# Supplementary material for: SingleNucleotide Polymorphisms as Biomarkers of Mepolizumab and Benralizumab Treatment Response in Severe Eosinophilic Asthma
Source: Int J Mol Sci. 2024 Jul 26;25(15):8139. doi: 10.3390/ijms25158139 (PMC11311889; doi:10.3390/ijms25158139)
Supplement: Supplementary file 1 [file ijms-25-08139-s001.zip › Table S9.pdf]

Table S9. Estimation of *RAD50* rs11739623/rs4705959 haplotype frequency in patients treated with benralizumab.

| | rs11739623 | rs4705959 | Freq | R | NR | Cumulative frequency | OR (95% CI) | p-value |
|---|---|---|---|---|---|---|---|---|
| **Responsive for 1 criterion** | | | | | | | | |
| - | - | - | - | - | - | - | - | - |
| **Responsive for 2 criteria** | | | | | | | | |
| 1 | C | T | 0.7255 | 0.7262 | 0.7222 | 0.7255 | 1.00 | --- |
| 2 | T | C | 0.2451 | 0.2381 | 0.2778 | 0.9706 | 1.12 (0.35 - 3.60) | 0.85 |
| 3 | T | T | 0.0294 | 0.0357 | NA | 1 | 0.00 (-Inf - Inf) | 1 |
| Global haplotype association p-value: 0.54 | | | | | | | | |
| **Responsive for 3 criteria** | | | | | | | | |
| 1 | C | T | 0.7255 | 0.78 | 0.6731 | 0.7255 | 1.00 | --- |
| 2 | T | C | 0.2451 | 0.16 | 0.3269 | 0.9706 | 2.28 (0.83 - 6.28) | 0.12 |
| 3 | T | T | 0.0294 | 0.06 | NA | 1 | 0.00 (-Inf - Inf) | 1 |
| Global haplotype association p-value: 0.027 | | | | | | | | |
| **Reduction in OCS ≥ 50%** | | | | | | | | |
| 1 | C | T | 0.7255 | 0.7656 | 0.6579 | 0.7255 | 1.00 | --- |
| 2 | T | C | 0.2451 | 0.1875 | 0.3421 | 0.9706 | 2.07 (0.78 - 5.48) | 0.15 |
| 3 | T | T | 0.0294 | 0.0469 | NA | 1 | 0.00 (-Inf - Inf) | 1 |
| Global haplotype association p-value: 0.076 | | | | | | | | |
| **Reduction in exacerbations ≥ 50%** | | | | | | | | |
| 1 | C | T | 0.7255 | 0.7188 | 0.8333 | 0.7255 | 1.00 | --- |
| 2 | T | C | 0.2451 | 0.25 | 0.1667 | 0.9706 | 0.54 (0.06 - 5.02) | 0.59 |
| 3 | T | T | 0.0294 | 0.0312 | NA | 1 | 0.00 (-Inf - Inf) | 1 |
| Global haplotype association p-value: 0.7 | | | | | | | | |
| **Increase in %FEV1 ≥ 10% or %FEV1 ≥ 80%** | | | | | | | | |
| 1 | C | T | 0.7255 | 0.7297 | 0.7143 | 0.7255 | 1.00 | --- |
| 2 | T | C | 0.2451 | 0.2297 | 0.2857 | 0.9706 | 1.21 (0.44 - 3.29) | 0.72 |
| 3 | T | T | 0.0294 | 0.0405 | NA | 1 | 0.00 (-Inf - Inf) | 1 |
| Global haplotype association p-value: 0.34 | | | | | | | | |

Freq: haplotype frequency; NA, not available; R, responder; NR, non-responder.
